# A chromosome-level genome assembly for the Silkie chicken resolves complete sequences for key chicken metabolic, reproductive, and immunity genes

**DOI:** 10.1038/s42003-023-05619-y

**Published:** 2023-12-06

**Authors:** Feng Zhu, Zhong-Tao Yin, Qiang-Sen Zhao, Yun-Xiao Sun, Yu-Chen Jie, Jacqueline Smith, Yu-Ze Yang, David W. Burt, Maxwell Hincke, Zi-Ding Zhang, Meng-Di Yuan, Jim Kaufman, Cong-Jiao Sun, Jun-Ying Li, Li-Wa Shao, Ning Yang, Zhuo-Cheng Hou

**Affiliations:** 1 National Engineering Laboratory for Animal Breeding and Key Laboratory of Animal Genetics, Breeding and Reproduction, MARA; College of Animal Science and Technology, China Agricultural University, No. 2 Yuanmingyuan West Rd, 100193 Beijing, China; 2 The Roslin Institute & R(D)SVS, University of Edinburgh, Easter Bush, Midlothian, EH25 9RG UK; 3 Beijing General Station of Animal Husbandry, 100101 Beijing, China; 4 The University of Queensland, St. Lucia, QLD 4072 Australia; 5 Department of Cellular and Molecular Medicine, Department of Innovation in Medical Education, Faculty of Medicine, University of Ottawa, 451 Smyth Road, Ottawa, KIH 8M5 Canada; 6 College of Biological Sciences, China Agricultural University, 100193 Beijing, China; 7 Institute for Immunology and Infection Research, University of Edinburgh, Edinburgh, EH9 3FL UK; 8 Department of Pathology, University of Cambridge, Cambridge, CB2 1QP UK; 9 Sanya Institute of China Agricultural University, Beijing, China

**Keywords:** Evolution, Genomics, Immunogenetics

## Abstract

A set of high-quality pan-genomes would help identify important genes that are still hidden/incomplete in bird reference genomes. In an attempt to address these issues, we have assembled a de novo chromosome-level reference genome of the Silkie (*Gallus gallus domesticus*), which is an important avian model for unique traits, like fibromelanosis, with unclear genetic foundation. This Silkie genome includes the complete genomic sequences of well-known, but unresolved, evolutionarily, endocrinologically, and immunologically important genes, including *leptin*, ovocleidin-17, and tumor-necrosis factor*-α*. The gap-less and manually annotated MHC (major histocompatibility complex) region possesses 38 recently identified genes, with differentially regulated genes recovered in response to pathogen challenges. We also provide whole-genome methylation and genetic variation maps, and resolve a complex genetic region that may contribute to fibromelanosis in these animals. Finally, we experimentally show leptin binding to the identified leptin receptor in chicken, confirming an active leptin ligand-receptor system. The Silkie genome assembly not only provides a rich data resource for avian genome studies, but also lays a foundation for further functional validation of resolved genes.

## Abstract (web summary)

A chromosome-level genome for the Silkie chicken (*Gallus gallus domesticus*) provides insight into both the basis of fibromelanosis in these animals and, more broadly, unresolved genes in genome assemblies for related chicken subspecies.

## Introduction

Birds represent over 30% of known tetrapod diversity^1^, and the chicken (*Gallus gallus domesticus*) is an important model species for scientific discovery in developmental biology, genetics, virology, and immunology^2,3^. Chicken meat and eggs are consumed annually as a primary source of nutritious protein in the human diet^4,5^. While the draft Red Jungle Fowl (*Gallus gallus*, ancestor of domestic chickens) genome was first released in 2004, and continues to be updated^6–8^, several biological and evolutionarily important genes are still apparently absent in the current chicken reference genome and pan-genomes (as well as other avian genomes)^9^.

For instance, adipokines are important components of the neuroendocrine-immune network^10^, and the most studied adipokine in mammals is leptin; however, its existence in avian species has faced extensive controversies for decades^10^. Tumor necrosis factor-α (*TNF-α*) is a pleiotropic cytokine playing critical roles in host defense and was considered to be absent in avian genomes^11^. Recently, the cDNA of *TNF-α* and *leptin* have been cloned in chickens^12–16^, but the full genomic sequences are still missing from publicly available chicken reference genomes. Another important issue is the genetics of avian egg formation. The avian egg represents the most advanced amniotic egg in oviparous vertebrates, and is a successful reproductive adaptation to the desiccating terrestrial environment^17–19^. Understanding the genetics of eggshell biomineralization will be a crucial step in our understanding of the evolution of unique eggshell features. Recently, we reported two types of C-type lectin (CTL, anascalcin, *ACA-1* and *ACA-2*) in the Mallard genome^20^. However, no genomic footprint of the eggshell-specific ovocleidin-17 (*OC17*) has yet been found in the current chicken genome, even though the relevant cDNA, amino acid sequence, and protein crystal structure were obtained several years ago^17,18^. Recovering these functionally important genes in the chicken genome is an essential step in resolving various biological conundrums and is also critical for performing gene-editing experiments to confirm functions in vivo.

In addition to this, the genetic mechanisms behind the diverse phenotypes in chicken populations have not yet been fully understood. Fibromelanosis (FM), also known as dermal hyperpigmentation, stands out as one of the rare instances of skin pigmentation characteristics in chickens^21^. The extensive hyperpigmentation of dermal and connective tissues in chicken has been partially resolved^22^, but not yet addressed at the genome level^23^. Several studies have suggested that FM is caused by a complex mutation on chromosome 20, but it has not been possible to resolve the full extent of the mutation^24^ due to the unsuccessful assembly of large structural variations (SVs).

Overall, a set of high-quality pan-genomes would help identify these hidden/incomplete genes in the chicken reference genomes. Multiple pan-genomes representing different populations are necessary for a better understanding of the SVs that recover the entire gene repertoire in a species. Thousands of previously unidentified protein-coding genes and long noncoding RNAs have been recovered from the high-quality pan-genomes of human (*Homo sapiens*)^25^, pig (*Sus scrofa*)^26^, duck (*Anas platyrhynchos*)^20^, and chicken^27,28^. However, chromosome-level genome assemblies are only available for a few chicken breeds, meaning that many important genes are likely still missing or incomplete in chicken pan-genomes. For example, the Silkie chicken is considered to be an excellent model for the study of hyperpigmentation due to the large number of melanin deposits in a variety of tissues in the dermal layer of skin, sheaths of muscles and nerves, tendons, gut mesenteries, blood vessel walls, trachea, and air sacs, but lacks a chromosomal-level genome assembly^29,30^.

To address the full genomic sequences of these well-known missing genes and accurate SV of FM in chicken, we have produced a high-quality chromosome-level genome assembly of the Silkie chicken genome, and applied multi-omics methods in our analyses. Our study resolves complete genomic sequences for several genes (i.e., *leptin*, *TNF-α*, and CTL) whose existence in the avian genome was previously questioned, and we also provide the full-length MHC (major histocompatibility complex) genomic sequences and manual annotations for chicken. Besides this, we also identify the gene(s) encoding eggshell mineralization-specific C-type lectins and the potential genetic basis of FM in Silkies. A whole-genome methylation map has been produced, and millions of genome variations are identified in this assembly, which lays a foundation for further functional validation of recovered hidden genes.

## Results

### Genome assembly, annotation, and methylome

We assembled the Silkie genome by incorporating Nanopore and PacBio HiFi single-molecule real-time long-read sequences as well as sequences from high-throughput chromatin conformation capture (Hi-C) technologies (Supplementary Fig. 1). The final assembly (CAU_Silkie) contains 39 pseudochromosomes and 39 unplaced scaffolds with an N50 length of 91.5 Mb (Supplementary Table 1). The assembly accuracy and completeness were also supported by perfect matches with FISH-marker sequences^31,32^ (Supplementary Table 2) and good genomic collinearity between our assembly and the latest GRCg7b reference genome (Supplementary Figs. 2 and 3), which attest to the high contiguity and completeness of our Silkie genome assembly. To annotate genes in the Silkie genome, we curated publicly available chicken RNA sequencing (RNA-seq) datasets from 26 tissues (Supplementary Data 1) and also performed RNA-seq of different cell lines. We then used the EVidenceModeler (EVM) pipeline with assembled RNA-seq transcripts, protein homology, and de novo predictions as evidence (“Methods”). A total of 18,034 protein-coding genes were annotated in the Silkie genome (Supplementary Table 3), 17,499 (95.43%) of which have at least one annotated functional domain (Supplementary Table 4). Of these protein-coding genes, 99.43% were expressed in at least one of the 26 tissues.

We compared the Silkie genome (CAU_Silkie) with the genomes of previously available breeds^8,28^ (NCBI Accession: PRJNA693184 and PRJNA777393). The results show that the Silkie genome has assembled 14.12 Mb of previously unidentified fragments that are distributed throughout each chromosome (Supplementary Figs. 4 and 5). These assembled fragments encompass 285 protein-coding genes (Supplementary Data 2). Our results suggest that although the construction of pan-genomes is now considered a better approach for comparative genomic studies, their incorporation of low-quality individual genomes still results in a loss of information. These recently uncovered assembled fragments were also found to be much more numerous in microchromosomes than in macrochromosomes (Supplementary Fig. 4).

**Fig. 1:**
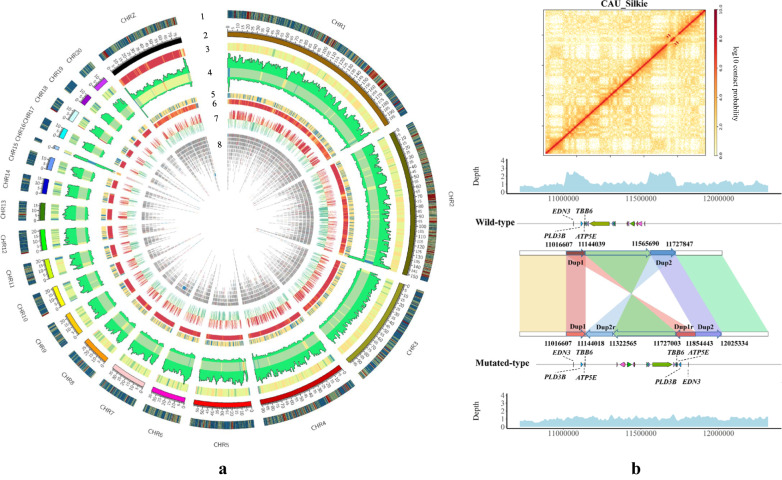
High-quality chromosome-level assembly and genome features of the Silkie chicken. **a** Genome features depicted by using 1-Mb-wide bins across the chromosomes. The tracks from the outside to the inside are: (1) Gene density; (2) The length of chromosomes; (3) 5-mc methylation level (per 1 Mb); (4) the distribution of SNVs. The outward curve is the density of SNPs, and the inward curve is the density of InDels. The middle heatmap is the density of SNVs; (5) the distribution of the noncoding RNAs; (6) the distribution of the repetitive elements; (7) the A/B compartments of chromosomes. Red color for A compartments, and green color for B compartments; (8) the distribution of SV. The gray color means that the length of the SV is less than 1000 bp, the darker the color, the longer the length. **b** The genomic collinearity for inverted duplication associated with fibromelanosis and the Hi-C heatmap of Chr20. Arrows of different colors represent the clip direction. The density curves at each end represent sequencing coverage.

To discover genomic variations, we aligned the genome sequences of a reference chicken genome [bGalGal1.mat.broiler.GRCg7b (GRCg7b)] and the resequencing reads of 15 Silkie chickens (8 males and 7 females) onto the Silkie genome. A total of 9,337,467 SNPs and 920,864 small insertions and deletions (indels, referring <=50 bp in this work) were identified (Supplementary Table 5). In addition to identifying SNPs and small indels, de novo construction of the high-quality genome provides a solid basis for the identification of complex SVs. Comparing genomes identified a total of 2799 presence/absence variations (PAVs, defined as >50 bp insertion or deletion in this work), 384 copy number variations (CNVs), 121 translocation events (including 72 intra-chromosome translocations, and 49 inter-chromosome translocations), and 70 inversion events (Fig. 1a, Supplementary Fig. 6, Supplementary Table 5, and Supplementary Data 3 and 4). A total of 385 genes were involved in these SVs (Supplementary Data 4). These SVs, especially in some complex genomic structures, allowed us to better understand if they lead to any unique characteristic of Silkie. Among all known causative mutations in Silkies, our assembly has confirmed the silky rose comb, silky-feather, crest, and polydactyly (Supplementary Figs. 7–10). Some of these traits are caused by simple point mutation (silky-feather^33^, polydactyly^34^), and some are produced by structural rearrangement (rose comb^35^, crest^36^).

Dermal hyperpigmentation is a famous trait of the Silkie chicken, and previous research suggested that an inverted duplication and junction of two genomic regions separated by more than 400 kb on Chr20 was the genetic causative mutation of FM^37^. However, due to the length of this large region and its complex variation, the complete genomic structure of this mutation, to the best of our knowledge, has not been resolved. The recently uncovered assembled genomic region showed an inverted duplication, with a length of 0.41 Mb rearranged on Chr20 of the Silkie genome. This result was different from the previous hypothesis that was based on genetic markers and short PCR results. The detailed comparison between the current assembly and the previous hypothesis is shown in Supplementary Fig. 11. To further validate the assembly of this complex region, we used Hi-C and long reads to verify the whole region in detail. There are 9745 long ONT/HiFi reads mapped on the duplication and inverted region. Among these long reads, 620 reads spanned the duplication and inverted genomic breakpoints (Supplementary Figs. 12–15, ONT/HIFI mapping reads). Further to this, we also confirmed the authenticity of this variation using resequencing and Hi-C data (Fig. 1b). The heatmap of Hi-C clearly shows the presence of inversion and duplication in this region, while the segment where the duplication is located has twice as much sequencing coverage as the wild-type genome. Our results define this complex variation and validate the use of high-quality, chromosome-level genome assemblies in understanding the molecular basis of genetic mutations.

Using Nanopore sequencing, we detected genome-wide CpG methylation sites and their frequencies. Genome-wide methylation analysis reveals genome-wide CpG methylation level. We found hypermethylation in the telomeric regions of the chromosomes and most of the hypomethylated regions on the macrochromosomes (Supplementary Figs. 16–18 and Supplementary Table 6). Genes on macrochromosomes showed higher levels of methylation than those on microchromosomes (Supplementary Fig. 19). We subjected these hypomethylated genes to functional annotation and enrichment analysis (Supplementary Figs. 20 and 21). Notably, genes within the hypomethylation window were significantly associated with bone development, which is relevant to CAU_Silkie chickens exhibiting polydactyly. After deriving the genome-wide methylation frequencies, we focused on methylation frequency of some key regions and genes. On chromosome 16 we found significantly lower methylation levels in the B regions of the MHC, as well as a lower mutation rate (Supplementary Figs. 22–24).

### Comparative genomics of the MHC region in Silkie chicken and Mallard genomes

The MHC region plays crucial roles in the innate and adaptive immune systems and has been particularly important in the strong genetic associations with resistance to a variety of infectious diseases^3,38^. The chicken MHC maintains the essential counterpart genes of the mammalian MHC as well as harboring avian-specific elements^39^. However, the current chicken MHC genomic region has not yet been fully resolved, especially considering the high polymorphism in various breeds and species. The current Silkie genome assembly data provide a complete view of a gapless MHC region in the chicken and support comparison of the chicken MHC region with that of other bird species.

**Fig. 2:**
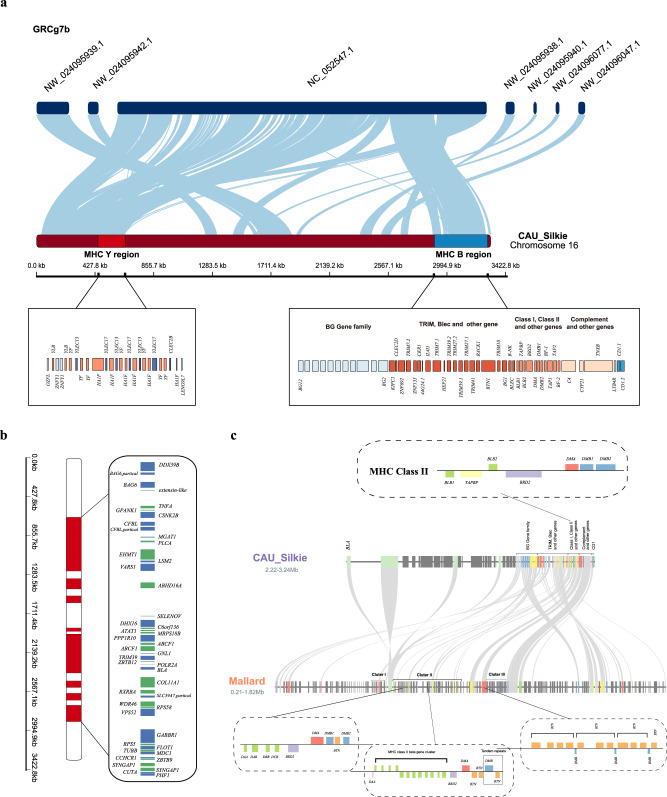
The characteristics of MHC regions of Silkie and Mallard. **a** The collinearity between Chr16 of Silkie and corresponding chromosomes in the GRCg7b genome assembly. Among them, NW_024095938.1, NW_024095939.1, NW_024095940.1, NW_024095942.1 are scaffolds that are unlocalized to Chr16, NW_024096047.1, and NW_024096078.1 are scaffolds that are unplaced to any chromosome. In the chromosome diagram of Silkie, the red area represents the MHC-Y region, and the blue area represents the MHC-B region. The corresponding box shows the gene arrangement and order in these regions. **b** The distribution of genes within the recently identified sequence on Chr16. The red part represents the position distribution of the recently identified sequence in the chromosome. The size of the square in the box represents the length of a gene, the color represents the orientation of gene transcription, blue is forward (+), and green is reverse (−). **c** The gene collinearity between the orthologous Chr16 of Silkie and Chr30 of Mallard. The gene arrangement in the dashed box represents the gene composition of three clusters in the MHC class II region in the Mallard genome. Gene models of the same color represent the same gene family members or multiple copies of one gene.

The length of Silkie Chr16 is 3.3 Mb, encompassing the corresponding chromosome in the GRCg7b reference genome along with 6 other unplaced scaffolds (478.88 kb), including the complete MHC core region (Fig. 2a and Supplementary Fig. 25). The collinearity between chromosomes in Silkie and the unplaced scaffolds helps to explain why the length of Chr16 in the Silkie genome is longer. We manually annotated 170 protein-coding genes with complete gene structure, of which 14 genes are previously unidentified and annotated on Chr16 in chicken (Supplementary Data 5). The MHC-B region of Silkie is distributed at the end of Chr16 and contains 49 genes. This region has an updated annotation of MHC class IV (BG gene family) when compared with the GRCg7b genome which represents a complete gene map of the MHC region which has been presented at the genome level. Chr16 also contains ~1.6 Mb of hitherto unknown sequence with no synteny to regions of other recently published chicken genomes or related draft genome assemblies^6,8,28^ (Supplementary Table 7). The previously unidentified sequence contains 45 protein-coding genes, of which 40 genes were freshly discovered on Chr16 of Silkie (Fig. 2b and Supplementary Table 8). It is worth noting that 31.58% of these freshly discovered genes^11^ were previously thought to be missing in birds, indicating that the Silkie genome contributes to the identification of functioning and previously undiscovered genes (Supplementary Table 8). At the same time, we found that the MHC-B and MHC-Y regions showed a low level of methylation, while the methylation level was not high in other regions with high GC content and low gene density, indicating that the related functional genes in the MHC region were subject to methylation.

Ducks are natural reservoirs of influenza A virus and are more resistant to IAV than chickens^40^. Therefore, to provide more genetic information for the chicken and duck MHC regions, we manually annotated a gapless Mallard Chr30 (containing the MHC region) from a previous study^20^ and compared chicken Chr16 and duck Chr30 chromosomes (Supplementary Data 5). At the chromosome level, the gene density of Mallard is significantly higher than that of Silkie, and both the gene length and inter-gene distance are also shorter (Fig. 2c). Mallard Chr30 contains the MHC region, and we have demonstrated that the Silkie Chr16 and Mallard Chr30 are homologous based on chromosomal collinearity (Fig. 2c). Unlike Silkie, which encodes MHC genes that are tightly distributed in the MHC-B region, these genes are loosely distributed across the entire chromosome in Mallard. Based on functional annotation, we conducted a comprehensive comparison of the iconic genes in the core region of chicken and duck MHC. The gene structure and sequences of the MHC class I region are basically consistent with the previous description (Supplementary Fig. 26)^41–44^. Class II genes of Silkie and Mallard reflect the diversity and complexity of the MHC among different species^45–47^ (Fig. 2c). Compared with Silkie, the MHC class II of Mallard contains three clusters that annotated more tandem repeat genes (Fig. 2c and Supplementary Fig. 27). The Cluster I region of Mallard and the class II region of Silkie has good collinearity. However, the TAP Binding Protein (*TAPBP*) gene in Cluster I is missing in Mallard, which indicates that MHC class I proteins in Mallard may be transported across the membrane by other means^48^. Cluster II and Cluster III genes have the characteristics of the tandem duplication of *DAB* and the tandem duplication of butyrophilin (*BTN*) and heterodimer of beta (*DMB*), respectively, which may account for increased disease resistance in Mallard and regulate the evolution of gene expression^49–51^. In addition, we have completely annotated 12 copies of the B–G antigen (*BG*) gene family in Silkie (Supplementary Fig. 28), which do not exist in the recently assembled Mallard genome and may instead have differentiated into a series of orthologous genes with less similarity during evolution^52,53^.

We reanalyzed transcriptome data derived from chicken spleen before and after infection with Marek’s Disease Virus (MDV)^54,55^, along with transcriptome data from bone marrow before and after infection with bacterial *E. coli*^56^ in order to identify differentially expressed MHC genes (DEGs) (Supplementary Data 6 and 7). We found that the expression of *BG* gene family members, *BG4*, *BG10*, and *BG12* decreased in birds infected with MDV. In response to *E. coli* infection, all members of the *BG* gene family were significantly downregulated (except *BG8* and *BG12* in Silkies). These results show that with improvements in genome assembly and annotation, a larger number of DEGs responding to disease challenges (both viral and bacterial) can be identified, which has important value for research into disease resistance in birds.

### Identification of crucial protein-coding genes that are missing in current avian genome annotations

**Fig. 3:**
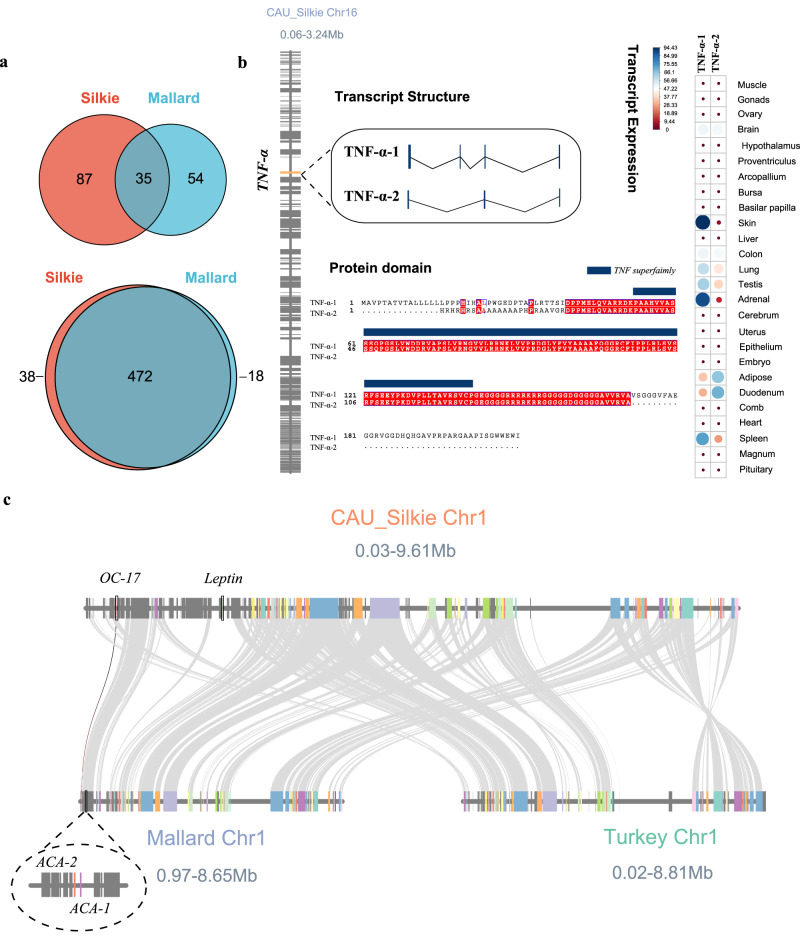
The distribution and characteristics of protein-coding genes with important functions recently annotated in the CAU_Silkie genome. **a** Venn diagram of the number of genes identified in chickens and ducks that were thought to be missing in birds. The Venn diagrams are the 176 missing genes annotated in Mallard and Silkie, and 528 high-confidence missing genes obtained through the assembly of chicken and duck multi-tissue transcriptomes. **b** The sequence and expression characteristics of *TNF-α* in Silkie. *TNF-α* contains two transcripts, composed of either 3 or 4 exons. The red part of the protein sequence represents two transcripts encoding the same amino acid, and the blue part represents tumor necrosis factor superfamily domains. In the gene expression profile, the size of the circle represents the level of expression, the unit is FPKM, and the color represents the ratio of the transcript to the total expression of the gene. **c** The genetic collinearity between the 0.03 and 9.61 Mb region on Chr1 of Silkie, Mallard, and Turkey. There are homologous genes *ACA-1* and *ACA-2* (Mallard) with *OC17* (Silkie), while the annotation of *OC17* in the turkey genome is still absent. The Silkie *leptin* gene does not show good collinearity with Mallard and Turkey.

Previous studies have suggested that a large number of protein-coding genes with important functions are missing in birds^11,57,58^. Here, we annotated 136 missing genes with complete structure in birds (Supplementary Data 8). Combined with the results of genes re-discovered using chicken and duck genome and transcriptome data, a total of 528 (92.47%) genes presumed to be missing are seen to actually be present in these bird genomes, of which 176 (30.82%) have a complete gene structure in the assembled Silkie and Mallard genomes^20,59^ (Fig. 3a and Supplementary Data 9). These results suggest that most missing genes in birds will be recovered with ongoing improvements in high-quality genome assembly and annotation.

TNF-α is a pleiotropic cytokine, and has important regulatory functions in avian energy metabolism, insulin sensitivity, and appetite, as well as disease pathogenesis^60–62^. Although the chicken *TNF-α* gene has been assembled using multiple transcriptome data, the existence of *TNF-α* in birds is still widely controversial^39,63–65^. We manually annotated the complete *TNF-α* gene structure at ~970 kb on Chr16, with two isoforms (Fig. 3b). To verify the accuracy of the assembly and annotation results, we analyzed the gene collinearity between human and chicken *TNF* genes, and this confirmed that the genome region surrounding the *TNF-α* genes in humans and chickens had good collinearity (Supplementary Figs. 29 and 30). This result indicates that this region belongs to a homologous chromosome block and has a similar structure to a segment of the human genome. The *TNF-α* gene is about 16 kb in length, and gives rise to two transcripts of 632 bp and 480 bp, encoding 210 and 156 amino acids, respectively (Fig. 3b). Previously, *TNF-α* was annotated to chromosome 14 in the chicken genome (GRCg7b, GCF_016699485.2; Gene ID: 374125), but this annotation is misleading (also named *LITAF* in the annotation). The results of multiple sequence alignment between homologous sequences of *TNF-α* showed that our annotation data corrects these annotation errors (Supplementary Fig. 31). In addition, it is worth noting that other researchers previously only obtained one transcript via transcriptome de novo assembly, while the two isoforms we annotated were obtained by both transcriptome de novo and reference assembly^14,66,67^. Among them, *TNF-α*-2 is a freshly discovered isoform consisting of 3 exons and encoding fewer amino acids. Expression profiling in multiple chicken tissues reveals that the isoform containing 4 exons (*TNF-α*-1) is predominantly expressed, and has strong tissue-specific expression, with the highest expression in the skin and adrenal gland (Fig. 3b). Therefore, here we provide evidence for the existence of 2 isoforms of *TNF-α* in birds and draw attention to a direction for re-evaluating the molecular mechanism of chicken resistance to insulin (Supplementary Fig. 31).

We also found two genes with important biological functions at the start of Chr1 (0–3 Mb). One of these is ovocleidin-17 (*OC17*), encoding a protein that is essential for chicken eggshell biomineralization^20,68^, and the other is *leptin*^69–72^ (Fig. 3c). Compared with other bird species (Mallard and Turkey), we found that *OC17* has no other copies in the chicken genome^20^. The precise annotation of *OC17* in the chicken genome will permit deeper exploration to understand its regulation and mode of gene expression, and provide support for improving eggshell quality in layer flocks. Another gene with important functions in this annotated region is the famous *leptin* gene, which consists of two exons and has extremely high (68.52%) GC content. The annotation of these genes is consistent with approximate locations generated by radiation hybrid mapping in previous studies^13,14^, indicating that a larger number of *Gallus gallus* genes with important biological functions can be accurately located in the Silkie genome, providing annotation information as the basis for gene function studies.

### The recently identified *leptin* shows binding capacity with *leptin* receptor

**Fig. 4:**
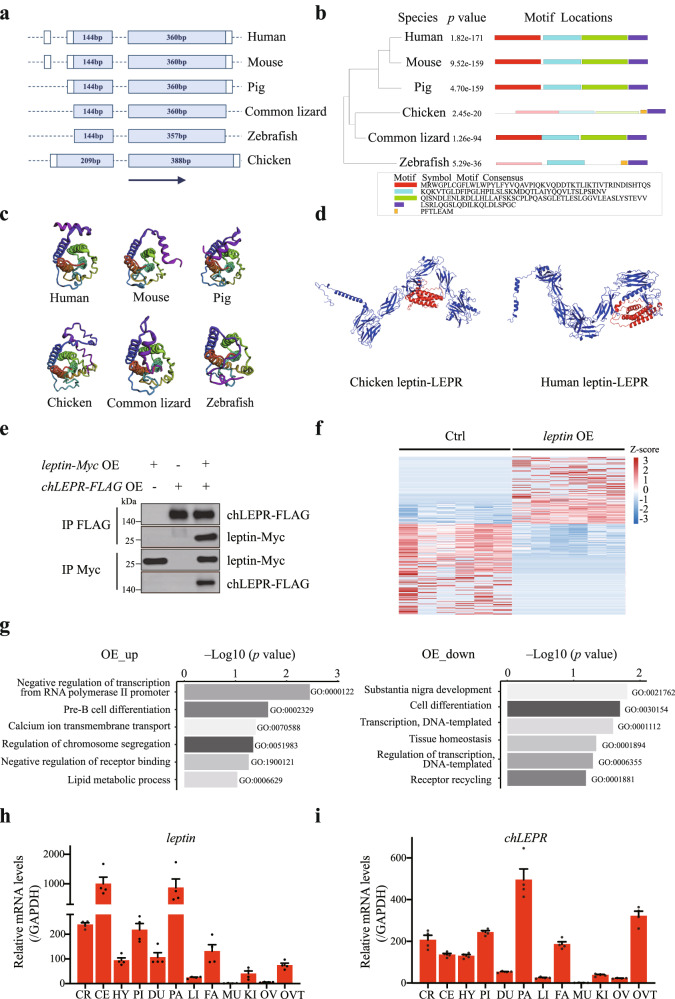
Analysis of chicken leptin sequence and functional exploration. **a**–**c** Gene and protein characteristics of *leptin* in six major vertebrates (human, mouse, pig, chicken, lizard, zebrafish), representing the diagram of gene structures (**a**), evolutionary relationships and protein motif structures (**b**), and protein structures (**c**), respectively. Human (NM_000230.3, NP_000221.1), house mouse (NM_008493.3, NP_032519.1), pig (NM_213840.1, NP_999005.1), common lizard (XM_035127222.1, XP_034983113.1), zebrafish (NM_001128576.1, NP_001122048.1). **d** Complex prediction of chicken leptin-LEPR and human leptin-LEPR. Chicken LEPR (AAF31355.2), and human LEPR (NP_002294.2). **e** Immunoprecipitation followed by immunoblotting reveals that chicken leptin interacts with chLEPR in DF-1 cells. **f** Heatmap of the differentially expressed genes in Ctrl and *leptin* over-expression DF-1 cells. **g** GO enrichment analysis of genes upregulated or downregulated in *leptin* over-expression DF-1 cells. **h**, **i** *leptin* (**h**) and *chLEPR* (**i**) expression patterns in Silkie chickens. CR cerebrum, CE cerebellum, HY hypothalamus, PI pituitary, DU duodenal mucosa, PA pancreas, LI liver, FA fat, MU muscle, KI kidney, OV ovary, OVT oviduct. Transcripts were estimated by qRT-PCR and normalized to GAPDH. *n* = 4 independent experiments. Error bars indicate mean ± SE.

While a chicken *leptin* sequence was first reported decades ago^73,74^, it has been difficult to reproduce in subsequent studies^75–77^. Two credible versions of chicken *leptin* coding sequences were released in 2016, which provide valuable baseline information for the presence of *leptin* in birds^15,78^. However, both these genomic sequences and their full-length cDNA sequences are incomplete. Here, we report a complete and accurate chicken full-length *leptin* sequence, consisting of two exons of 209 bp and 388 bp (Fig. 4a and Supplementary Fig. 32a). We then compared the chicken leptin protein sequence to that of other representative species in which leptin has also been characterized (Fig. 4a). Chicken leptin shows four similar motifs that align significantly with the mammalian and common lizard forms (Fig. 4b). Furthermore, 3D structures of leptin were modeled through RoseTTAFold^79^. As shown in Fig. 4c, leptin structures in human, mouse, pig, common lizard, zebrafish, and chicken are largely similar, which is in line with the significant sequence similarity between them. The gene encoding the chicken leptin receptor (chLEPR) sequence has already been identified and its sequence shares similarly characterized LEPR motifs with that of mammalian genes^12,80–82^. To investigate the binding function of chicken leptin to chLEPR in silico, we performed comparative protein docking experiments between chicken leptin-LEPR and human leptin-LEPR. ZDOCK^83^ was used to predict the complex structures of chicken leptin-LEPR and human leptin-LEPR (Fig. 4d). Interestingly, the docking score of chicken leptin-LEPR (66.059) was slightly higher than that of human leptin-LEPR (64.906), suggesting that chicken leptin retains its binding ability with chLEPR. We then overexpressed *leptin* and *chLEPR* in the chicken embryo fibroblast cell line DF-1, using synthetic coding sequences (Supplementary Fig. 32b, c). Immunoprecipitation experiments on lysates from the cells expressing both Myc-tagged leptin and FLAG-tagged chLEPR verified the interaction between these two chicken proteins (Fig. 4e). These Co-IP experimental results of chicken leptin and LEPR suggest that leptin plays an evolutionarily conserved role via binding to chLEPR. Moreover, we performed mRNA-seq analysis to compare the transcriptomes of wild-type cells with that of cells overexpressing leptin. Heatmaps reveal the genes significantly upregulated or downregulated in *leptin* overexpressing cells (Fig. 4f and Supplementary Fig. 32d). Gene Ontology (GO) functional enrichment analysis reveals biological processes in which the differentially expressed genes were enriched, such as lipid metabolism (Fig. 4g and Supplementary Fig. 32e).

To gain further insight into the role of *leptin* in bird species, we analyzed *leptin* and *chLEPR* expression patterns by quantitative RT-PCR. In view of the elevated GC content of the chicken *leptin* sequence, quantitative PCR primers for chicken *leptin* were identified by DNA gel electrophoresis and sequencing (Supplementary Fig. 32f). High levels of *leptin* expression were detected in the cerebrum, cerebellum, pituitary, and pancreas (Fig. 4h), which is consistent with previous reports^12,15,78^. *chLEPR* showed a similar expression pattern (cerebrum, pituitary, pancreas), in addition to high levels detected in the hypothalamus, abdominal fat, and oviduct (Fig. 4i). Taken together, we provide evidence that leptin can directly interact with chLEPR, and show that over-expression of chicken leptin can modify transcript levels of hundreds of genes. The full-length genomic and coding sequence of chicken *leptin*, therefore, provides a solid basis for further functional investigations.

## Discussion

The high-quality chromosome-level assembly of the Silkie genome provides 14.12 Mb of previously unidentified sequence that is not present in the red jungle fowl genome (Supplementary Data 10). The high GC content and some repetitive elements of the “hidden” genes previously caused the failed assembly of these genes^11^. Between 3.5% and 11.3% of genomic regions in the current VGP (Vertebrate Genomes Project) assemblies are missing across species, as was found in a recent study^84^. As genome quality improves, we predict that more presumed-missing genes may be annotated in other bird species. Several undetected genes have been recovered via transcriptome assembly methods and also had their chromosome position confirmed by FISH-mapping^31,32^. Our Silkie assembly provides a marked advance, in that we have identified ‘hidden’ and controversial genes, and confirmed the previous FISH-mapping results. One major finding from the Silkie genome sequence is the 1.6 Mb sequences located in the MHC region (a genome region never assembled completely until now), which harbors 38 freshly identified protein-coding genes in chicken. In addition, *TNF-*α, which plays multiple roles in avian energy metabolism and disease pathogenesis^14^, is located close to the MHC-Y region with a high GC content. In addition to this full-length genomic region, we also reveal that *TNF-α* has two isoforms, which extends current knowledge. Several recently identified *BG* gene family members were found to be differentially expressed between infection and control groups after previous MDV (viral)^54,55^ and *E. coli* (bacterial)^56^ infection data were reanalyzed, which could be used in subsequent functional studies^54,56^. In addition, we have obtained the complete gene structure of protein-coding genes in the MHC-Y region using manual annotation with the improvement of genome assembly quality, providing necessary data for exploration of the role of the MHC-Y region in avian immunity. Similarly, we found that the MHC-Y region has more repeat sequences than the MHC-B region, and that the MHC-Y class I elements in the region are highly polymorphic^85–88^. Comparison of the current Silkie and Mallard genome assemblies^20^ will also accelerate progress in comparative immunology between chicken and duck. Our results show that the Mallard MHC class II region contains three gene clusters that are annotated as more tandemly repeated genes, and that the *TAPBP* gene is missing in ducks. Further genome-wide comparison between the chicken and Mallard immunome will be essential for understanding differences in the host-pathogen interactions between these two economically important species.

The assembly of the Silkie genome has provided several pieces of evidence to evaluate the presence or absence of various well-known genes in the avian genome. In addition, it offers valuable insights into the comparative aspects of endocrinology, immunology, and reproduction between birds and mammals. Mammalian *leptin* has been identified for over twenty years^89^, yet the many attempts to identify and verify avian *leptin* sequences have not easily been accepted due to experimental issues related to its high GC content and high repetitiveness^1,15,78,80,90^. Our study successfully deciphered the entire genomic and cDNA sequences of leptin, providing additional evidence for its presence in birds. More importantly, based on the complete *leptin* sequence, we have demonstrated that chicken leptin shares similar protein structure and amino acid motifs with the mammalian and lizard versions (Fig. 4b, c), and we experimentally verified that chicken leptin interacts with the chicken leptin receptor. It is intriguing that chicken *leptin* was found to be highly expressed in the brain and digestive tract, whereas human *leptin* is most highly expressed in adipose tissue^69,91^. These distinct expression patterns indicate that leptin may play different roles in birds and mammals. Interestingly, genes involved in lipid metabolism appear to be upregulated as a consequence of *leptin* over-expression. It will thus be interesting to investigate whether leptin indeed modulates energy homeostasis in chickens, and other potential functions also need to be further investigated in birds. For example, the expression in the chicken brain is intriguing. Furthermore, due to the low expression level of leptin in chicken-immortalized cell lines, in vitro cell experiments are inadequate for the characterization of its function. Hence, the generation of *leptin*-edited chickens will be essential for further progress. We recognize that a recent publication described a high-quality Huxu chicken genome assembly^8,28^, and while comparison to this assembly was out of scope for the current study, high-quality annotations are critically important for discovering missing or incomplete avian genes. In general, as more high-quality pan-genomes are released, more of these missing or incomplete genes can be recovered, eventually revealing more functional mutations. In our case, the high-quality Silkie chicken assembly solves FM traits which had not been investigated by large-scale chicken pan-genome assemblies, especially based on the second-generation whole-genome sequencing data, and insufficient third-generation data. Overall, this line of research will facilitate our future understanding comparative molecular endocrinology and genetic functional divergence during avian evolution.

## Methods

### Sample collection

We randomly selected adult Silkie birds from flocks that were raised under standard feeding regimes for this study (eight males and seven females). Fresh blood was used for PacBio and Nanopore sequencing, and breast muscle tissue for Hi-C sequencing. Fifteen adult Silkies (eight males and seven females) were fed with the same diet and maintained under the same lighting conditions, and fresh blood was collected for resequencing. At 20 weeks of age, birds were euthanized by cervical dislocation and dissected. Fresh tissues from the cerebrum, cerebellum, hypothalamus, pituitary, duodenal mucosa, pancreas, liver, fat, muscle, kidneys, ovaries, and oviduct were processed for qRT-PCR. Blood samples from the wing vein of 15 Silkies were also collected and stored at −20 °C before DNA extraction. Information relating to sequencing samples is shown in Supplementary Data 1. All experiments with birds were performed under the guidance of ethical regulations, and approved by the Animal Care and Use Committee of China Agricultural University, Beijing, China (permit number: SYXK 2007–0023).

### Library construction and sequencing

DNA from the same female Silkie bird was used to generate PacBio, Nanopore sequencing libraries. More than 20 µg of sheared DNA was subjected to size selection by the BluePippin system, and ~20 kb Sequel SMRT bell libraries were prepared according to the protocol provided by the Pacific Biosciences Company (PacBio). Four single-molecule real-time (SMRT) cells were run on a PacBio RSII system using P6-C4 chemistry, with ~774 Gb (~645X) Subreads data generated.

For Nanopore sequencing, DNA was sequenced to 85× coverage on an Oxford Nanopore GridIon sequencer following the manufacturer’s instructions. Guppy (v4.0) was used for base calling and output to FASTQ files.

A Hi-C library was constructed from breast muscle, extracted from the same sequenced animal. Briefly, nuclear DNA was cross-linked in situ in 2% formaldehyde before the nuclei were extracted and then digested by *Dpn*II restriction endonuclease. The sticky ends of the digested fragments were biotinylated, diluted, and ligated randomly. The biotinylated DNA fragments were enriched to construct the sequencing libraries, and the sequencing of these libraries was conducted on the Illumina NovaSeq 6000 platform. Finally, a total of 707 million (~176×) 150 bp paired-end reads were produced.

#### Genome assembly and polishing

A total of 774 Gb (~645× coverage) of subreads were generated from the PacBio Sequel II platform and were converted to the respective CCS (Circular Consensus Sequence) reads using PBCCS (v5.0.0) [https://github.com/PacificBiosciences/pbbioconda]. Briefly, clean HiFi subreads were assembled into contigs by hifiasm^92^ (0.13-r307) with default parameters. We generated 43.12 Gb PacBio HiFi long reads (39× coverage) and 93.44 Gb (84x coverage) Oxford Nanopore long reads (Supplementary Tables 9–11). To correct systematic errors of Nanopore sequencing, FMLRC2^93^ (v0.1.1) (“-m 3 -C 10”) was used with HiFi reads. Subsequently, corrected ONT long reads were set as input for NextDenovo (v2.3.1) (https://github.com/Nextomics/NextDenovo) with parameters “task = all;input_type = raw;read_cutoff = 1k;seed_cutoff = 32459 #;minimap2_options_cns = -x ava-ont -t 8 -k17 -w17”. Variants that were considered the result of sequencing errors were polished using Nextpolish^94^ with default parameters using Nanopore reads. Quickmerge^95^ was used in series with default parameters to scaffold the hybrid assembly using PacBio and Nanopore scaffolds/contigs twice. The duplicates and redundant haplotypes were removed using purge_dups (v1.2.5). The hybrid assembly with a contig N50 of 72.4 Mb (Supplementary Table 12) was anchored into 39 pseudochromosomes using Juicer (v.1.6.2)^96^ and SALSA2 (v2.2)^97^ in combination with the Hi-C reads (176× coverage) (Supplementary Tables 13 and 14 and Supplementary Fig. 33). To improve the assembly quality, pbjelly2 and TGS-GapCloser^98^ were used to fill the pseudo-chromosome gaps.

We assessed the completeness of the Silkie genome assembly by searching for 8338 single-copy avian genes using BUSCO (BUSCO v.5 and the aves_odb10 database)^99^. The final assembly of 39 pseudochromosomes was obtained with 96.64% (8,058/8,338) completeness in conserved single-copy protein-coding sequences (Supplementary Fig. 34). We aligned FISH-marker sequences obtained from previous studie2^9,30^ to the pseudochromosomes using Blast^31,32,100^.

#### RNA sequencing and gene expression analysis

To facilitate gene model annotation and to capture diverse gene expression, we extracted RNA from multiple cell lines of chicken (Supplementary Data 1). Cells from one well of a six-well plate with about 90% cell confluence per sample were used. Total RNAs were extracted using the RNeasy Plant Mini Kit (Qiagen, USA), following the manufacturer’s protocol. RNA quality was assessed using an Agilent Bioanalyzer 2100 (Agilent Technology, USA), and RNA samples with RNA integrity number (RIN) > 9.0 were used for cDNA library preparation. Illumina libraries for RNA sequencing were prepared as previously described. The cDNA libraries for Illumina sequencing were prepared using the SureSelect Strand-specific RNA library kit (Agilent Technology, USA), according to the manufacturer’s instructions, and sequenced using an Illumina HiSeq 4000 sequencer (Illumina, San Diego, USA) to obtain paired-end reads with an average length of 150 bp and over 8 Gb sequencing reads for each of the multiple samples.

We also curated all publicly available chicken RNA sequencing (RNA-Seq) datasets from 26 tissues (Supplementary Data 1) to facilitate the genome annotation. All RNA sequencing data were analyzed according to a standard process. Trimommatic^101^ (v0.40) was used to remove the adapters, poor quality reads (base quality score >30), and reads with N base (where the number of invalid bases accounted for more than 5%) from the raw RNA-Seq data. The clean reads were de novo assembled to transcripts by Trinity^102^ (v2.13.2) with parameters for gene model annotation (--max_memory 500 G, --CPU 200). For expression analysis, we used HISAT2^103^ software (v2.1.1) to map clean reads to the Silkie reference genome with default parameters. The expression level for protein-coding genes (RPKM, TPM, and expression count data) was obtained using HTSeq^104^ software (v0.11.2). In addition, the RNA-Seq data were mapped to the cDNA sequences of the Silkie genome by Salmon and the expression of each isoform was determined. Differentially expressed genes between different samples were identified using the DEseq2^105^ (v1.32.0) package with the following standards: FDR < 0.05 and absolute fold change >1.5, and the read counts of all genes were used as the input data for the analysis. The DAVID database (2021 Update)^106^ was implemented to analyze functional categories (using the chicken genome annotation as a background) and the association between genes and the corresponding Gene Ontology (GO) classification.

### Protein-coding gene annotation

Gene models were annotated using the EVidenceModeler (EVM) genome annotation pipeline^107^ (v2.31.8), which integrates both ab initio gene predictions generated by Braker2^108^ (v2.1.6), AUGUSTUS^109^ (v3.3.2), and SNAP (v.2013-11-29), as well as homology evidence including protein sequences in the SwissProt database (release 2018_12), in addition to a de novo transcriptome assembly generated from RNA-seq data using Trinity^110^ (v2.8.3). The gene models were further refined using PASA (v2.3.3)^107^.

### Noncoding RNA gene annotation

Noncoding RNA species including miRNA, tRNA, rRNA, and snRNA were annotated using several methods. tRNA species were predicted using tRNAscan-SE^111^ (v1.3.1) with default parameters. rRNA species were identified by mapping chicken rRNA sequences to the Silkie chicken genome using BLASTN-short (v2.2.28). miRNA and snRNA were annotated by scanning Rfam^112^ against the genome and passing the results into Infernal^113^ (v1.1.3) with default parameters. Noncoding RNAs including 254 microRNAs (miRNA), 171 small nuclear RNAs (snRNA), and 290 ribosomal RNAs (rRNA) genes were also predicted in the Silkie genome (Supplementary Table 15).

### Annotation of repeats and transposable elements

Repeats were analyzed with a method combining de novo structure analyses and homology comparison. First, RepeatModeler2^114^ was employed to construct the repeat elements library. Full-length LTR-RTs were identified using LTR_FINDER with the following parameters: -D 15000 -d 1000 -L 700 -l 100 -p 20 -C -M 0.9. Repeat regions were then annotated by RepeatMasker (v4.0.7)^115^ using de novo prediction in conjunction with the reference library. Repetitive elements accounted for 16% of the genome, most of which were transposable elements (Supplementary Table 16).

#### Comparative genomic analysis

We obtained seven other chicken assemblies (GCA_024206055.1(Huxu), GCA_024652995.1(White Leghorn), GCA_024653025.1(Silkie), GCA_024652985.1(Rhode Island Red), GCA_024653045.1(Houdan), GCA_024653035.1(Cornish), and GRCg7b) from NCBI. All assemblies were aligned to CAU_Silkie using Winnowmap2 (v2.03)^116^ with “-a -x asm20 --cs -r 2000 -k 15”. Svim-asm(v1.0.3)^117^ was used to detect variations with haploid mode. For detecting hitherto unknown assembled sequences, we used SURVIVOR (v1.0.7)^118^ with parameters “1000 7 1 1 0 50”. Absence was defined by DEL (length>50 bp) variation in the resulting VCF file.

### Synteny analysis

Synteny analysis of genomes was performed via whole-genome alignment using MUMmer4^119^ (v4.00beta2) for CAU_Silkie versus GRCg7b. Alignment of the genomes was performed using NUCmer (–c 1000), and then the alignment block filter was performed using a delta-filter with one-to-one alignment mode (-1 -i90 -l10000). The homologous genes were analyzed by the MCScanX package^120^ with default settings, except for gap_penalty -3. Syntenic blocks were defined as those with at least five syntenic genes.

### Genomic resequencing and variant calling

Genomic DNA was extracted from the blood samples. At least 5 μg DNA was used for library construction using the Illumina TruSeq DNA Sample Prep Kit (Illumina, CA, USA). DNA was isolated using the DNeasy Blood & Tissue Kit (QIAGEN, ON, Canada). The purified genomic DNA was mechanically disrupted using Bioruptor (Diagenode Inc., NJ, USA) to generate ~300 bp inserts. The DNA fragments were subjected to end repair and A addition to the 3’end, followed by amplification using the Thermal cycler 1000 (Bio-Rad). The purified library was subjected to quality control using StepOne Plus (Applied Biosystems, MA, USA). Finally, the Nova-seq6000 platform (Illumina, CA, USA) was used to generate paired-end sequencing data with a genome coverage of at least 30×.

The variant calling was performed using the Speedseq pipeline^121^. After trimming of low-quality bases using Trimmomatic (version 0.32), the clean data for 15 Silkies and 11 Rhode Island Red birds obtained from the Bioproject PRJEB44038 were mapped to the Silkie genome and GRCg7b using BWA software (version 0.7.10- r789)^122^. All the unique mapping data were extracted to identify SNPs and small InDels using freebayes (v0.9.21) and Samtools^123^ (version 0.1.19) programs. Variants were removed with QualByDepth (QD) < 4.0, 300 > depth >2200, Quality <30, mapping quality (MQ) < 40.0, MQRankSum < -10, Read- PosRankSum < -7.0, Fisher Strand > 60.0, ReadPosRankSum >7, BaseQRankSum < -6, BaseQRankSum > 6”. Cluster Size and ClusterWindowSize were set to 4 and 10, respectively. SVs (Inversions, Translocations, Duplications, Insertions, and deletions) were identified by extracting unaligned regions between Silkie chicken and GRCg7b (GCA_016699485.1) from the genomic alignment using MUMmer4 (v4.00beta2)^119^ and syri (v1.5)^124^. All genome resequencing data (15 Silkies and 11 Rhode Island Red birds) were mapped to the CAU_Silkie and GRCg7b genomes to confirm the potential presence/absence of variations. The one-tailed t-test was used to determine whether the coverage was significantly different between the two genomes. Other structural variants (inversions and duplications) were verified by Delly (v0.9.1) and manual checking.

#### Resolution of the fibromelanosis locus

Nanopore reads and HiFi reads mapped to chr20:10716608-12027847 were extracted to assemble sequences. First, we found soft-clip reads at dup1 3’, dup2 5’(dup: duplication). We selected reads from dup1 5’ (subtract soft-clipped reads), dup1 3’ (soft-clipped reads), dup2 5’ (subtract soft-clipped), dup2 3’ (soft-clipped reads), and other normal reads corresponding to wild-type genomic position as shown in Fig. 1. Reads were assembled by Flye^125^. Likewise, reads from dup1 5’ (soft-clipped reads), dup1 3’ (subtract soft-clipped reads), dup2 5’ (soft-clipped reads), and dup2 3’ (subtract soft-clipped reads), and other normal reads were selected, corresponding to Fig. 1 wild-type genomic position. Reads were again assembled by Flye. Resulting contigs the were merged by quickmerge, and then integrated into the genome.

#### Validation of known causal variations in the assembled genome

The causal variations for the Silky-feather^33^, Polydactyly^34^, rose comb^35^, and crest^36^ have been identified in previous studies. In order to validate these known variations in the current assembly, we downloaded the assemblies (Galgal3, (GCF_000002315.1) and Galgal4, (GCA_000002315.2) from GenBank and extracted genomic sequences from the corresponding chromosomal regions for these known causal variations. Finally, BLAST^126^ and MUMMER^127^ were used to align genomic sequences from the selected regions for these traits. The results were visualized by LINKVIEW (https://github.com/YangJianshun/LINKVIEW) and ESPript^128^.

#### DNA methylome analysis

To measure CpG methylation in Nanopore data, we used the Nanopolish pipeline from METEORE (v1.0.0)^129^. Minimap2 was used for reads mapping with the “-a -x map-ont” parameter. Nanopolish employs a Hidden Markov Model (HMM) on the Nanopore current signal to distinguish 5-methylcytosine from unmethylated cytosine. Methylation call filtering, CpG site splitting, and frequency calculating were completed by scripts in Nanopore-methylation-utilities (https://github.com/timplab/nanopore-methylation-utilities). We used a log-likelihood ratio of 2.0 as a threshold for calling methylation. CpG sites with log-likelihood ratios greater than 2.0 (methylated) or less than −2.0 (unmethylated) were considered high-quality and included in the analysis. Reads that did not have any high-quality CpG sites were excluded from the subsequent methylation analysis. Mean frequency was calculated in 50-kbp windows. High-frequency (top 1%) and low-frequency (lowest 1%) windows were picked to find gene overlaps for enrichment analysis (https://maayanlab.cloud/Enrichr/). We selected genes that were longer than 4kbp, and more than 10kbp from a chromosome end, and calculated methylation frequency for 1kbp windows within 10kbp upstream and 10kbp downstream regions of each gene. The chi-square test was used for methylation type and chromosome type. A pairwise test (Games–Howell) was used for the frequency of methylation at each GC site across the entire Chr16. Wilcoxon test was used for methylation frequency of gene body regions on macro- and microchromosomes.

### Strategy to identify missing genes

We used the annotated transcripts of the Silkie genome to find sequences homologous to any of the 571 genes previously thought to be missing from the bird genome, of which 274 were thought to be missing from all avian genomes^6,11,58,59,130^. The human protein sequences of the corresponding missing genes were used as query sequences to search for homologies in the recently assembled Silkie genome using the best-reciprocal Blast algorithm. We manually checked each matched candidate sequence based on the list of missing genes to distinguish matching paralogous products and alignment errors.

### Manual annotation of the MHC region in Silkie and Mallard

Chr16 contains the MHC region, which has the characteristics of a large number of repetitive sequences and genes. Manual annotation is a powerful method to obtain the precise gene map of the chromosome. We deployed the Apollo^131^ (v2.6.1) gene annotation editing platform to manually annotate the protein-coding genes of Chr16 in Silkie and Chr30 in Mallard^20^. Based on the gene model annotated by the EVM^107^ process, the full-length transcriptome and RNA-Seq of multiple tissues, and ab initio predictive gene models from multiple sources were loaded in the track as the reference evidence for correcting the gene structure. The structure of the gene models was adjusted according to the gene features such as conserved protein functional domains and codons, to ensure that the corrected genes possessed a complete gene structure. MDV or *E. coli* infection are very common in chicken production, and we hypothesized that previously unidentified genes could be discovered based on improved annotation of the chicken MHC region. The RNA-seq data relating to MDV infection of two genetically divergent white Leghorn lines (6_3_ and 7_2_) can be found in NCBI under accession number PRJNA344896^54^, while the RNA-seq data relating to *E. coli* infection can be found under accession number PRJNA279487^56^. We reanalyzed these transcriptomic datasets to identify differentially expressed MHC genes (DEGs). The RNA-Seq analysis method was previously described in “RNA-Seq analysis method” section.

### Cell line and plasmids

To over-express *leptin* and chLEPR in chicken cells, the chicken embryo fibroblast cell line, DF-1, and the immortalized chicken preadipocyte cell line, ICP-1 were cultured in DMEM/F12 medium (Gibco #31330095) supplemented with 10% fetal bovine serum (Gibco #10099141) at 37 °C. They are two of the few chicken-immortalized cell lines suitable for in vitro culture. *Leptin* and *chLEPR* plasmids were constructed by ligating cDNA sequences into the pCDNA3.1 vector. Due to the difficulty in obtaining the intact coding sequence by PCR amplification of cDNA from chicken tissues with high expression, we synthesized the *leptin* coding sequences to optimize the codon usage and reduce the GC content. Plasmids were transfected into cells by FuGENE reagent (Promega #E2311).

### *Leptin* coding sequence verification

As the full-length *leptin* failed to be amplified with alternative primers, several short overlapping fragments were PCR-amplified from Silkie brain cDNA, sequenced and conceptually spliced together. Primer sequences are provided in Supplementary Table 17.

#### Analysis of leptin and LEPR protein structures

Robetta (https://robetta.bakerlab.org) was used to submit the aligned polypeptides of human (NP_000221.1), house mouse (NP_032519.1), pig (NP_999005.1), common lizard (XP_034983113.1), and zebrafish (NP_001122048.1) leptins and chicken (AAF31355.2), human (NP_002294.2) LEPRs to the RoseTTAFold protein modeling server^79^. ZDOCK (https://zdock.umassmed.edu)^83^ was used to predict the complex structures for leptin and LEPR. Limited by the PDB file size for ZDOCK submission, human LEPR (1-834 AA) and chicken LEPR (1–889 AA) sequences which contain the entire extracellular region were selected for protein docking. The motif analysis of *leptin* protein sequences of human (NP_000221.1), mouse (NP_032519.1), pig (NP_999005.1), common lizard (XP_034983113.1), and zebrafish (NP_001122048.1) was completed using MEME (version 5.5.0)^132^ software with default parameters.

### RNA isolation and RT-qPCR

RNA was extracted from 12 different tissues including the cerebrum, cerebellum, hypothalamus, pituitary, duodenal mucosa, pancreas, liver, fat, muscle, kidney, ovary, and oviduct in order to extract RNA to detect the expression levels of leptin and LEPR in Silkie chickens. Chicken tissues of about 50–100 mg per sample were ground at 0 °C. For cells, one well of a six-well plate with about 90% cell confluence per sample was used. Total RNA in cells or tissues was isolated by the TRIzol (Thermo #15596026) method. cDNA was synthesized by reverse transcription kit (Takara #RR037A). RT-qPCR was performed using SYBR Green PCR Master Mix (Takara # RR820A). For quantification, mRNA levels were normalized to *GAPDH*. Primer sequences for quantitative RT-PCR are listed in provided in Supplementary Table 18.

#### Immunoblotting

Immunoblotting was performed to determine whether chicken leptin and LEPR proteins were successfully expressed in DF-1 cells. Around 1 million cells per sample were washed with PBS buffer and resuspended in RIPA buffer (Solarbio #R0010) on ice for 10 min. The cell lysates were collected in tubes containing SDS loading buffer (Solarbio #P1040) and boiled at 95 °C for 10 min. Samples were separated by SDS-PAGE and transferred onto a PVDF membrane (Bio-Rad). After blocking with 5% milk-TBST, the membranes were probed with designated primary antibodies (Anti-Myc antibody (CST #2276, 1:1000 dilution), anti-FLAG antibody (Sigma #F7425, 1:2000 dilution), anti-GAPDH antibody (Proteintech #10494-1-AP, 1:1000 dilution)) and secondary antibodies (anti-Mouse IgG antibody (Abbkine #A25112, 1:2000 dilution), anti-Rabbit IgG antibody (Proteintech #SA00001-2, 1:3000 dilution). Immunoreactive bands were visualized with the enhanced chemiluminescence method, using the Syngene G: BOX Chemi XX6 chemical luminescence imaging system.

#### Immunoprecipitation

Immunoprecipitation was performed to detect whether chicken leptin and LEPR proteins can bind to each other. Cells in 10 cm dishes with 90% confluence were washed with PBS buffer, resuspended in 1 ml lysis buffer (50 mM Tris-HCl pH 8.0, 137 mM NaCl, 1% Triton X-100, 1 mM EDTA, 10% glycerol, proteinase inhibitor), and then incubated on ice for 30 min. The cell lysate was centrifuged at 20,000 × g for 15 min. The supernatant was transferred into a new tube and rotated at 4 °C overnight in the presence of the designated antibody. Anti-FLAG magnetic beads (Sigma #M8823, 40 µl per sample) or anti-Myc magnetic beads (Bimake #B26302, 20 µl per sample) were utilized. After binding, the beads were washed three times with lysis buffer and boiled in 50 µl SDS loading buffer (Solarbio, #P1040) at 95 °C for 10 min. Immunoblotting was performed as described in the previous section.

### Statistics and reproducibility

Differentiation assays have been performed at least three times, and all attempt at replication were successful (detail are indicated on figures/results). In RNA-seq analysis, each design has six biological replicates, which eliminates intra-group errors and improves the accuracy of the results. The sequencing data is 8 G/sample to ensure that the sequencing is saturated. DNA resequencing sequencing depth is between 10 and 30× to ensure comprehensive and accurate detection of variation. All the RT-qPCR experiments were performed at least three times, and one-way ANOVA with post hoc tests was used for statistical analyses. Analyses were performed using GraphPad Prism (GraphPad Software, Inc.). Sample sizes constituted *n*  ≥  3 biological replicates per group.

### Reporting summary

Further information on research design is available in the Nature Portfolio Reporting Summary linked to this article.

## Supplementary information

Supplementary Information

Description of Additional Supplementary Files

Supplementary Data 1

Supplementary Data 2-10

Supplementary Data 11

Reporting Summary

## Data Availability

All genome assembly, genomic resequencing, and RNA-Seq datasets reported in this study have been deposited in GenBank (NCBI) under accession numbers PRJNA805080 and PRJNA827662. This WGS project of Silkie chicken has been deposited at DDBJ/ENA/GenBank under the accession JAKZEP000000000. The version described in this paper is version JAKZEP010000000. The public RNA-seq data for Silkie genome annotation is consistent with the description of tables in Supplementary Data 8. The annotation information was deposited in the Figshare database (10.6084/m9.figshare.22115024.v4). The uncropped images for western blotting are included in Supplementary Fig. 35. Source data are included in Supplementary Data 11.

## Code availability

We utilized publicly available software in all analyses. The analysis code has been deposited in the repository: 10.5281/zenodo.10077021^133^.

## References

[1] Friedman-Einat, M. & Seroussi, E. Avian leptin: bird’s-eye view of the evolution of vertebrate energy-balance control. *Trends Endocrinol. Metab.* **30**, 819–832 (2019). 31699239 10.1016/j.tem.2019.07.007

[2] International Chicken Genome Sequencing C. Sequence and comparative analysis of the chicken genome provide unique perspectives on vertebrate evolution. *Nature* **432**, 695–716 (2004). 15592404 10.1038/nature03154

[3] Tregaskes, C. A. & Kaufman, J. Chickens as a simple system for scientific discovery: the example of the MHC. *Mol. Immunol.* **135**, 12–20 (2021). 33845329 10.1016/j.molimm.2021.03.019 PMC7611830

[4] Hjellnes, V., Slizyte, R., Rustad, T., Carvajal, A. K. & Greiff, K. Utilization of egg-laying hens (Gallus *Gallus domesticus*) for production of ingredients for human consumption and animal feed. *BMC Biotechnol.* **20**, 22 (2020). 32375769 10.1186/s12896-020-00618-x PMC7204061

[5] Bennett, C. E. et al. The broiler chicken as a signal of a human reconfigured biosphere. *R. Soc. Open Sci.* **5**, 180325 (2018). 30662712 10.1098/rsos.180325 PMC6304135

[6] Warren, W. C. et al. A new chicken genome assembly provides insight into avian genome structure. *G3 (Bethesda)* **7**, 109–117 (2017). 27852011 10.1534/g3.116.035923 PMC5217101

[7] Wang, M. S. et al. 863 genomes reveal the origin and domestication of chicken. *Cell Res.* **30**, 693–701 (2020). 32581344 10.1038/s41422-020-0349-y PMC7395088

[8] Huang, Z. et al. Evolutionary analysis of a complete chicken genome. *Proc. Natl. Acad. Sci. USA* **120**, e2216641120 (2023). 36780517 10.1073/pnas.2216641120 PMC9974502

[9] Smith, J. et al. Fourth report on chicken genes and chromosomes 2022. *Cytogenet Genome Res.* **162**, 405–528 (2022). 36716736 10.1159/000529376 PMC11835228

[10] Mellouk, N. et al. Chicken is a useful model to investigate the role of adipokines in metabolic and reproductive diseases. *Int. J. Endocrinol.* **2018**, 4579734 (2018). 30018639 10.1155/2018/4579734 PMC6029501

[11] Lovell, P. V. et al. Conserved syntenic clusters of protein coding genes are missing in birds. *Genome Biol.* **15**, 565 (2014). 25518852 10.1186/s13059-014-0565-1 PMC4290089

[12] Seroussi, E. et al. Avian expression patterns and genomic mapping implicate leptin in digestion and TNF in immunity, suggesting that their interacting adipokine role has been acquired only in mammals. *Int. J. Mol. Sci.* **20**, 4489 (2019). 10.3390/ijms20184489 PMC6770569 31514326

[13] Seroussi, E. et al. Mapping of leptin and its syntenic genes to chicken chromosome 1p. *BMC Genet.* **18**, 77 (2017). 28793857 10.1186/s12863-017-0543-1 PMC5550943

[14] Rohde, F. et al. Characterization of chicken tumor necrosis factor-alpha, a long missed cytokine in birds. *Front. Immunol.* **9**, 605 (2018). 29719531 10.3389/fimmu.2018.00605 PMC5913325

[15] Seroussi, E. et al. Identification of the long-sought leptin in chicken and duck: expression pattern of the highly GC-rich avian leptin fits an autocrine/paracrine rather than endocrine function. *Endocrinology* **157**, 737–751 (2016). 26587783 10.1210/en.2015-1634

[16] Dalman, M. R., Liu, Q., King, M. D., Bagatto, B. & Londraville, R. L. Leptin expression affects metabolic rate in zebrafish embryos (*D. rerio*). *Front. Physiol.* **4**, 160 (2013). 23847542 10.3389/fphys.2013.00160 PMC3696835

[17] Hincke, M. T. et al. The eggshell: structure, composition and mineralization. *Front. Biosci.* **17**, 1266–1280 (2012). 10.2741/3985 22201802

[18] Erben, H. K., Hoefs, J. & Wedepohl, K. H. Paleobiological and isotopic studies of eggshells from a declining dinosaur species. *Paleobiology* **5**, 380–414 (1979).

[19] Mann, K. & Siedler, F. The amino acid sequence of ovocleidin 17, a major protein of the avian eggshell calcified layer. *Biochem. Mol. Biol. Int.* **47**, 997–1007 (1999). 10410246 10.1080/15216549900202123

[20] Zhu, F. et al. Three chromosome-level duck genome assemblies provide insights into genomic variation during domestication. *Nat. Commun.* **12**, 5932 (2021). 34635656 10.1038/s41467-021-26272-1 PMC8505442

[21] Faraco, C. D., Vaz, S. A., Pastor, M. V. & Erickson, C. A. Hyperpigmentation in the Silkie fowl correlates with abnormal migration of fate-restricted melanoblasts and loss of environmental barrier molecules. *Dev. Dyn.* **220**, 212–225 (2001). 11241830 10.1002/1097-0177(20010301)220:3<212::AID-DVDY1105>3.0.CO;2-9

[22] Dorshorst, B. et al. A genomic duplication is associated with ectopic eomesodermin expression in the embryonic chicken comb and two duplex-comb phenotypes. *PLoS Genet.* **11**, e1004947 (2015). 25789773 10.1371/journal.pgen.1004947 PMC4366209

[23] Tian, M. et al. Genomic regions associated with the sex-linked inhibitor of dermal melanin in Silkie chicken. *Front. Agr. Sci. Eng.* **1**, 242–249 (2014).

[24] Dharmayanthi, A. B. et al. The origin and evolution of fibromelanosis in domesticated chickens: genomic comparison of Indonesian Cemani and Chinese Silkie breeds. *PLoS ONE* **12**, e0173147 (2017). 28379963 10.1371/journal.pone.0173147 PMC5381777

[25] Sherman, R. M. et al. Assembly of a pan-genome from deep sequencing of 910 humans of African descent. *Nat. Genet.* **51**, 30–35 (2019). 30455414 10.1038/s41588-018-0273-y PMC6309586

[26] Tian, X. et al. Building a sequence map of the pig pan-genome from multiple de novo assemblies and Hi-C data. *Sci. China Life Sci.* **63**, 750–763 (2020). 31290097 10.1007/s11427-019-9551-7

[27] Wang, K. et al. The chicken pan-genome reveals gene content variation and a promoter region deletion in IGF2BP1 affecting body size. *Mol. Biol. Evol.* **38**, 5066–5081 (2021). 34329477 10.1093/molbev/msab231 PMC8557422

[28] Li, M. et al. De novo assembly of 20 chicken genomes reveals the undetectable phenomenon for thousands of core genes on micro-chromosomes and sub-telomeric regions. *Mol. Biol. Evol.* **39**, msac066 (2022). 10.1093/molbev/msac066 PMC9021737 35325213

[29] Dorshorst, B., Okimoto, R. & Ashwell, C. Genomic regions associated with dermal hyperpigmentation, polydactyly and other morphological traits in the Silkie chicken. *J. Hered.* **101**, 339–350 (2010). 20064842 10.1093/jhered/esp120

[30] Wong, G. K. et al. A genetic variation map for chicken with 2.8 million single-nucleotide polymorphisms. *Nature* **432**, 717–722 (2004). 15592405 10.1038/nature03156 PMC2263125

[31] O’Connor, R. E. et al. Patterns of microchromosome organization remain highly conserved throughout avian evolution. *Chromosoma* **128**, 21–29 (2019). 30448925 10.1007/s00412-018-0685-6 PMC6394684

[32] Solinhac, R. et al. Integrative mapping analysis of chicken microchromosome 16 organization. *BMC Genomics* **11**, 616 (2010). 21050458 10.1186/1471-2164-11-616 PMC3091757

[33] Feng, C. et al. A cis-regulatory mutation of PDSS2 causes silky-feather in chickens. *PLoS Genet.* **10**, e1004576 (2014). 25166907 10.1371/journal.pgen.1004576 PMC4148213

[34] Dunn, I. C. et al. The chicken polydactyly (Po) locus causes allelic imbalance and ectopic expression of Shh during limb development. *Dev. Dyn.* **240**, 1163–1172 (2011). 21465618 10.1002/dvdy.22623

[35] Imsland, F. et al. The Rose-comb mutation in chickens constitutes a structural rearrangement causing both altered comb morphology and defective sperm motility. *PLoS Genet.* **8**, e1002775 (2012). 22761584 10.1371/journal.pgen.1002775 PMC3386170

[36] Li, J. et al. The crest phenotype in domestic chicken is caused by a 197 bp duplication in the intron of HOXC10. *G3 (Bethesda)* **11**, jkaa048 (2021). 10.1093/g3journal/jkaa048 PMC8022956 33704432

[37] Dorshorst, B. et al. A complex genomic rearrangement involving the endothelin 3 locus causes dermal hyperpigmentation in the chicken. *PLoS Genet.* **7**, e1002412 (2011). 22216010 10.1371/journal.pgen.1002412 PMC3245302

[38] Silva, A. P. D. & Gallardo, R. A. The Chicken MHC: insights into genetic resistance, immunity, and inflammation following infectious bronchitis virus infections. *Vaccines* **8**, 637 (2020). 10.3390/vaccines8040637 PMC7711580 33147703

[39] Kaufman, J. et al. The chicken B locus is a minimal essential major histocompatibility complex. *Nature* **401**, 923–925 (1999). 10553909 10.1038/44856

[40] Wu, Y. et al. Structural definition of duck major histocompatibility complex class I molecules that might explain efficient cytotoxic T lymphocyte immunity to influenza A virus. *J. Virol.* **91**, 10–1128 (2017). 10.1128/JVI.02511-16 PMC5487541 28490583

[41] Moon, D. A., Veniamin, S. M., Parks-Dely, J. A. & Magor, K. E. The MHC of the duck (*Anas platyrhynchos*) contains five differentially expressed class I genes. *J. Immunol.* **175**, 6702–6712 (2005). 16272326 10.4049/jimmunol.175.10.6702

[42] Mesa, C. M., Thulien, K. J., Moon, D. A., Veniamin, S. M. & Magor, K. E. The dominant MHC class I gene is adjacent to the polymorphic TAP2 gene in the duck, Anas platyrhynchos. *Immunogenetics* **56**, 192–203 (2004). 15205935 10.1007/s00251-004-0672-3

[43] Walker, B. A., van Hateren, A., Milne, S., Beck, S. & Kaufman, J. Chicken TAP genes differ from their human orthologues in locus organisation, size, sequence features and polymorphism. *Immunogenetics* **57**, 232–247 (2005). 15900495 10.1007/s00251-005-0786-2

[44] Shaw, I. et al. Different evolutionary histories of the two classical class I genes BF1 and BF2 illustrate drift and selection within the stable MHC haplotypes of chickens. *J. Immunol.* **178**, 5744–5752 (2007). 17442958 10.4049/jimmunol.178.9.5744

[45] Shiina, T. et al. Comparative genomic analysis of two avian (quail and chicken) MHC regions. *J. Immunol.* **172**, 6751–6763 (2004). 15153492 10.4049/jimmunol.172.11.6751

[46] Chazara, O., Tixier-Boichard, M., Morin, V., Zoorob, R. & Bed’hom, B. Organisation and diversity of the class II DM region of the chicken MHC. *Mol. Immunol.* **48**, 1263–1271 (2011). 21481938 10.1016/j.molimm.2011.03.009

[47] Balakrishnan, C. N. et al. Gene duplication and fragmentation in the zebra finch major histocompatibility complex. *BMC Biol.* **8**, 29 (2010). 20359332 10.1186/1741-7007-8-29 PMC2907588

[48] Magor, K. E. et al. Defense genes missing from the flight division. *Dev. Comp. Immunol.* **41**, 377–388 (2013). 23624185 10.1016/j.dci.2013.04.010 PMC7172724

[49] Loehlin, D. W. & Carroll, S. B. Expression of tandem gene duplicates is often greater than twofold. *Proc. Natl. Acad. Sci. USA* **113**, 5988–5992 (2016). 27162370 10.1073/pnas.1605886113 PMC4889415

[50] Holland, P. W., Marletaz, F., Maeso, I., Dunwell, T. L. & Paps, J. New genes from old: asymmetric divergence of gene duplicates and the evolution of development. *Philos. Trans. R Soc. Lond. B Biol. Sci.* **372**, 20150480 (2017). 10.1098/rstb.2015.0480 PMC5182412 27994121

[51] Baudouin-Gonzalez, L. et al. Diverse cis-regulatory mechanisms contribute to expression evolution of tandem gene duplicates. *Mol. Biol. Evol.* **34**, 3132–3147 (2017). 28961967 10.1093/molbev/msx237 PMC5850857

[52] Chen, L., Fakiola, M., Staines, K., Butter, C. & Kaufman, J. Functional alleles of chicken BG genes, members of the Butyrophilin gene family, in peripheral T cells. *Front. Immunol.* **9**, 930 (2018). 29765375 10.3389/fimmu.2018.00930 PMC5938342

[53] Henry, J., Miller, M. M. & Pontarotti, P. Structure and evolution of the extended B7 family. *Immunol. Today* **20**, 285–288 (1999). 10354554 10.1016/s0167-5699(98)01418-2

[54] Dong, K., Chang, S., Xie, Q., Black-Pyrkosz, A. & Zhang, H. Comparative transcriptomics of genetically divergent lines of chickens in response to Marek’s disease virus challenge at cytolytic phase. *PLoS ONE* **12**, e0178923 (2017). 28591220 10.1371/journal.pone.0178923 PMC5462384

[55] You, Z. et al. Integrated analysis of lncRNA and mRNA repertoires in Marek’s disease infected spleens identifies genes relevant to resistance. *BMC Genomics* **20**, 245 (2019). 30922224 10.1186/s12864-019-5625-1 PMC6438004

[56] Sun, H., Liu, P., Nolan, L. K. & Lamont, S. J. Avian pathogenic *Escherichia coli* (APEC) infection alters bone marrow transcriptome in chickens. *BMC Genomics* **16**, 690 (2015). 26369556 10.1186/s12864-015-1850-4 PMC4570614

[57] Dakovic, N. et al. The loss of adipokine genes in the chicken genome and implications for insulin metabolism. *Mol. Biol. Evol.* **31**, 2637–2646 (2014). 25015647 10.1093/molbev/msu208

[58] Zhang, G. et al. Comparative genomics reveals insights into avian genome evolution and adaptation. *Science* **346**, 1311–1320 (2014). 25504712 10.1126/science.1251385 PMC4390078

[59] Yin, Z. T. et al. Revisiting avian ‘missing’ genes from de novo assembled transcripts. *BMC Genomics* **20**, 4 (2019). 30611188 10.1186/s12864-018-5407-1 PMC6321700

[60] Borst, S. E. The role of TNF-alpha in insulin resistance. *Endocrine* **23**, 177–182 (2004). 15146098 10.1385/ENDO:23:2-3:177

[61] Kalliolias, G. D. & Ivashkiv, L. B. TNF biology, pathogenic mechanisms and emerging therapeutic strategies. *Nat. Rev. Rheumatol.* **12**, 49–62 (2016). 26656660 10.1038/nrrheum.2015.169 PMC4809675

[62] Akash, M. S. H., Rehman, K. & Liaqat, A. Tumor necrosis factor-alpha: role in development of insulin resistance and pathogenesis of type 2 diabetes mellitus. *J. Cell Biochem.* **119**, 105–110 (2018). 28569437 10.1002/jcb.26174

[63] Kaiser, P. The long view: a bright past, a brighter future? Forty years of chicken immunology pre- and post-genome. *Avian Pathol.* **41**, 511–518 (2012). 23237363 10.1080/03079457.2012.735359

[64] Takimoto, T., Sato, K., Akiba, Y. & Takahashi, K. Role of chicken TL1A on inflammatory responses and partial characterization of its receptor. *J. Immunol.* **180**, 8327–8332 (2008). 18523299 10.4049/jimmunol.180.12.8327

[65] Bornelov, S. et al. Correspondence on Lovell et al.: Identification of chicken genes previously assumed to be evolutionarily lost. *Genome Biol.* **18**, 112 (2017). 28615067 10.1186/s13059-017-1231-1 PMC5470226

[66] Elleder, D. & Kaspers, B. After TNF-alpha, still playing hide-and-seek with chicken genes. *Poult. Sci.* **98**, 4373–4374 (2019). 31189184 10.3382/ps/pez307

[67] Qu, F. et al. Molecular identification and functional characterization of a tumor necrosis factor (TNF) gene in *Crassostrea hongkongensis*. *Immunobiology* **222**, 751–758 (2017). 28189340 10.1016/j.imbio.2017.02.002

[68] Reyes-Grajeda, J. P., Moreno, A. & Romero, A. Crystal structure of ovocleidin-17, a major protein of the calcified Gallus gallus eggshell: implications in the calcite mineral growth pattern. *J. Biol. Chem.* **279**, 40876–40881 (2004). 15263013 10.1074/jbc.M406033200

[69] Friedman, J. M. Leptin and the endocrine control of energy balance. *Nat. Metab.* **1**, 754–764 (2019). 32694767 10.1038/s42255-019-0095-y

[70] Pereira, S., Cline, D. L., Glavas, M. M., Covey, S. D. & Kieffer, T. J. Tissue-specific effects of leptin on glucose and lipid metabolism. *Endocr. Rev.* **42**, 1–28 (2021). 33150398 10.1210/endrev/bnaa027 PMC7846142

[71] Wang, P. et al. A leptin-BDNF pathway regulating sympathetic innervation of adipose tissue. *Nature* **583**, 839–844 (2020). 32699414 10.1038/s41586-020-2527-y

[72] Zhao, S. et al. Partial leptin reduction as an insulin sensitization and weight loss strategy. *Cell Metab.* **30**, 706–719.e706 (2019). 31495688 10.1016/j.cmet.2019.08.005 PMC6774814

[73] Taouis, M. et al. Cloning the chicken leptin gene. *Gene* **208**, 239–242 (1998). 9524275 10.1016/s0378-1119(97)00670-7

[74] Ashwell, C. M., Czerwinski, S. M., Brocht, D. M. & McMurtry, J. P. Hormonal regulation of leptin expression in broiler chickens. *Am. J. Physiol.* **276**, R226–R232 (1999). 9887199 10.1152/ajpregu.1999.276.1.R226

[75] Friedman-Einat, M. et al. The chicken leptin gene: has it been cloned? *Gen. Comp. Endocrinol.* **115**, 354–363 (1999). 10480986 10.1006/gcen.1999.7322

[76] Sharp, P. J., Dunn, I. C., Waddington, D. & Boswell, T. Chicken leptin. *Gen. Comp. Endocrinol.* **158**, 2–4 (2008). 18602102 10.1016/j.ygcen.2008.05.018

[77] Pitel, F., Faraut, T., Bruneau, G. & Monget, P. Is there a leptin gene in the chicken genome? Lessons from phylogenetics, bioinformatics and genomics. *Gen. Comp. Endocrinol.* **167**, 1–5 (2010). 19854194 10.1016/j.ygcen.2009.10.006

[78] Farkašová, H., Hron, T., Pačes, J., Pajer, P. & Elleder, D. Identification of a GC-rich leptin gene in chicken. *Agric. Gene* **1**, 88–92 (2016).

[79] Baek, M. et al. Accurate prediction of protein structures and interactions using a three-track neural network. *Science* **373**, 871–876 (2021). 34282049 10.1126/science.abj8754 PMC7612213

[80] Horev, G., Einat, P., Aharoni, T., Eshdat, Y. & Friedman-Einat, M. Molecular cloning and properties of the chicken leptin-receptor (CLEPR) gene. *Mol. Cell Endocrinol.* **162**, 95–106 (2000). 10854702 10.1016/s0303-7207(00)00205-7

[81] Tartaglia, L. A. et al. Identification and expression cloning of a leptin receptor, OB-R. *Cell* **83**, 1263–1271 (1995). 8548812 10.1016/0092-8674(95)90151-5

[82] Lee, G. H. et al. Abnormal splicing of the leptin receptor in diabetic mice. *Nature* **379**, 632–635 (1996). 8628397 10.1038/379632a0

[83] Pierce, B. G. et al. ZDOCK server: interactive docking prediction of protein-protein complexes and symmetric multimers. *Bioinformatics* **30**, 1771–1773 (2014). 24532726 10.1093/bioinformatics/btu097 PMC4058926

[84] Kim, J. et al. False gene and chromosome losses in genome assemblies caused by GC content variation and repeats. *Genome Biol.* **23**, 204 (2022). 36167554 10.1186/s13059-022-02765-0 PMC9516821

[85] Zhang, J. et al. Association of MHCY genotypes in lines of chickens divergently selected for high or low antibody response to sheep red blood cells. *Poult. Sci.* **101**, 101621 (2022). 34995879 10.1016/j.psj.2021.101621 PMC8741507

[86] Miller, M. M. & Taylor, R. L. Jr. Brief review of the chicken major histocompatibility complex: the genes, their distribution on chromosome 16, and their contributions to disease resistance. *Poult. Sci.* **95**, 375–392 (2016). 26740135 10.3382/ps/pev379 PMC4988538

[87] Zhang, J., Goto, R. M. & Miller, M. M. A simple means for MHC-Y genotyping in chickens using short tandem repeat sequences. *Immunogenetics* **72**, 325–332 (2020). 32488290 10.1007/s00251-020-01166-6

[88] Afanassieff, M. et al. At least one class I gene in restriction fragment pattern-Y (Rfp-Y), the second MHC gene cluster in the chicken, is transcribed, polymorphic, and shows divergent specialization in antigen binding region. *J. Immunol.* **166**, 3324–3333 (2001). 11207288 10.4049/jimmunol.166.5.3324

[89] Zhang, Y. et al. Positional cloning of the mouse obese gene and its human homologue. *Nature* **372**, 425–432 (1994). 7984236 10.1038/372425a0

[90] Prokop, J. W. et al. Discovery of the elusive leptin in birds: identification of several ‘missing links’ in the evolution of leptin and its receptor. *PLoS ONE* **9**, e92751 (2014). 24663438 10.1371/journal.pone.0092751 PMC3963946

[91] Consortium GT. Human genomics. The Genotype-Tissue Expression (GTEx) pilot analysis: multitissue gene regulation in humans. *Science* **348**, 648–660 (2015). 25954001 10.1126/science.1262110 PMC4547484

[92] Cheng, H., Concepcion, G. T., Feng, X., Zhang, H. & Li, H. Haplotype-resolved de novo assembly using phased assembly graphs with hifiasm. *Nat. Methods* **18**, 170–175 (2021). 33526886 10.1038/s41592-020-01056-5 PMC7961889

[93] Wang, J. R., Holt, J., McMillan, L. & Jones, C. D. FMLRC: Hybrid long read error correction using an FM-index. *BMC Bioinforma.* **19**, 50 (2018). 10.1186/s12859-018-2051-3 PMC5807796 29426289

[94] Hu, J., Fan, J., Sun, Z. & Liu, S. NextPolish: a fast and efficient genome polishing tool for long-read assembly. *Bioinformatics* **36**, 2253–2255 (2020). 31778144 10.1093/bioinformatics/btz891

[95] Chakraborty, M., Baldwin-Brown, J. G., Long, A. D. & Emerson, J. J. Contiguous and accurate de novo assembly of metazoan genomes with modest long read coverage. *Nucleic Acids Res.* **44**, e147 (2016). 27458204 10.1093/nar/gkw654 PMC5100563

[96] Durand, N. C. et al. Juicer provides a one-click system for analyzing loop-resolution Hi-C experiments. *Cell Syst.* **3**, 95–98 (2016). 27467249 10.1016/j.cels.2016.07.002 PMC5846465

[97] Ghurye, J. et al. Integrating Hi-C links with assembly graphs for chromosome-scale assembly. *PLoS Comput. Biol.* **15**, e1007273 (2019). 31433799 10.1371/journal.pcbi.1007273 PMC6719893

[98] Xu, M. et al. TGS-GapCloser: a fast and accurate gap closer for large genomes with low coverage of error-prone long reads. *GigaScience* **9**, giaa094 (2020). 10.1093/gigascience/giaa094 PMC7476103 32893860

[99] Seppey, M., Manni, M. & Zdobnov, E. M. BUSCO: assessing genome assembly and annotation completeness. *Methods Mol. Biol.* **1962**, 227–245 (2019). 31020564 10.1007/978-1-4939-9173-0_14

[100] Camacho, C. et al. BLAST+: architecture and applications. *BMC Bioinforma.* **10**, 421 (2009). 10.1186/1471-2105-10-421 PMC2803857 20003500

[101] Bolger, A. M., Lohse, M. & Usadel, B. Trimmomatic: a flexible trimmer for Illumina sequence data. *Bioinformatics* **30**, 2114–2120 (2014). 24695404 10.1093/bioinformatics/btu170 PMC4103590

[102] Grabherr, M. G. et al. Full-length transcriptome assembly from RNA-Seq data without a reference genome. *Nat. Biotechnol.* **29**, 644–652 (2011). 21572440 10.1038/nbt.1883 PMC3571712

[103] Kim, D., Paggi, J. M., Park, C., Bennett, C. & Salzberg, S. L. Graph-based genome alignment and genotyping with HISAT2 and HISAT-genotype. *Nat. Biotechnol.* **37**, 907–915 (2019). 31375807 10.1038/s41587-019-0201-4 PMC7605509

[104] Anders, S., Pyl, P. T. & Huber, W. HTSeq–a Python framework to work with high-throughput sequencing data. *Bioinformatics* **31**, 166–169 (2015). 25260700 10.1093/bioinformatics/btu638 PMC4287950

[105] Love, M. I., Huber, W. & Anders, S. Moderated estimation of fold change and dispersion for RNA-seq data with DESeq2. *Genome Biol.* **15**, 550 (2014). 25516281 10.1186/s13059-014-0550-8 PMC4302049

[106] Sherman, B. T. et al. DAVID: a web server for functional enrichment analysis and functional annotation of gene lists (2021 update). *Nucleic Acids Res.* **50**, W216–W221 (2022). 35325185 10.1093/nar/gkac194 PMC9252805

[107] Haas, B. J. et al. Automated eukaryotic gene structure annotation using EVidenceModeler and the Program to Assemble Spliced Alignments. *Genome Biol.* **9**, R7 (2008). 18190707 10.1186/gb-2008-9-1-r7 PMC2395244

[108] Bruna, T., Hoff, K. J., Lomsadze, A., Stanke, M. & Borodovsky, M. BRAKER2: automatic eukaryotic genome annotation with GeneMark-EP+ and AUGUSTUS supported by a protein database. *NAR Genom. Bioinforma.* **3**, lqaa108 (2021). 10.1093/nargab/lqaa108 PMC7787252 33575650

[109] Stanke, M. & Morgenstern, B. AUGUSTUS: a web server for gene prediction in eukaryotes that allows user-defined constraints. *Nucleic Acids Res.* **33**, W465–W467 (2005). 15980513 10.1093/nar/gki458 PMC1160219

[110] Haas, B. J. et al. De novo transcript sequence reconstruction from RNA-seq using the Trinity platform for reference generation and analysis. *Nat. Protoc.* **8**, 1494–1512 (2013). 23845962 10.1038/nprot.2013.084 PMC3875132

[111] Chan, P. P. & Lowe, T. M. tRNAscan-SE: searching for tRNA genes in genomic sequences. *Methods Mol. Biol.* **1962**, 1–14 (2019). 31020551 10.1007/978-1-4939-9173-0_1 PMC6768409

[112] Kalvari, I. et al. Rfam 13.0: shifting to a genome-centric resource for non-coding RNA families. *Nucleic Acids Res.* **46**, D335–D342 (2018). 29112718 10.1093/nar/gkx1038 PMC5753348

[113] Nawrocki, E. P., Kolbe, D. L. & Eddy, S. R. Infernal 1.0: inference of RNA alignments. *Bioinformatics* **25**, 1335–1337 (2009). 19307242 10.1093/bioinformatics/btp157 PMC2732312

[114] Flynn, J. M. et al. RepeatModeler2 for automated genomic discovery of transposable element families. *Proc. Natl. Acad. Sci. USA* **117**, 9451–9457 (2020). 32300014 10.1073/pnas.1921046117 PMC7196820

[115] Tarailo-Graovac, M. & Chen, N. Using RepeatMasker to identify repetitive elements in genomic sequences. *Curr. Protoc. Bioinforma.* Chapter 4, Unit 4 10 (2009). 10.1002/0471250953.bi0410s25 19274634

[116] Jain, C., Rhie, A., Hansen, N. F., Koren, S. & Phillippy, A. M. Long-read mapping to repetitive reference sequences using Winnowmap2. *Nat. Methods* **19**, 705–710 (2022). 35365778 10.1038/s41592-022-01457-8 PMC10510034

[117] Heller, D. & Vingron, M. SVIM-asm: Structural variant detection from haploid and diploid genome assemblies. *Bioinformatics* **36**, 5519–5521 (2020). 10.1093/bioinformatics/btaa1034 PMC8016491 33346817

[118] Jeffares, D. C. et al. Transient structural variations have strong effects on quantitative traits and reproductive isolation in fission yeast. *Nat. Commun.* **8**, 14061 (2017). 28117401 10.1038/ncomms14061 PMC5286201

[119] Marcais, G. et al. MUMmer4: a fast and versatile genome alignment system. *PLoS Comput. Biol.* **14**, e1005944 (2018). 29373581 10.1371/journal.pcbi.1005944 PMC5802927

[120] Wang, Y. et al. MCScanX: a toolkit for detection and evolutionary analysis of gene synteny and collinearity. *Nucleic Acids Res.* **40**, e49 (2012). 22217600 10.1093/nar/gkr1293 PMC3326336

[121] Chiang, C. et al. SpeedSeq: ultra-fast personal genome analysis and interpretation. *Nat. Methods* **12**, 966–968 (2015). 26258291 10.1038/nmeth.3505 PMC4589466

[122] Li, H. & Durbin, R. Fast and accurate short read alignment with Burrows-Wheeler transform. *Bioinformatics* **25**, 1754–1760 (2009). 19451168 10.1093/bioinformatics/btp324 PMC2705234

[123] Li, H. et al. The Sequence Alignment/Map format and SAMtools. *Bioinformatics* **25**, 2078–2079 (2009). 19505943 10.1093/bioinformatics/btp352 PMC2723002

[124] Goel, M., Sun, H., Jiao, W. B. & Schneeberger, K. SyRI: finding genomic rearrangements and local sequence differences from whole-genome assemblies. *Genome Biol.* **20**, 277 (2019). 31842948 10.1186/s13059-019-1911-0 PMC6913012

[125] Kolmogorov, M., Yuan, J., Lin, Y. & Pevzner, P. A. Assembly of long, error-prone reads using repeat graphs. *Nat. Biotechnol.* **37**, 540–546 (2019). 30936562 10.1038/s41587-019-0072-8

[126] Johnson, M. et al. NCBI BLAST: a better web interface. *Nucleic Acids Res.* **36**, W5–W9 (2008). 18440982 10.1093/nar/gkn201 PMC2447716

[127] Delcher, A. L., Salzberg, S. L. & Phillippy, A. M. Using MUMmer to identify similar regions in large sequence sets. *Curr. Protoc. Bioinforma.* **Chapter 10**, Unit 10 13 (2003). 10.1002/0471250953.bi1003s00 18428693

[128] Gouet, P., Robert, X. & Courcelle, E. ESPript/ENDscript: extracting and rendering sequence and 3D information from atomic structures of proteins. *Nucleic Acids Res.* **31**, 3320–3323 (2003). 12824317 10.1093/nar/gkg556 PMC168963

[129] Yuen, Z. W. et al. Systematic benchmarking of tools for CpG methylation detection from nanopore sequencing. *Nat. Commun.* **12**, 3438 (2021). 34103501 10.1038/s41467-021-23778-6 PMC8187371

[130] Botero-Castro, F., Figuet, E., Tilak, M. K., Nabholz, B. & Galtier, N. Avian genomes revisited: hidden genes uncovered and the rates versus traits paradox in birds. *Mol. Biol. Evol.* **34**, 3123–3131 (2017). 28962031 10.1093/molbev/msx236

[131] Firtina, C. et al. Apollo: a sequencing-technology-independent, scalable and accurate assembly polishing algorithm. *Bioinformatics* **36**, 3669–3679 (2020). 32167530 10.1093/bioinformatics/btaa179

[132] Bailey, T. L., Johnson, J., Grant, C. E. & Noble, W. S. The MEME Suite. *Nucleic Acids Res.* **43**, W39–W49 (2015). 25953851 10.1093/nar/gkv416 PMC4489269

[133] Zhu, F. et al. Johnsonzcode/CAU_Silkie_code: for publishing (v1.0.0). *Zenodo*10.5281/zenodo.10077021 (2023).

