# Supplementary material for: A chromosome-level genome assembly for the Silkie chicken resolves complete sequences for key chicken metabolic, reproductive, and immunity genes
Source: Commun Biol. 2023 Dec 6;6:1233. doi: 10.1038/s42003-023-05619-y (PMC10700341; doi:10.1038/s42003-023-05619-y)
Supplement: Supplementary file 1 — Supplementary Information [file 42003_2023_5619_MOESM1_ESM.pdf]

Supplementary Information for

Zhu, et al. A chromosome-level genome assembly for the Silkie chicken resolves complete sequences for key chicken metabolic, reproductive, and immunity genes

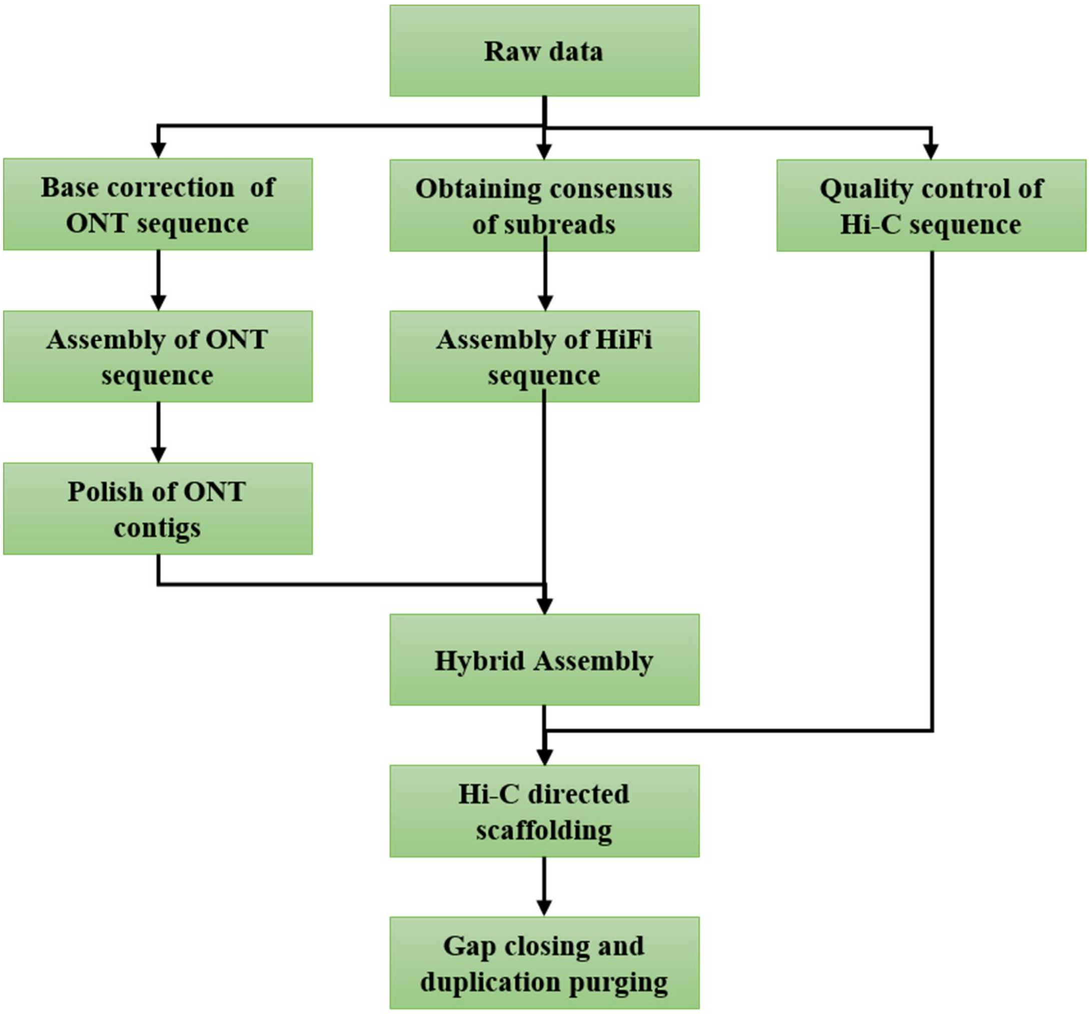

Supplementary Figure 1. The pipeline for genome assembly. Three technology have been adopted here: PacBio HiFi reads, ONT long reads sequencing and Hi-C short reads sequencing. First, HiFi reads were generated from obtaining consensus of subreads; second, base correction for ONT long reads was processed with HiFi long reads with depth > 39; third, a high quality and continuity hybrid assembly was combined from ONT assembly and HiFi assembly; forth, Hi-C directed scaffolding was carried out; finally, the gap in assembly was closed and haplotype duplication was purged.

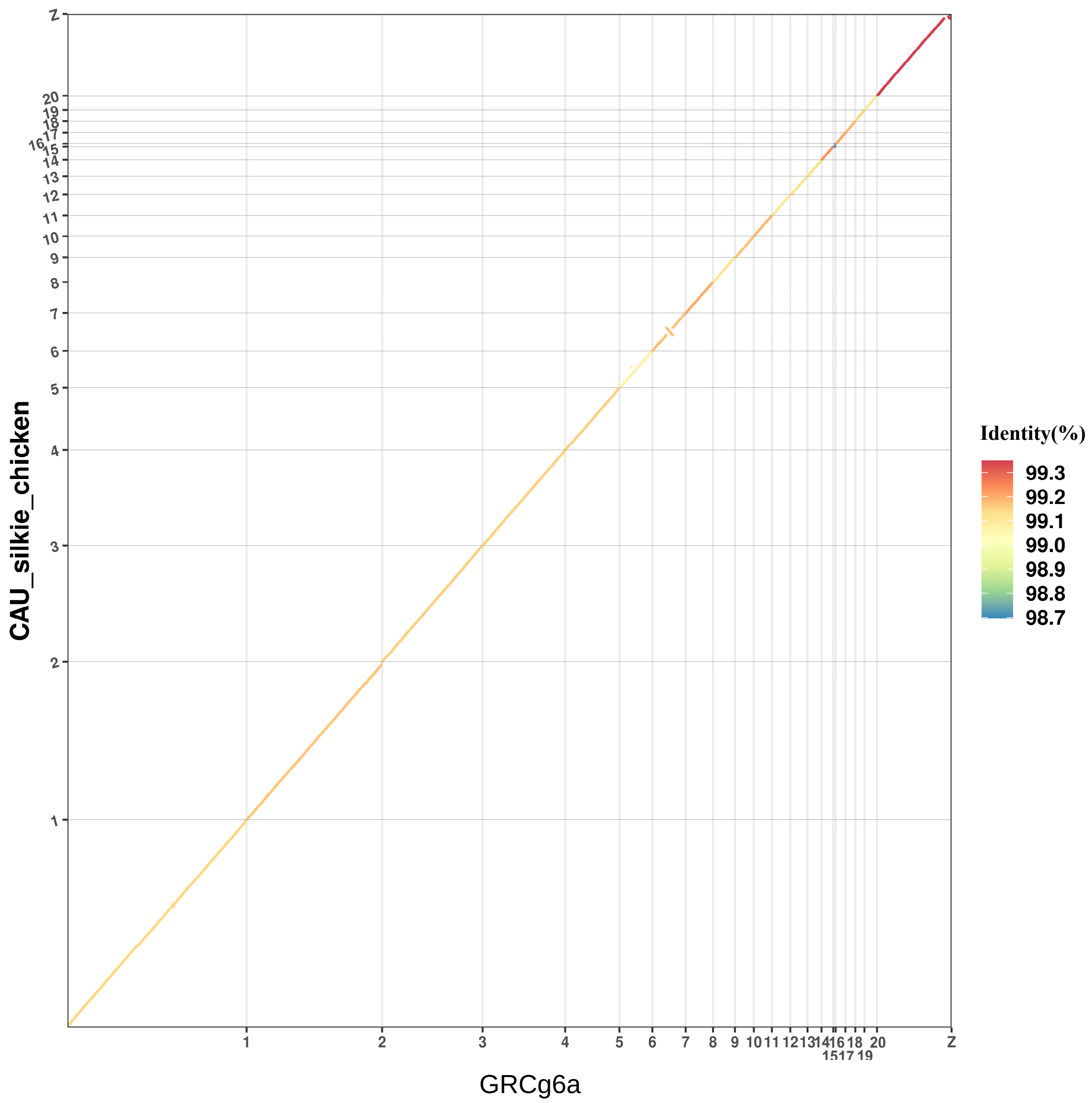

Supplementary Figure 2. Collinear dot-plots between CAU_Silkie and GRCg6a. Genome collinearity between GRCg6a and CAU_Silkie for their chromosome was constructed from NUCMER. Diagonal line represents a good collinearity. The legend indicates the sequence identity between pair of chromosomes. Light blue show lower similarity between pair of chromosomes. And red show higher similarity between pair of chromosomes. Short line in chromosome 7 indicates an inversion.

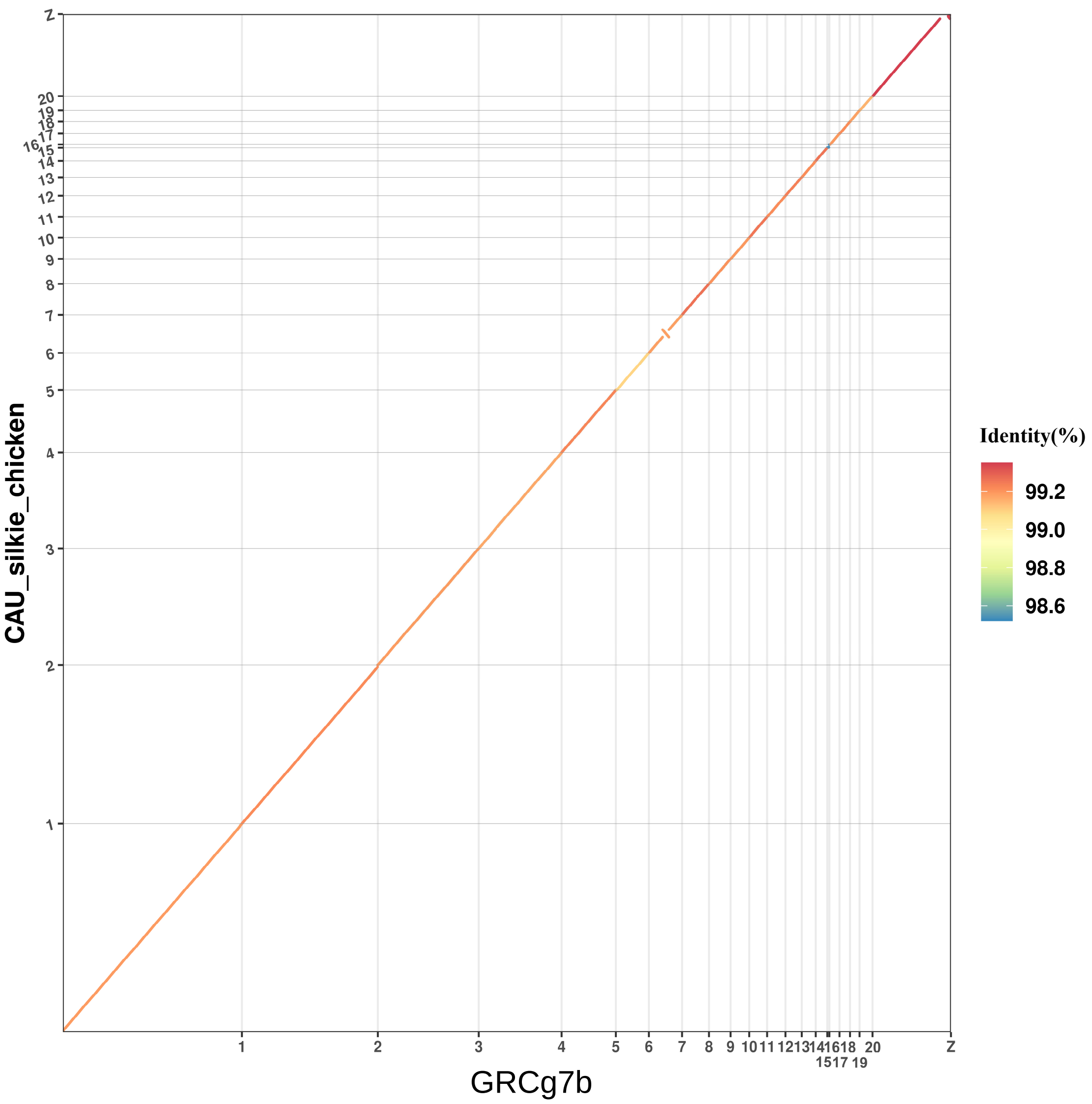

Supplementary Figure 3. Collinear dot-plots between CAU_Silkie and GRCg7b. Genome collinearity between GRCg7b and CAU_Silkie for their chromosome was constructed from NUCMER. Diagonal line represents a good collinearity. The legend indicates the sequence identity between pair of chromosomes. Light blue show lower similarity between pair of chromosomes. And red show higher similarity between pair of chromosomes. Short line in chromosome 7 indicates an inversion.

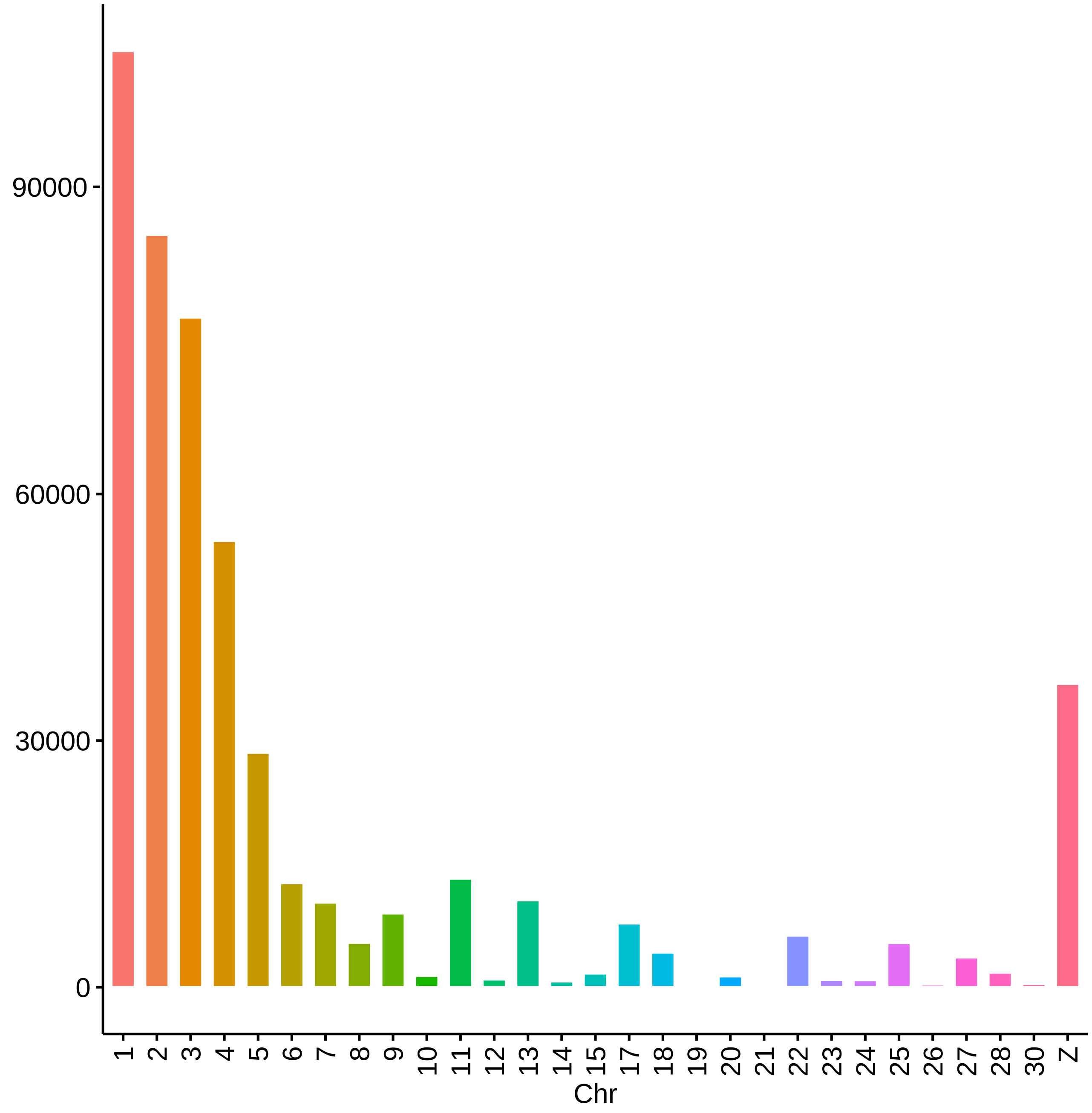

Supplementary Figure 4. Chromosome distribution of total length of shared DEL for 7 other chicken assemblies on chr1-30 and chrZ. The horizontal coordinate is the chromosome and the vertical coordinate is the sum of shared DEL for 7 other chicken compared to CAU_Silkie.
(Huxu: GCA_024206055.1, White_Leghorn: GCA_024652995.1, Silkies: GCA_024653025.1, Rhode_Island_Red: GCA_024652985.1, Houdan: GCA_024653045.1 Cornish: GCA_024653035.1 and GRCg7b)

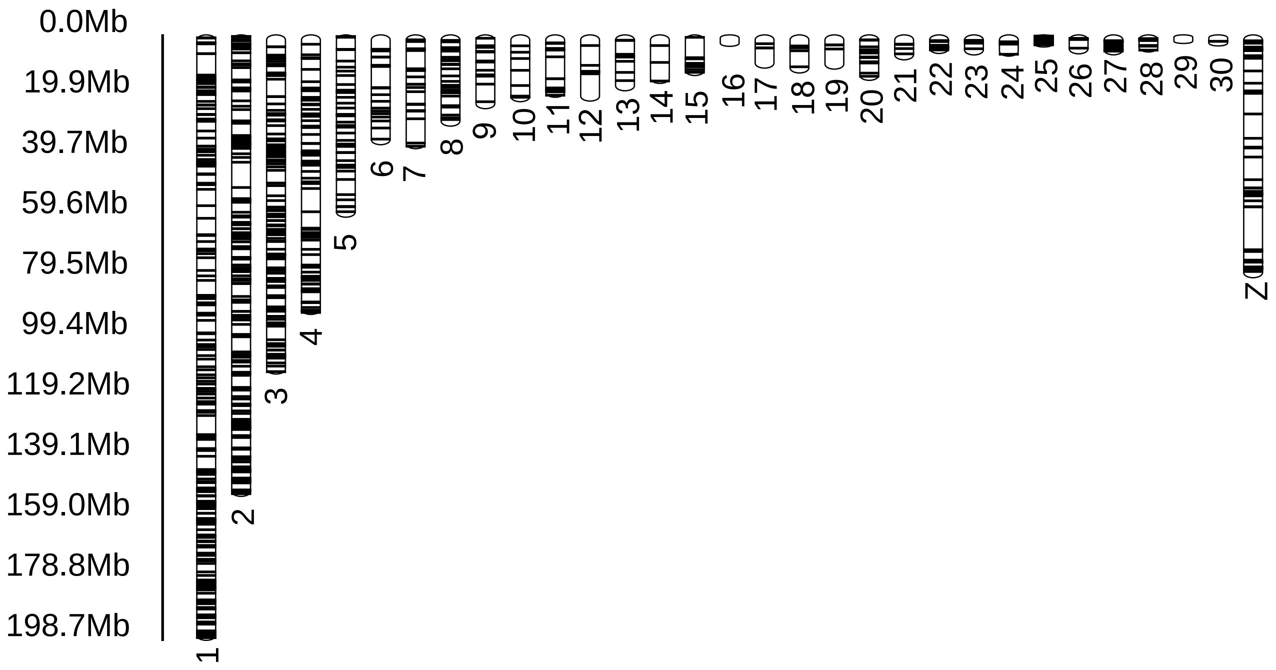

Supplementary Figure 5. Distribution of CAU_Silkie newly assembled sequences in chr1-30 and chrZ. The dark color of chromosome represents shared total length of DEL in 7 other chicken compared to CAU_Silkie.
(Huxu: GCA_024206055.1, White_Leghorn: GCA_024652995.1, Silkies: GCA_024653025.1, Rhode_Island_Red: GCA_024652985.1, Houdan: GCA_024653045.1 Cornish: GCA_024653035.1 and GRCg7b)

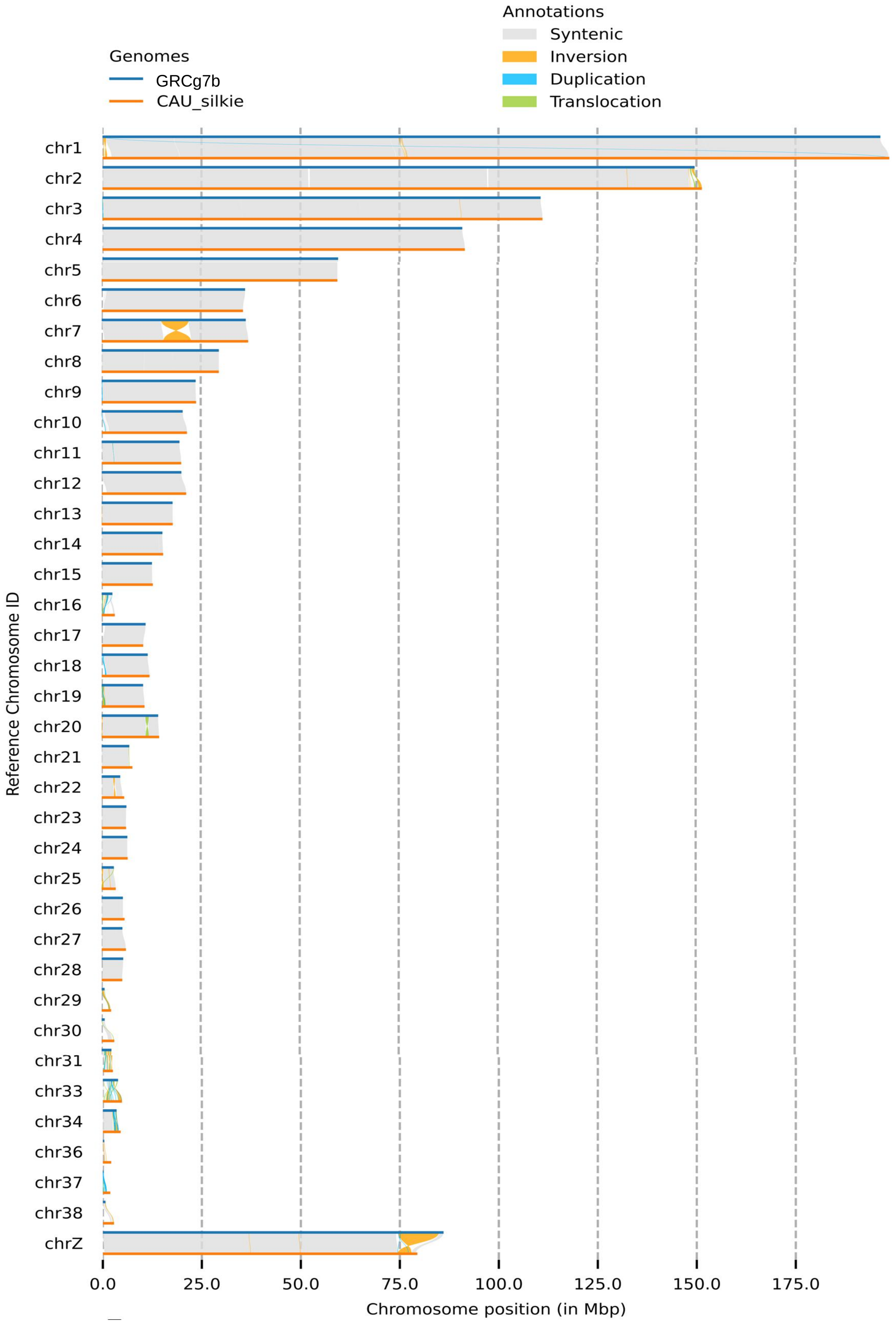

Supplementary Figure 6. Comparative genomes reveal SVs between GRCg7b and CAU_Silkie.

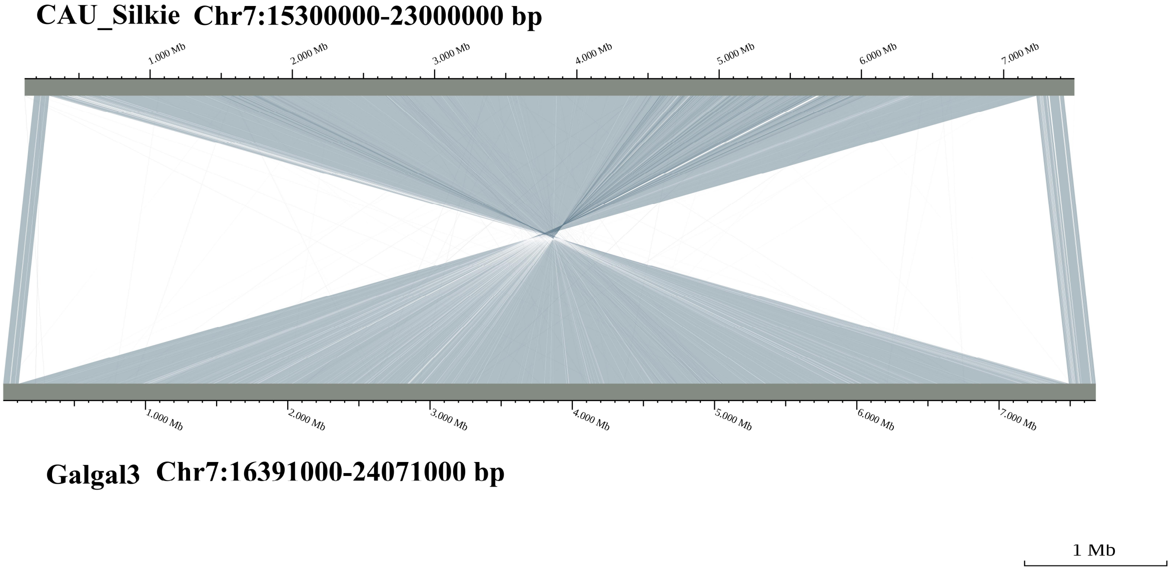

Supplementary Figure 7. Identification of rose comb variation. The allele corresponding to the rose comb corresponds to the mutant chromosome structure (Imsland F, Feng C, Boije H, et al. The Rose-comb mutation in chickens constitutes a structural rearrangement causing both altered comb morphology and defective sperm motility. PLoS Genet. 2012;8(6):e1002775. doi:10.1371/journal.pgen.1002775.)

```
chr3_70486523-70486723    1  TCAGAGCGCTCTGCGACTCTCAACGCGGGAACGCCGCGAG
chr3_67930722-67930923    1  TCAGAGCGCTCTGCGACTCTCAACGCGGGAACGCCGCGAG

chr3_70486523-70486723   41  AGGCCGTCAGGCGCGGAAGACGAGCGAAGCGGGAAGGGAG
chr3_67930722-67930923   41  AGGCCGTCAGGCGCGGAAGACGAGCGAAGCGGGAAGGGAG

chr3_70486523-70486723   81  AGCCGCGCTGCCTCGCTTTACGGCCCGTGTGCGACGCGCA
chr3_67930722-67930923   81  AGCCGCGCTGCCTCGCTTTAGGGCCCGTGTGCGACGCGCA

chr3_70486523-70486723  121  AGATGGCTGCCCCCAGGGCGCAATAAGGCCGCGCCGGTCC
chr3_67930722-67930923  121  AGATGGCTGCCCCCAGGGCGCAATAAGGCCGCGCCGGTCC

chr3_70486523-70486723  161  GCCGCCCGGGG.CCGAGCCGGGCCGAGCAGCGGCGCGGGG
chr3_67930722-67930923  161  GCCGCCCGGGGGCCGAGCCGGGCCGAGCAGCGGCGCGGGG

chr3_70486523-70486723  200  CG
chr3_67930722-67930923  201  CG
```

Supplementary Figure 8. Identification of silky-feather variation. It is reported that silky-feather is determined by the SNP C > G of the 103 bp upstream of PDSS2 (Galgal3). By extracting the corresponding region of the genome of Galgal3(Chr3:70486523-70486723) and comparing it to CAU_Silkie(Chr3:67930722-67930923), the corresponding SNP was obtained at the 100th bp, which is SNP C > G (Feng C, Gao Y, Dorshorst B, Song C, Gu X, Li Q, et al. A cis-regulatory mutation of PDSS2 causes silky-feather in chickens. PLoS Genet. 2014;10(8):e1004576. Robert, X. and Gouet, P. (2014) "Deciphering key features in protein structures with the new ENDscript server". Nucl. Acids Res. 42(W1), W320-W324 - doi: 10.1093/nar/gku316).

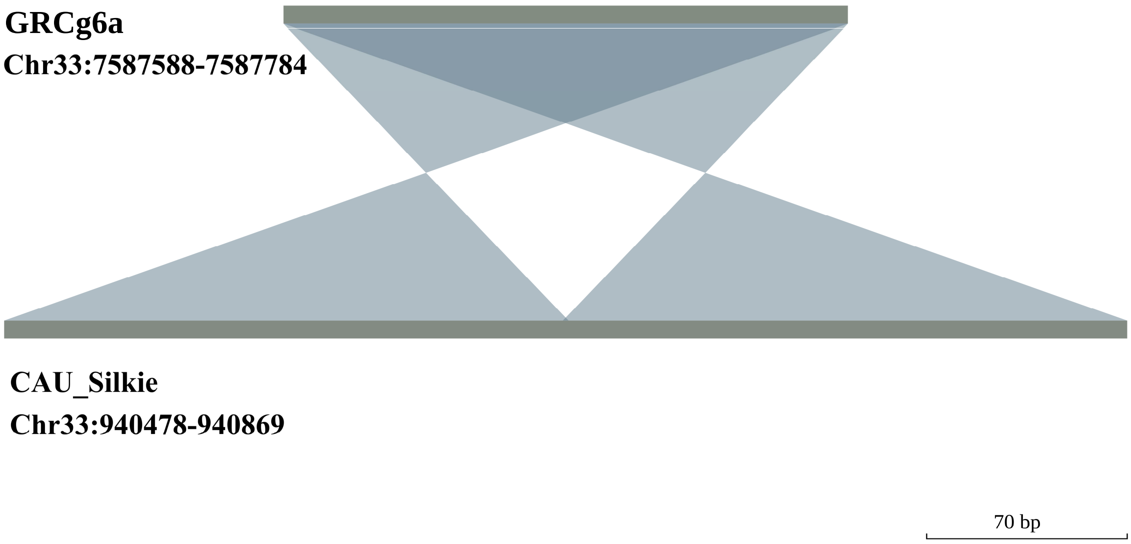

Supplementary Figure 9. Identification of crest variation. It is repoted that the crest is caused by a 197 bp tandem repeat on the chromosome 33. There is a 197 bp tandem repeats in CAU_Silkie Chr33(Li J, Lee MO, Davis BW, Wu P, Hsieh Li SM, Chuong CM, et al. The crest phenotype in domestic chicken is caused by a 197 bp duplication in the intron of HOXC10. G3 (Bethesda). 2021;11(2)).

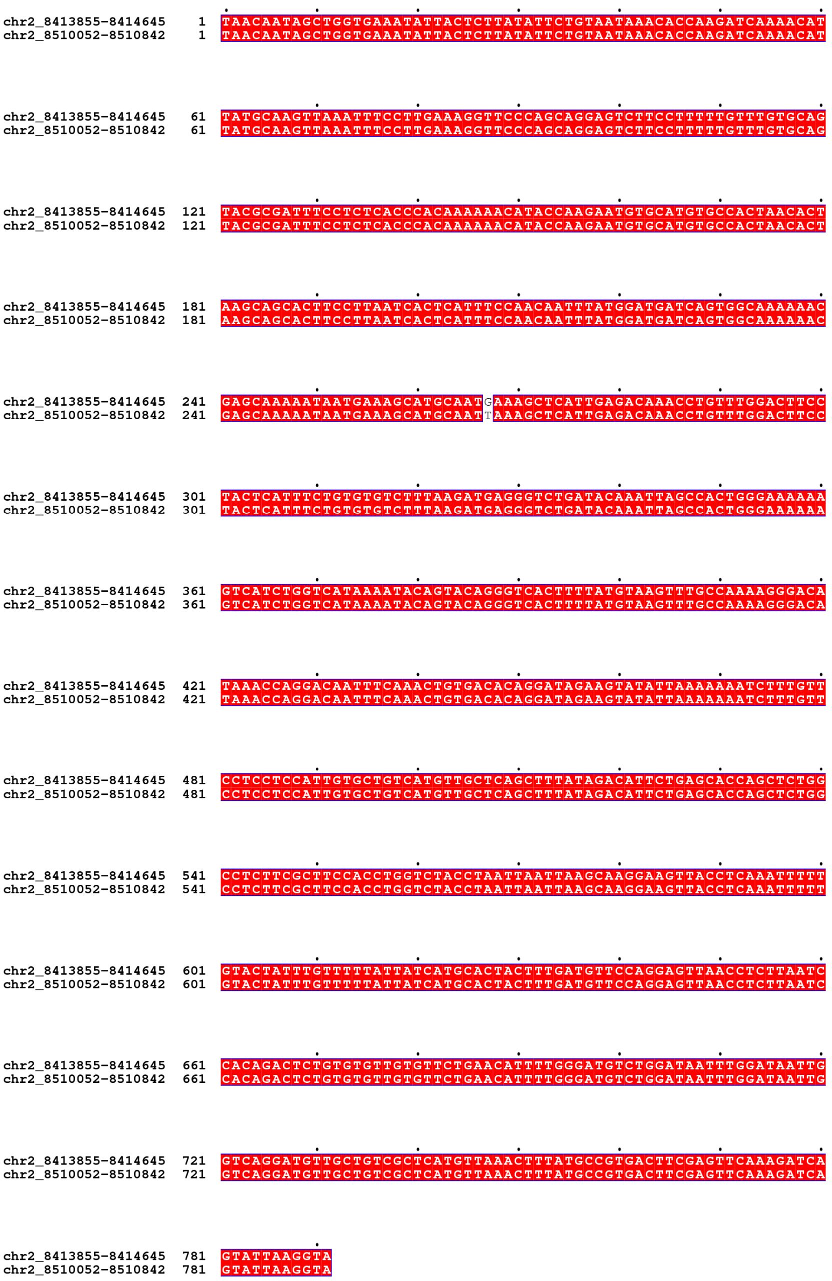

Supplementary Figure 10. Identification of Polydactyly variation. It is reported that polydactyly is caused by SNP C > A in intron ZRS region of LMBR1 gene(Dunn IC, Paton IR, Clelland AK, Sebastian S, Johnson EJ, McTeir L, et al. The chicken polydactyly (Po) locus causes allelic imbalance and ectopic expression of Shh during limb development. Dev Dyn. 2011;240(5):1163-72).

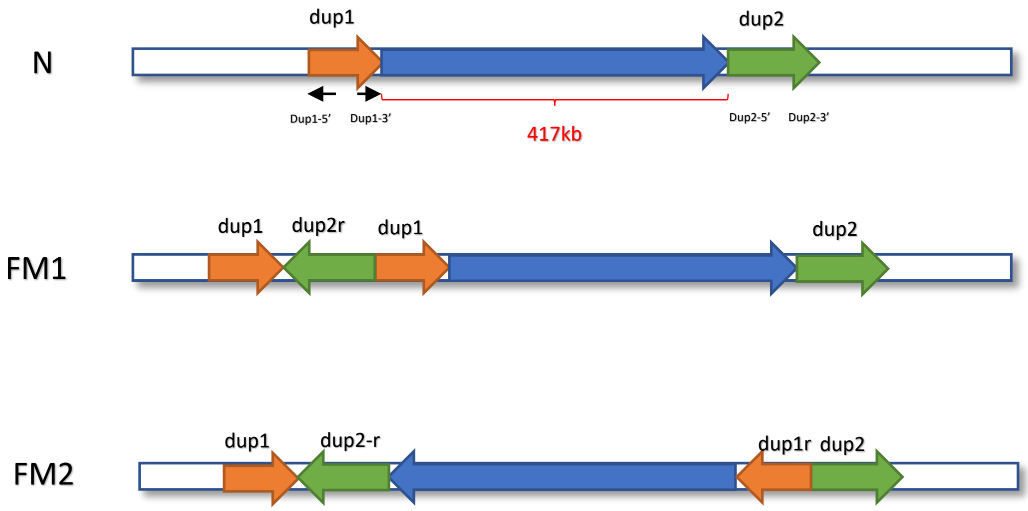

Supplementary Figure 11. The possible rearrangement hypothesis of hyperpigmentation. N represents wild-type, FM1 represents rearrangement hypothesis from Dorshorst et al(2011). FM2 represents assembly in this study.

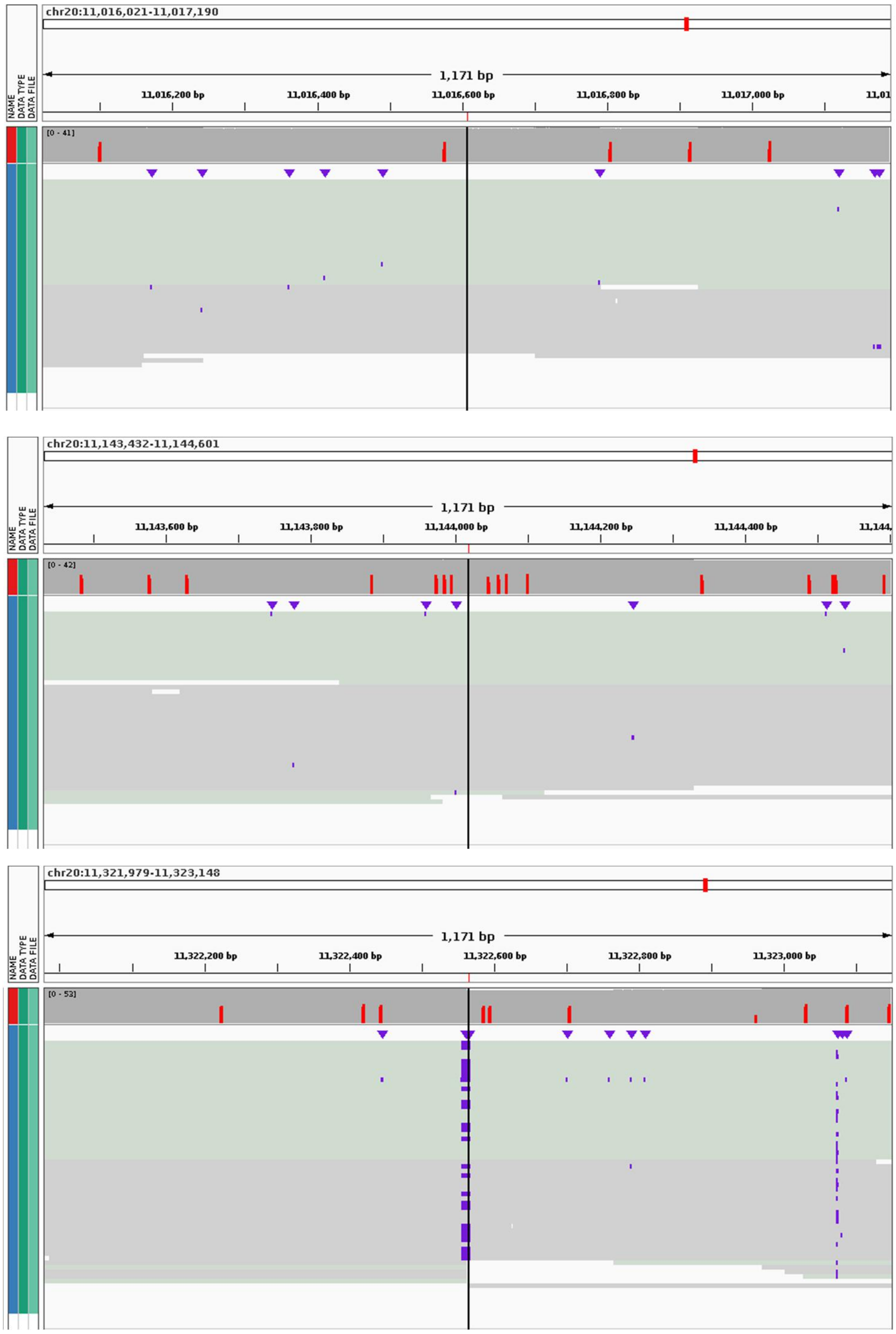

Supplementary Figure 12. IGV view of HiFi reads at the position of chr20. dup1-5'(11016607), dup1-3'(11144018) and dup2r-5'(11322565) respectively(Fig.1).

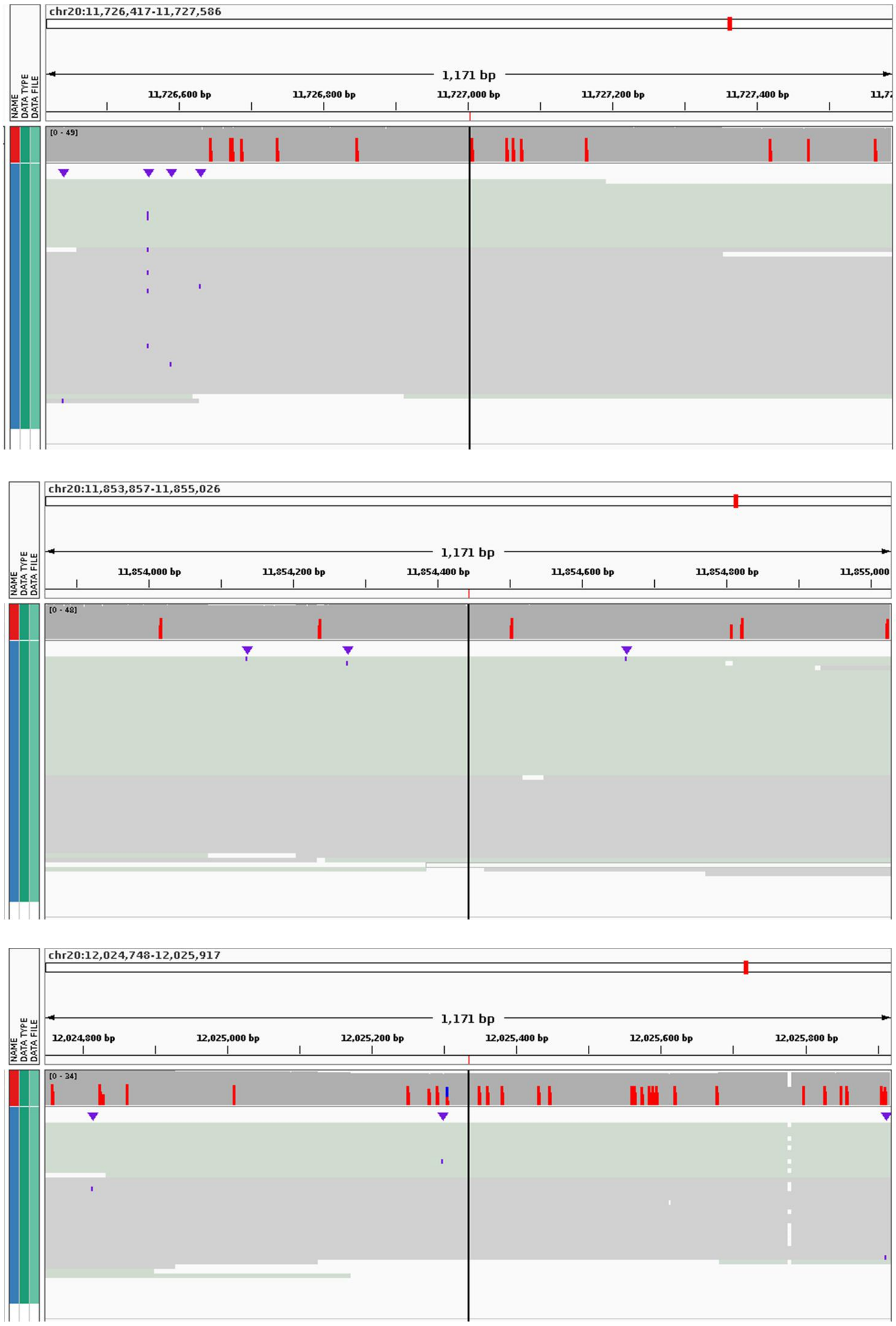

Supplementary Figure 13. IGV view of HiFi reads at the position of chr20. dup2r-3’(11727003), dup2-5’(11854443) and dup2-3’(12025334) respectively(Fig.1).

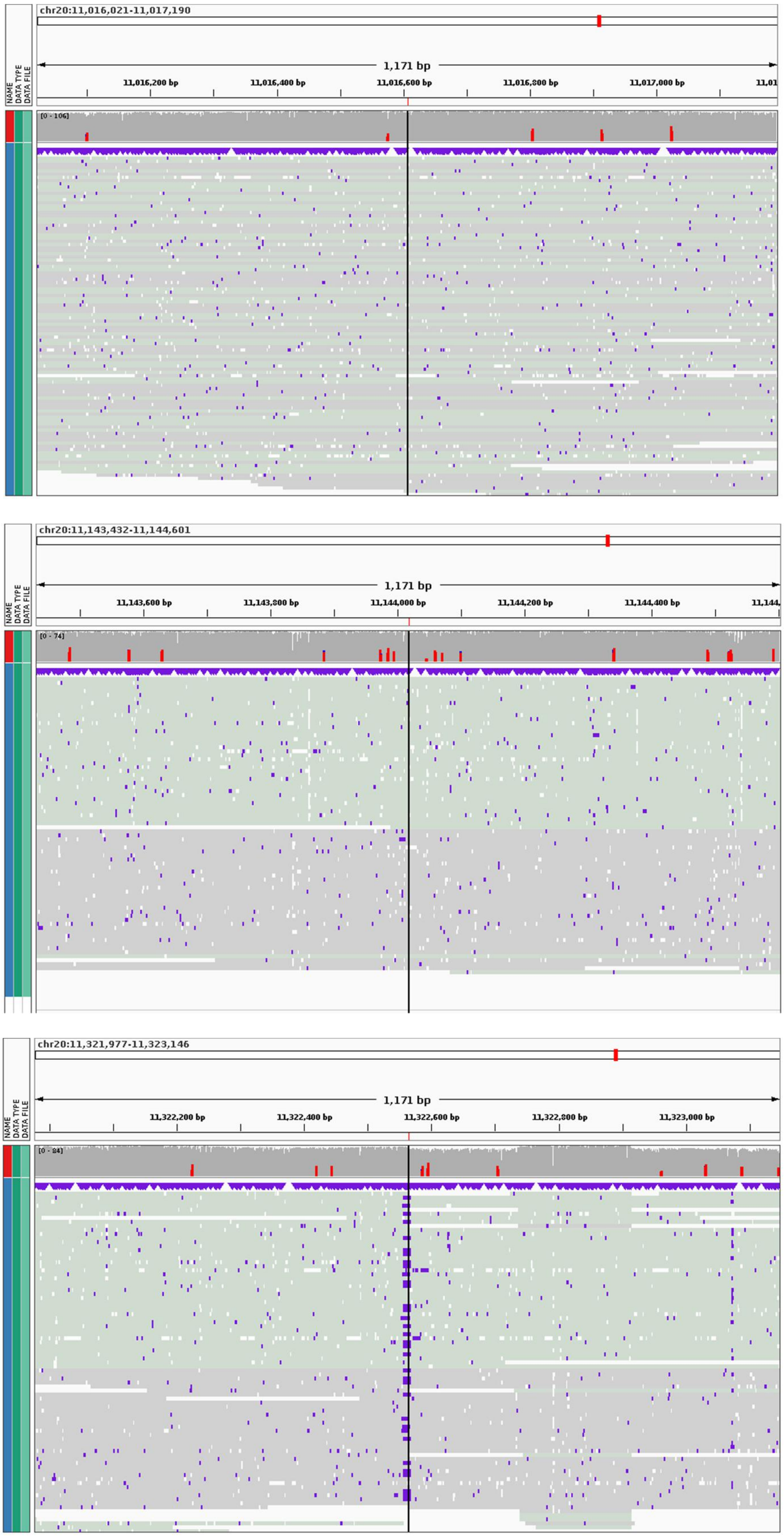

Supplementary Figure 14. IGV view of ONT reads at the position of chr20. dup1-5'(11016607), dup1-3'(11144018) and dup2r-5'(11322565) respectively(Fig.1).

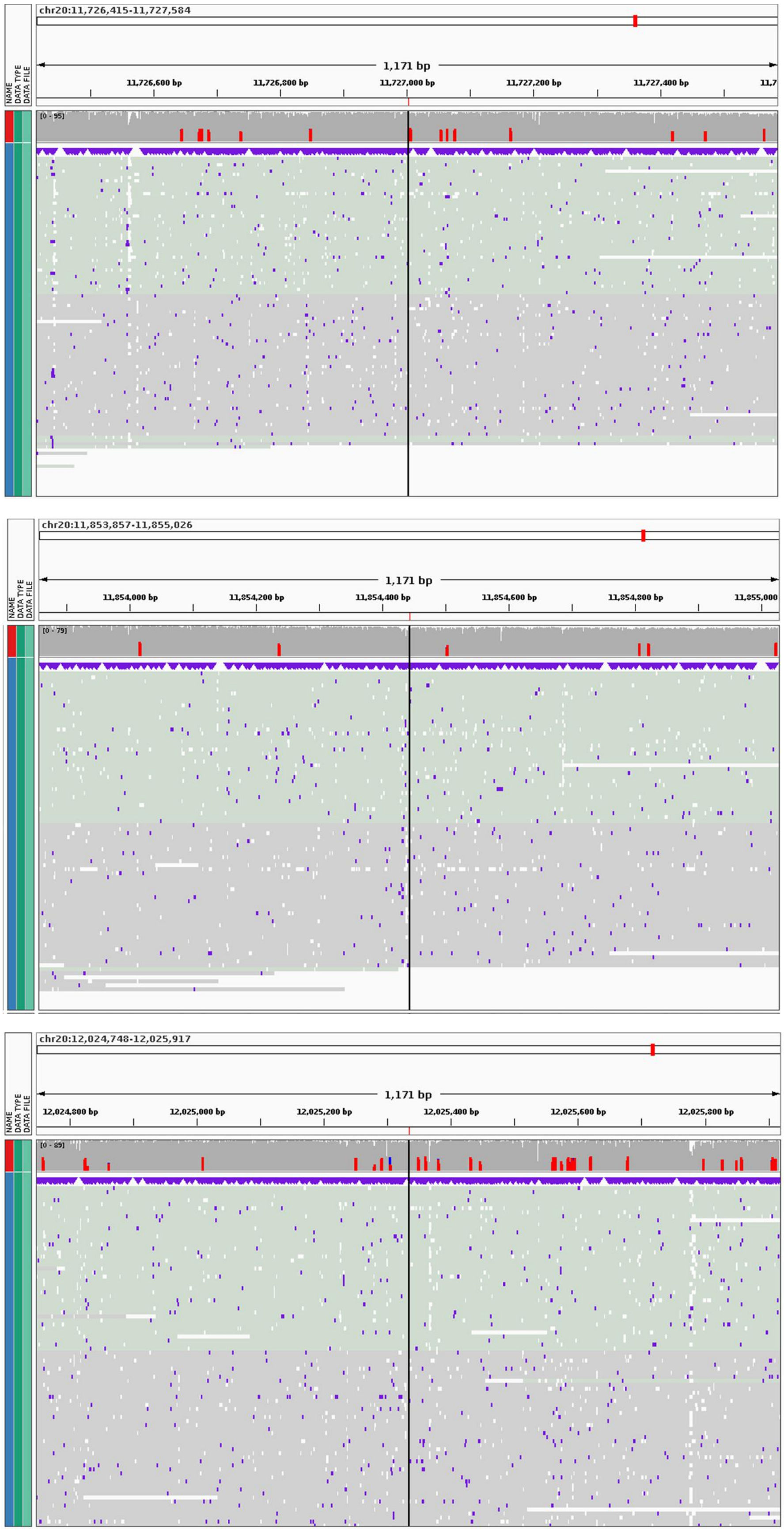

Supplementary Figure 15. IGV view of ONT reads at the position of chr20. dup2r-3'(11727003), dup2-5'(11854443) and dup2-3'(12025334) respectively(Fig.1).

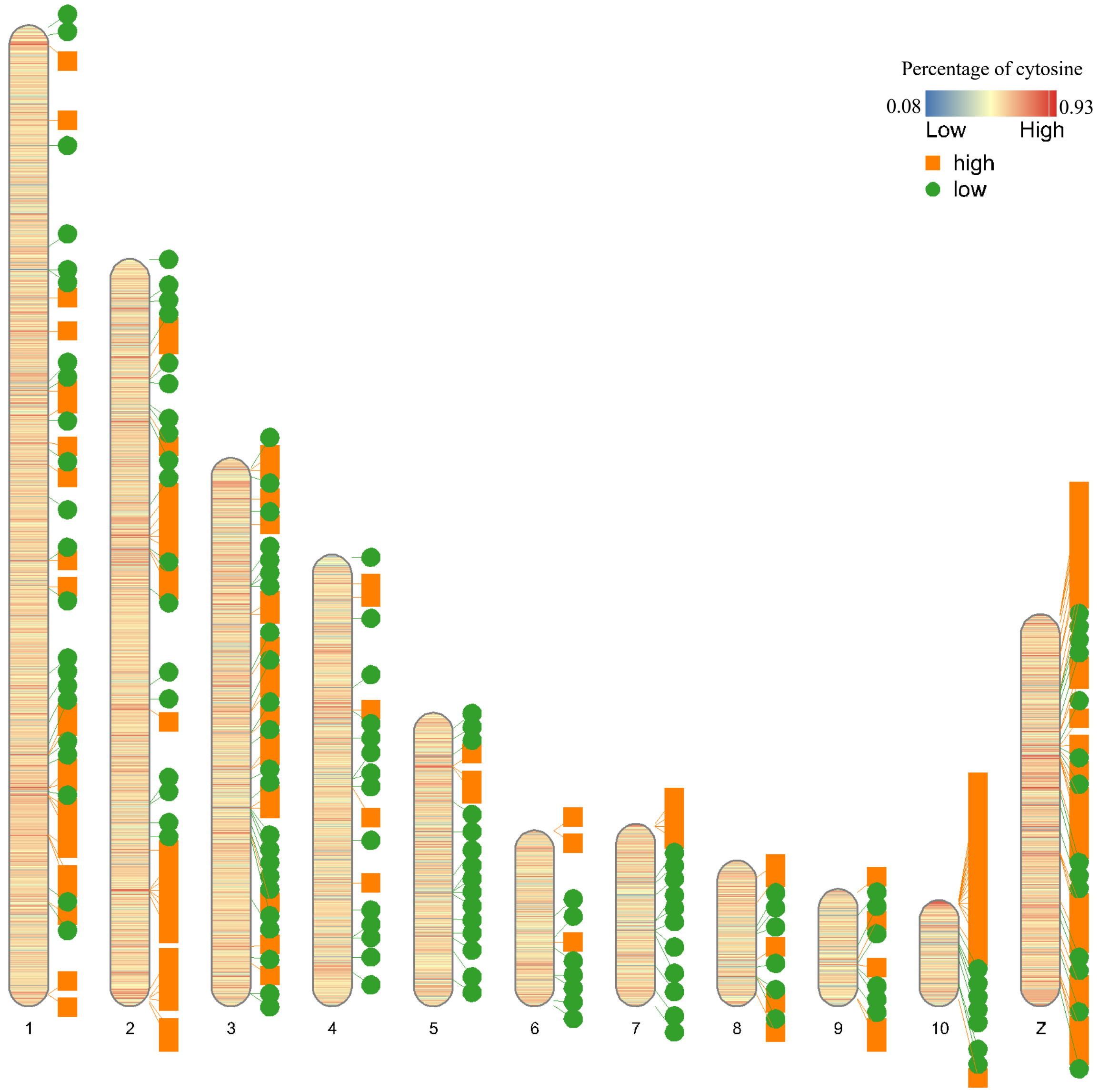

Supplementary Figure 16. Methylation landscape of CAU_Silkie chr1-10 and chrZ. The bands of chromosomes represent the high and low methylation frequencies within the 50kbp window, with orange squares representing the region as a hypermethylation region and green circles representing the region as a hypomethylated region**.**

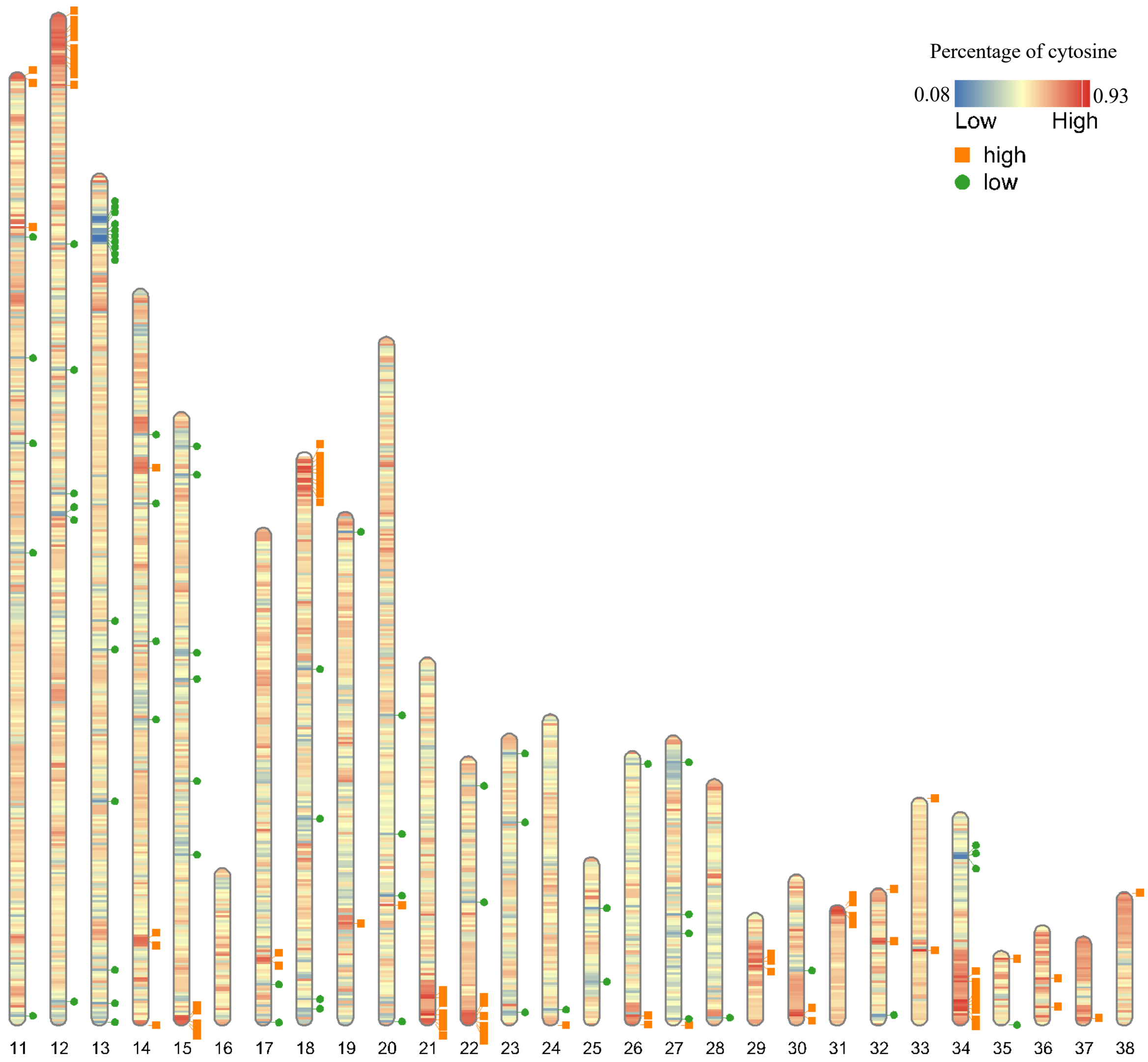

Supplementary Figure 17. Methylation landscape of CAU_Silkie chr11-38. The bands of chromosomes represent the high and low methylation frequencies within the 50kbp window, with orange squares representing the region as a hypermethylation region and green circles representing the region as a hypomethylated region**.**

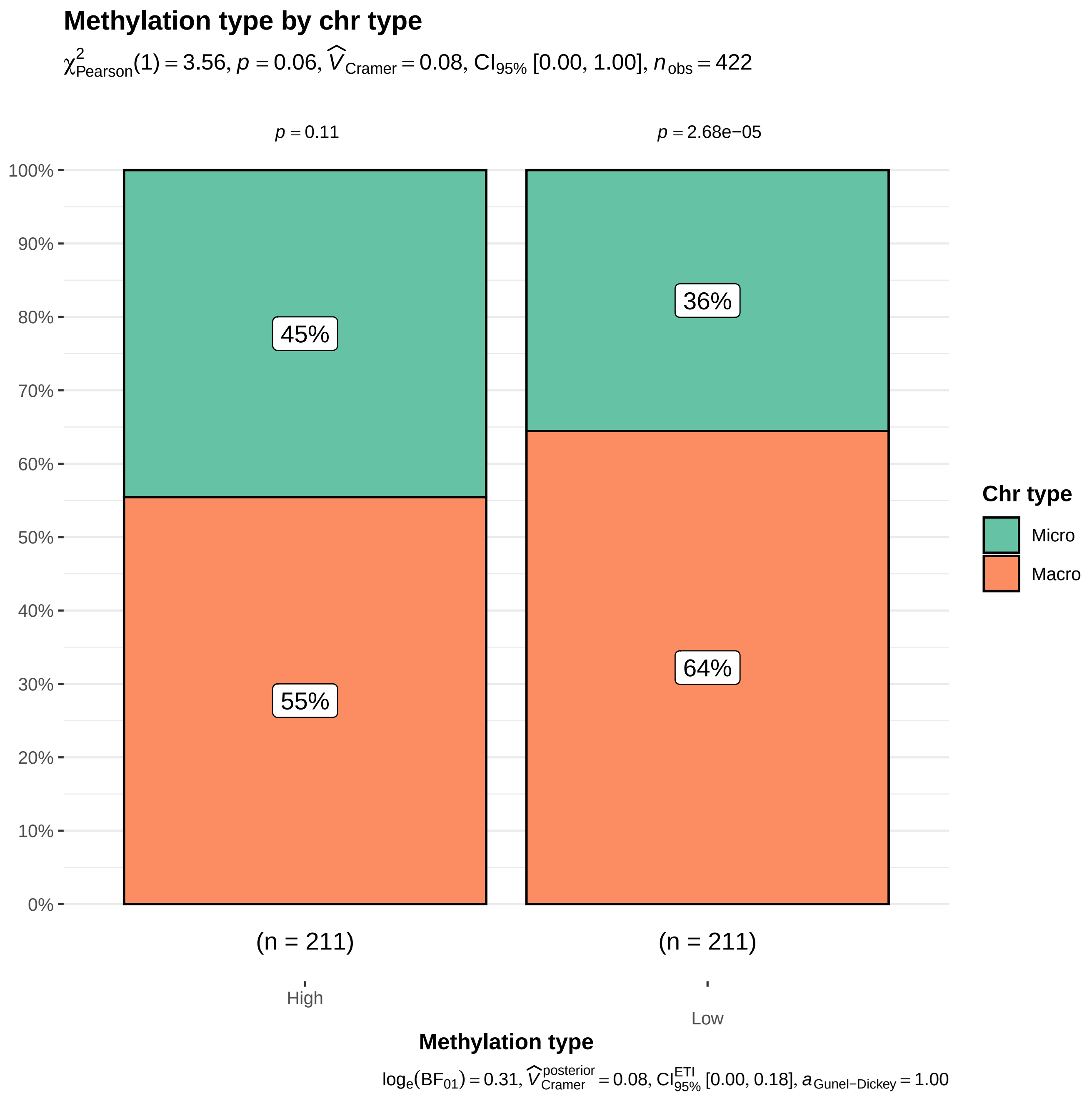

Supplementary Figure 18. The test of top 0.01% and low 0.01% methylation window in macro and micro chromosome. Chi-square test shows that the hypomethylation window was unevenly distributed on macro and micro chromosomes.

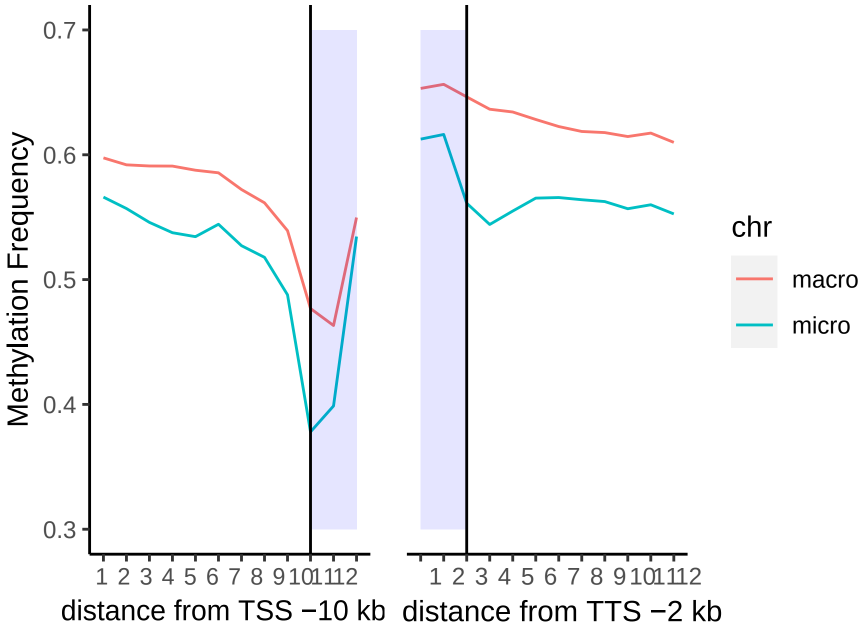

Supplementary Figure 19. Methylation frequency of 10 kbp upstream and downstream of the gene. The horizontal axis is the distance from the TSS and TSS, and the vertical axis is the methylation frequency. The area in purple is gene body. The black vertical lines represent the location of TSS and TTS sites respectively.

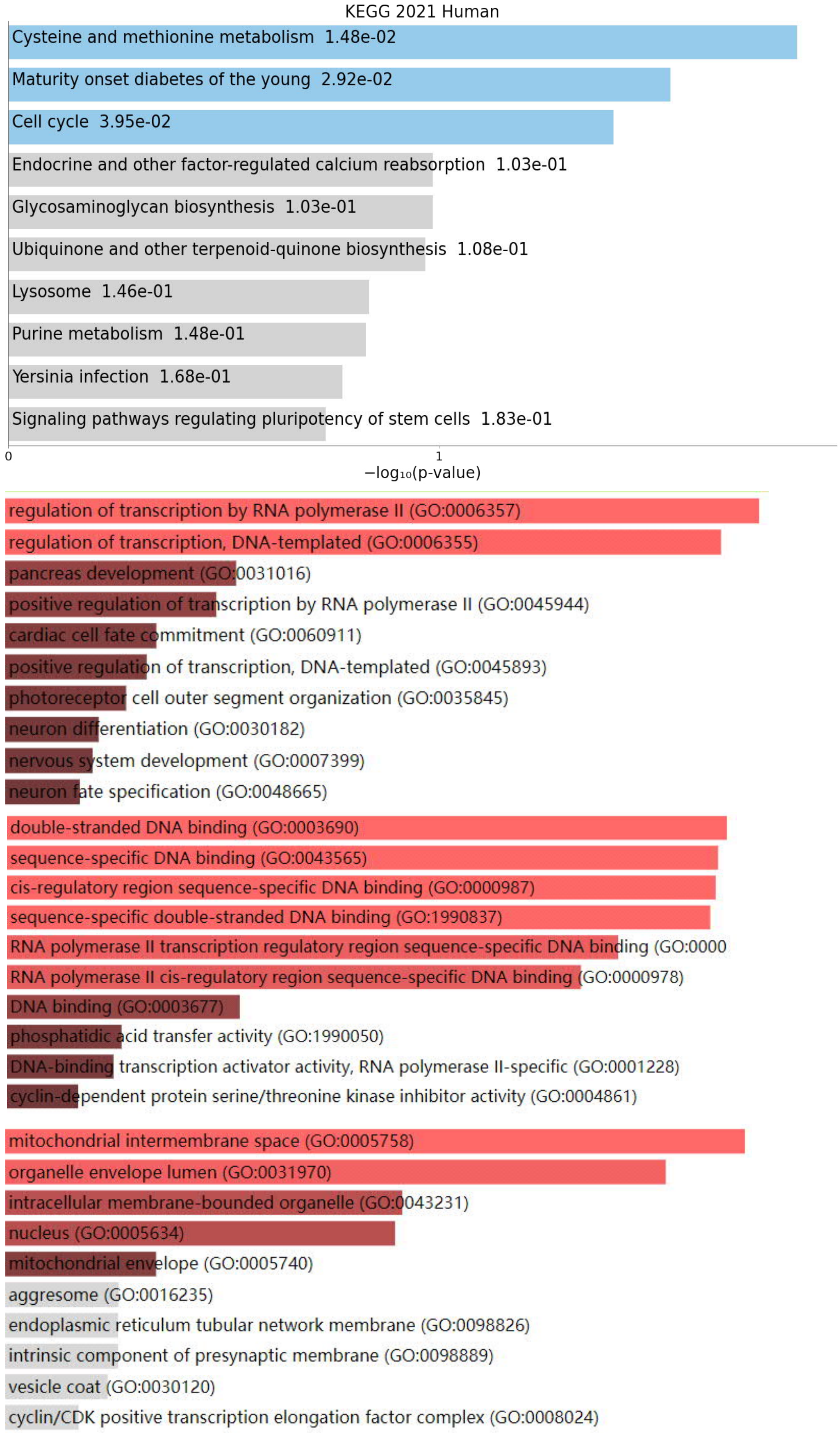

Supplementary Figure 20. The KEGG pathways with its P value, GO terms are significantly enriched for genes in the last 1% of the window with the lowest methylation levels in the whole genome. The blue and red ones are significantly enriched. (Biological process BP, molecular function MF and cellular component CC)

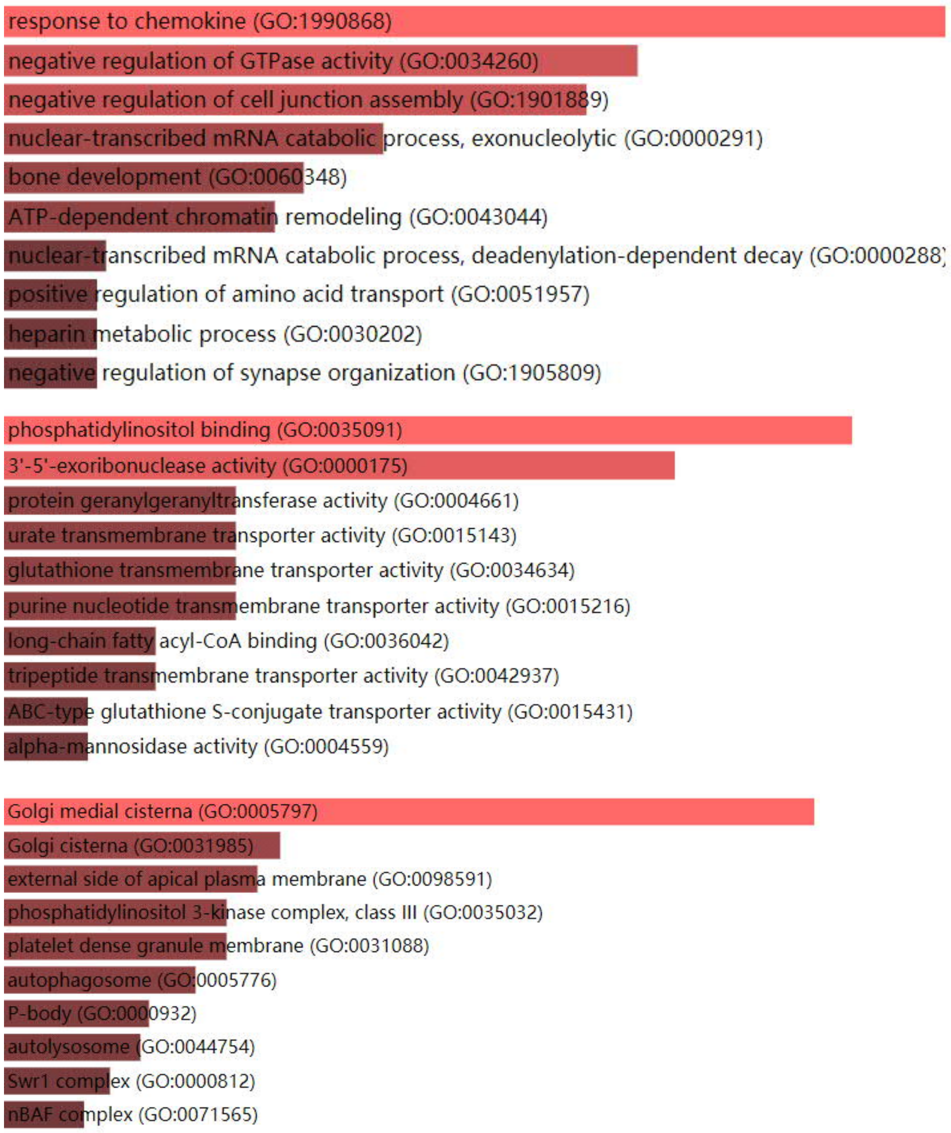

Supplementary Figure 21. The GO terms (BP, MF and CC) are significantly enriched for genes in the top 1% of the window with the lowest methylation levels in the whole genome. The red ones are significantly enriched.

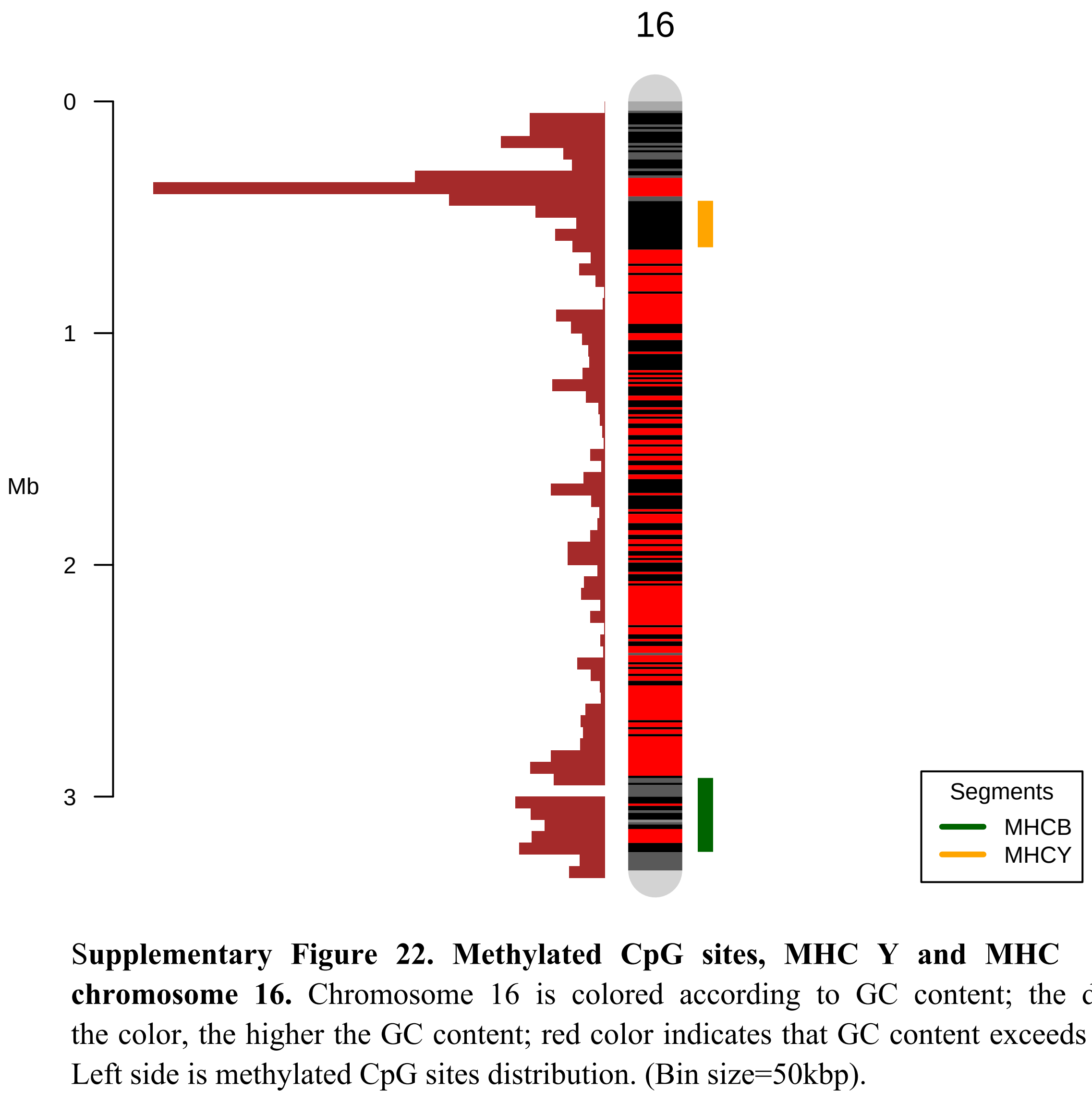

Supplementary Figure 22. Methylated CpG sites, MHC Y and MHC B of chromosome 16. Chromosome 16 is colored according to GC content; the darker the color, the higher the GC content; red color indicates that GC content exceeds 60%. Left side is methylated CpG sites distribution. (Bin size=50kbp).

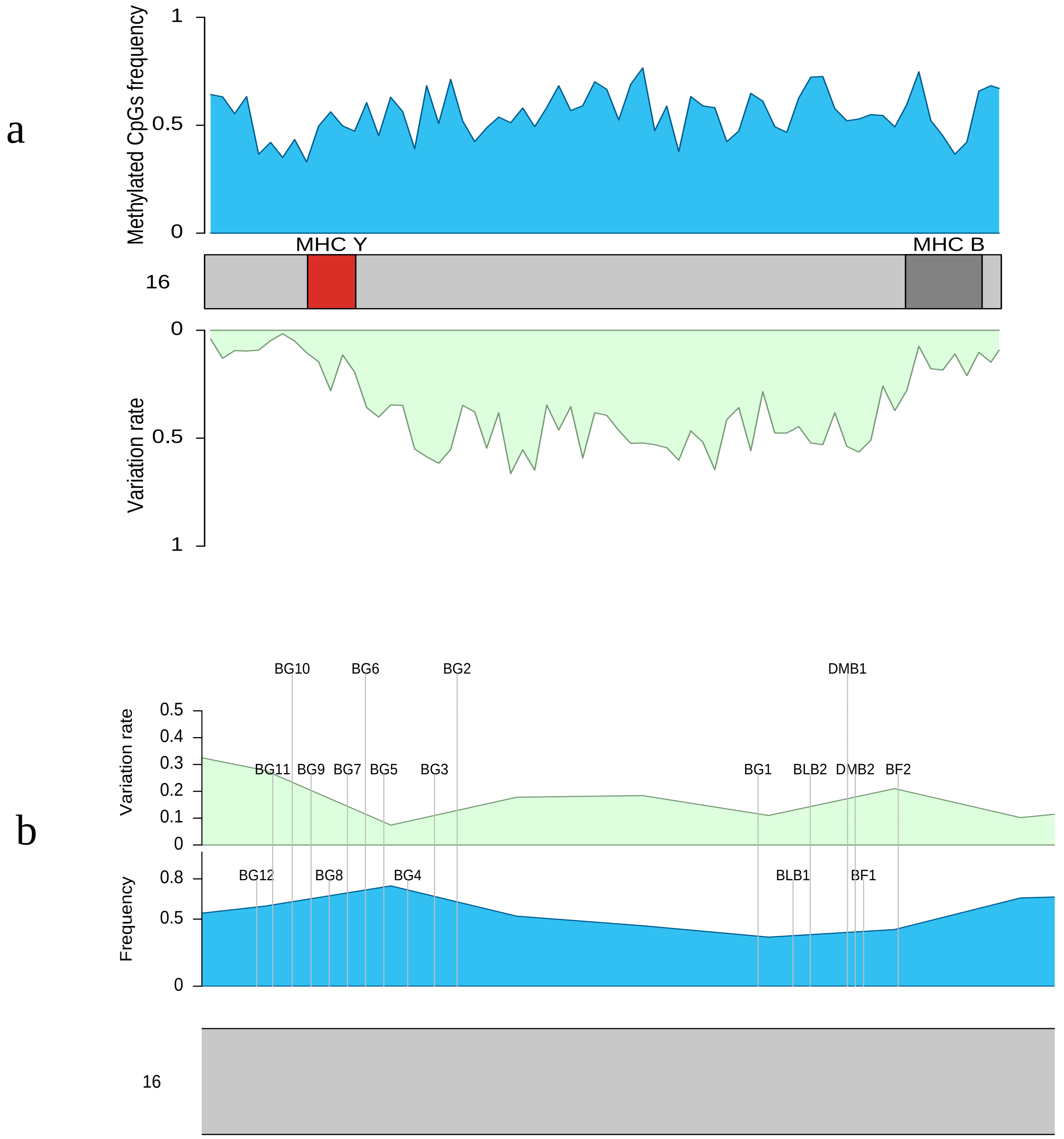

Supplementary Figure 23. Methylation frequency and mutation rate of bases of Silkie in chromosome 16. a. The red area is the MHC Y region and the dark gray area is the MHC B region. b. Methylation frequency and mutation rate of bases of immune-related genes in the MHC B region.

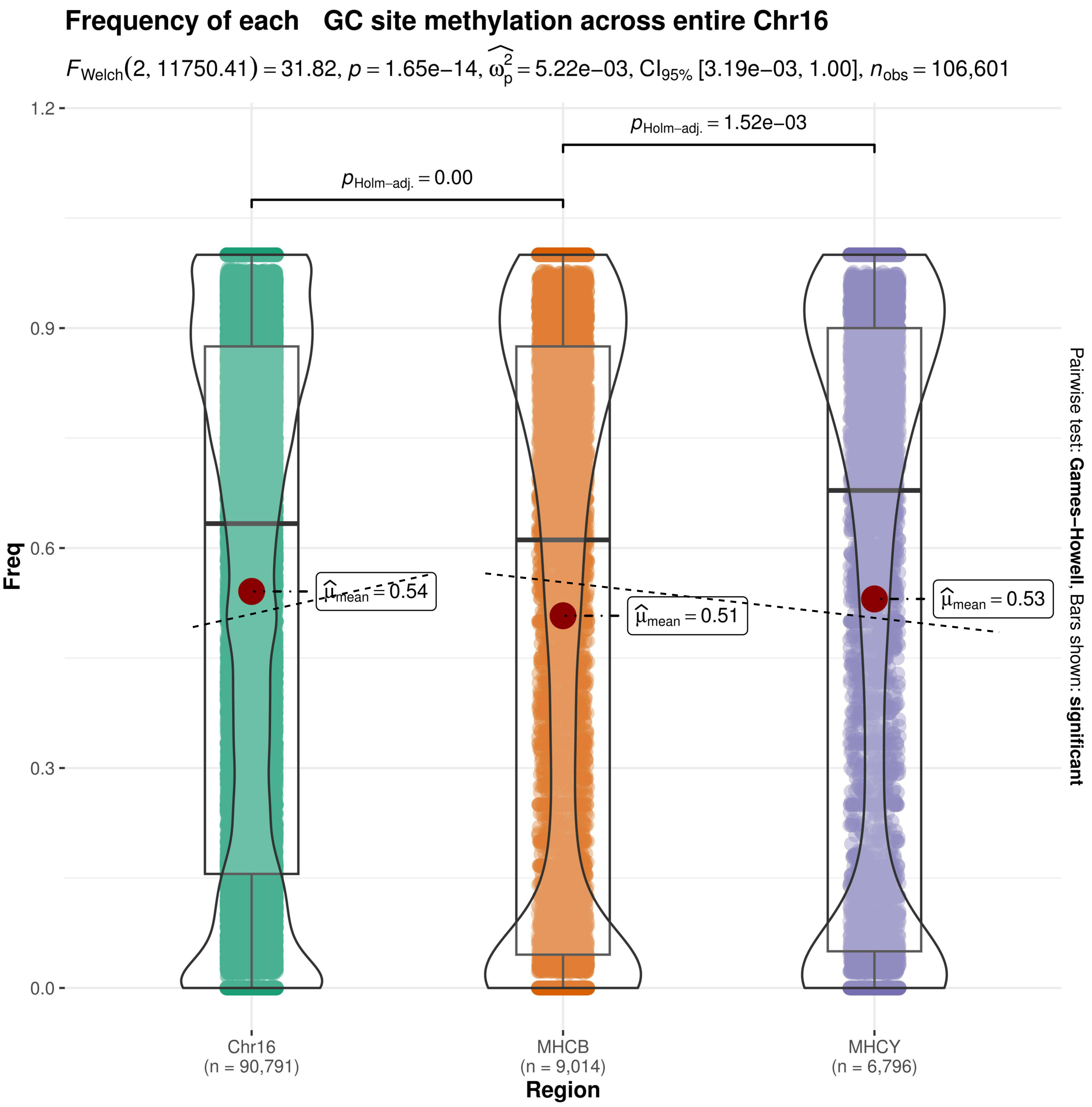

Supplementary Figure 24. Frequency of echo GC site methylation across entire Chr16. Pairwise test (Games-Howell) shows lower methylation MHC B region than entire Chr16 and MHC Y region. The box represents the interquartile range (IQR) of the data, with a line inside the box indicating the median. The "whiskers" extend from the box to the minimum and maximum values within 1.5 times the IQR.

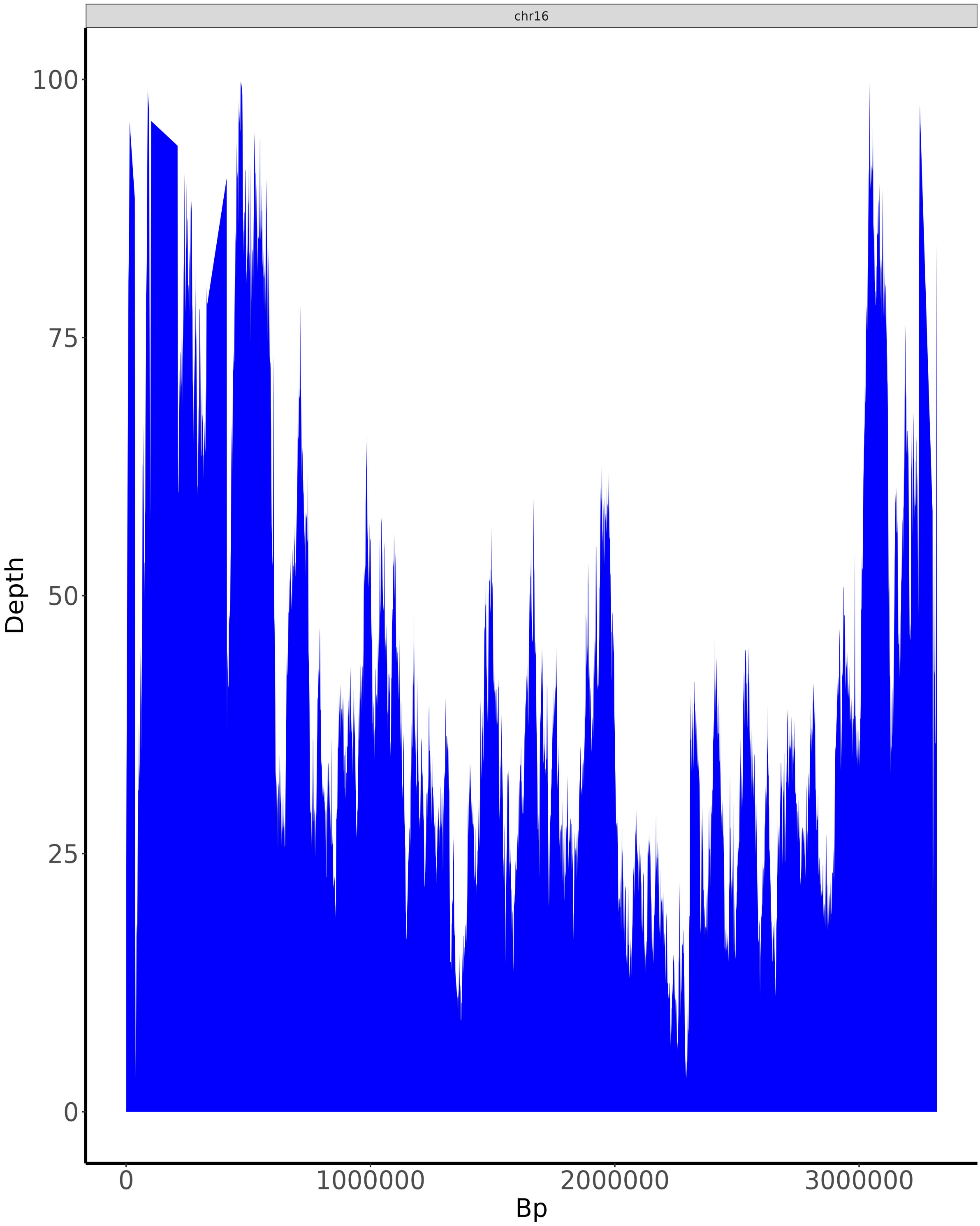

Supplementary Figure 25. The depth generated by mapping with ONT reads.
The depth was filtered by clip length < 100, and the `ymax` for plot was set as 100.

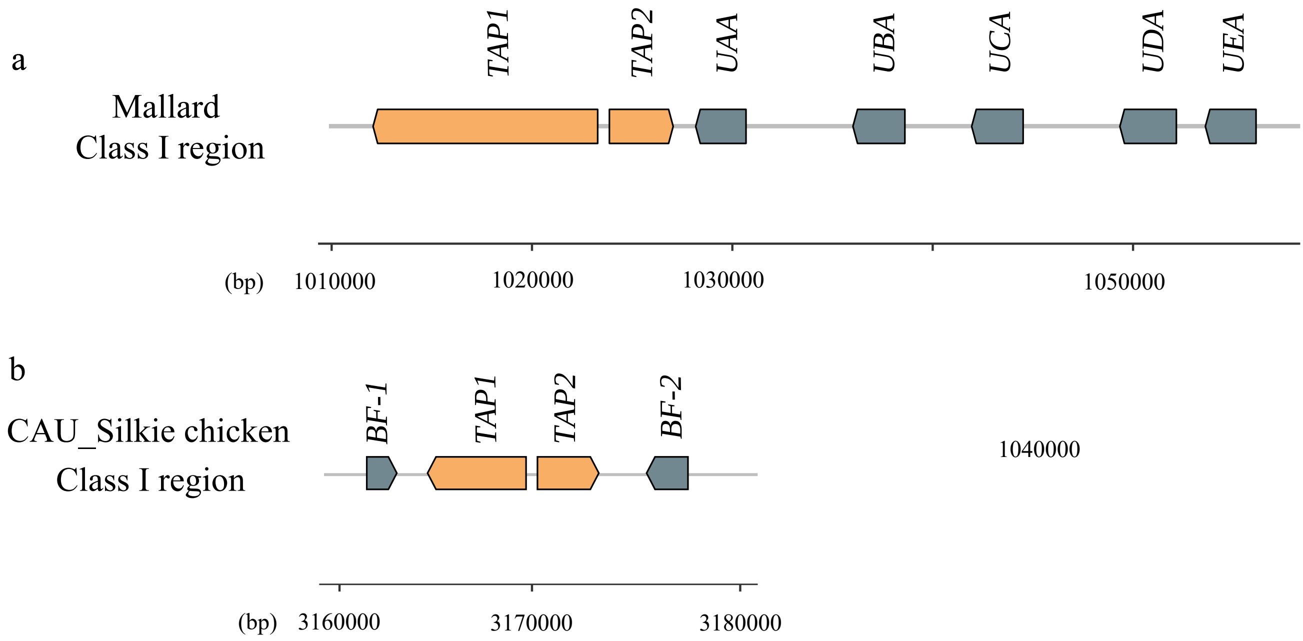

Supplementary Figure 26. Composition and arrangement of core genes in MHC class I region of CAU_Silkie (a) and Mallard (b). The arrow represents the direction geneof transcription; Orange represents *TAP* gene family; Grey represents *BF* gene family.

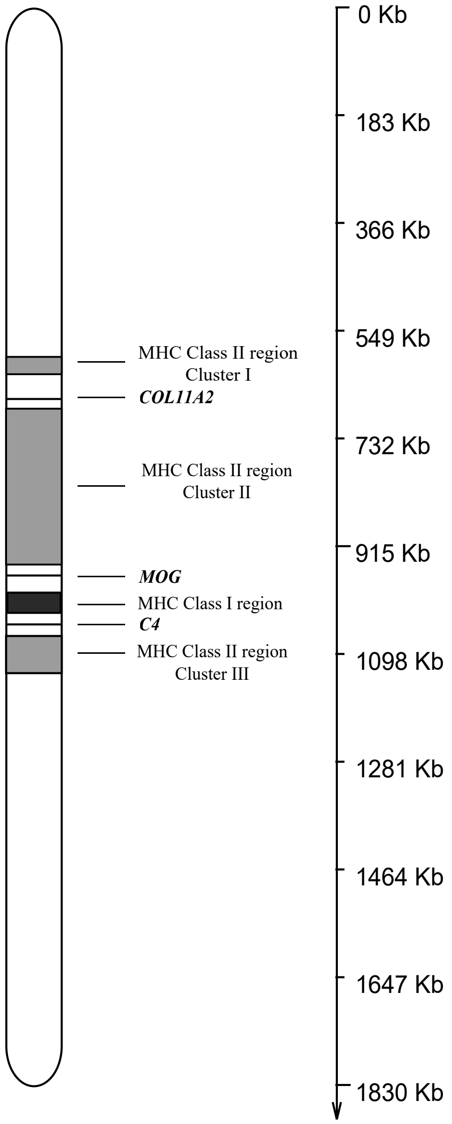

Supplementary Figure 27. The physical location of the three clusters of the MHC class II region in Mallard. Light grey represents three clusters of MHC Class II region ; Dark grey represents MHC Class I region.

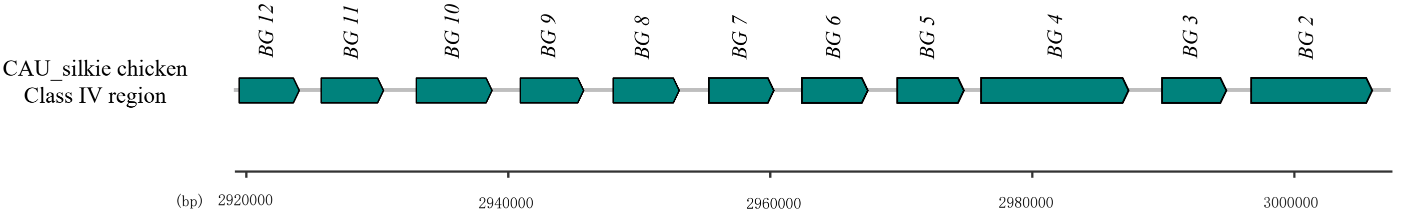

Supplementary Figure 28. Location distribution of 11 gene members of BG gene family on chromosome 16 of Silkie. The arrow represents the direction gene transcription; Green represents *BG* gene family.

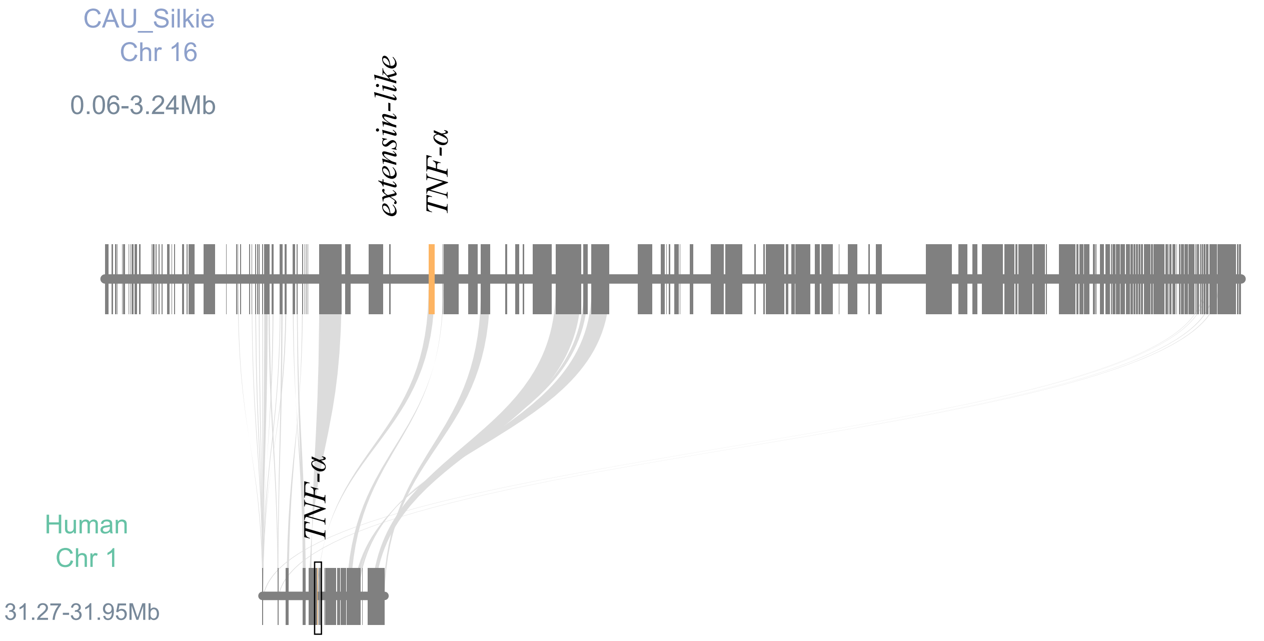

Supplementary Figure 29. Gene colinearity of TNF gene region between Silkie and Human. There are homologous genes TNF-α (CAU_Silkie) with TNF-α (human) , the Silkie extensin-like gene does not show good collinearity with Human.

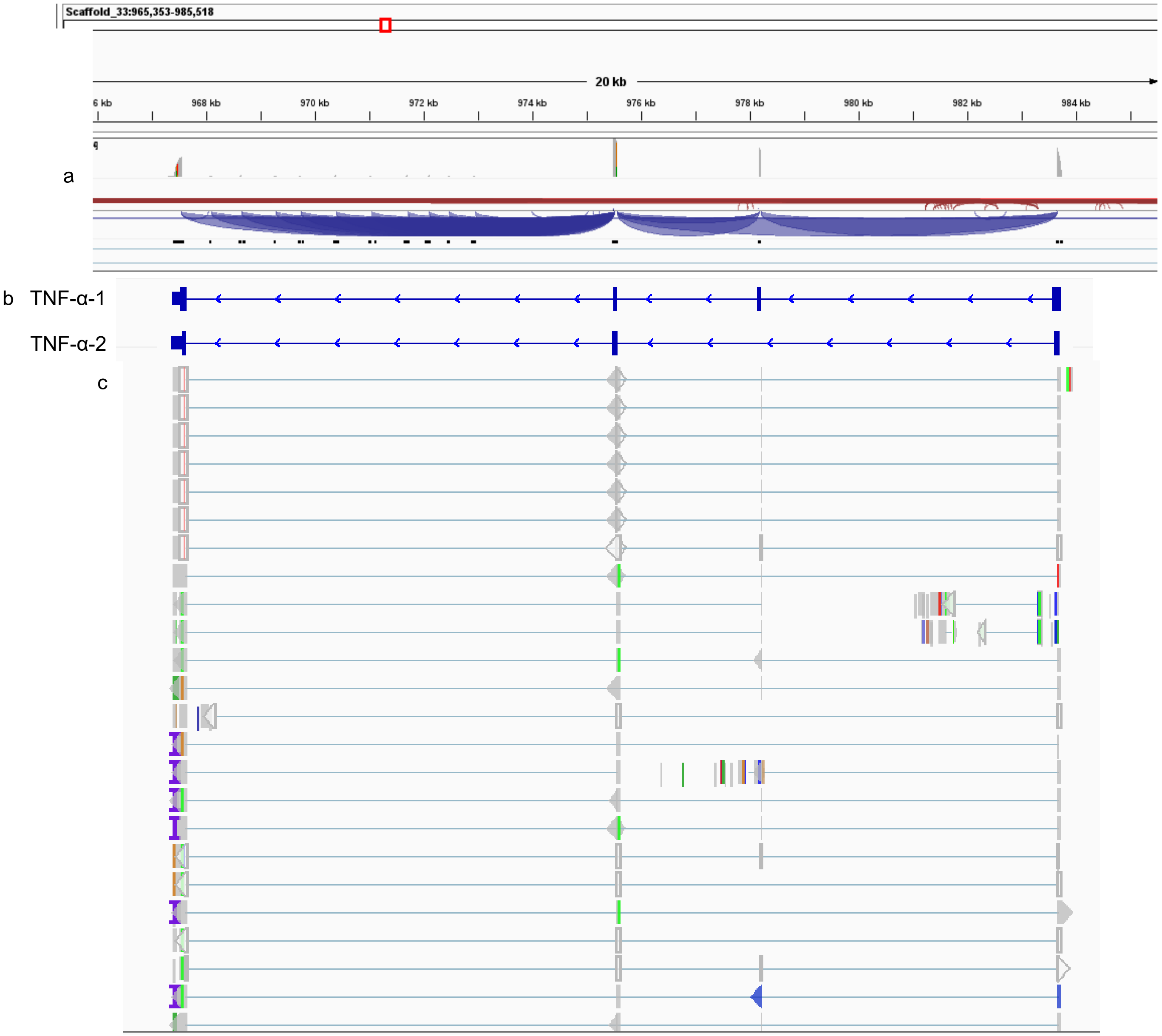

Supplementary Figure 30. The reads coverage map and alternative splicing of two transcript assembly; The reads coverage track of exon in transcript(a); The transcript structure of TNF-α(b); The reads alignment track of exon in transcript(c).

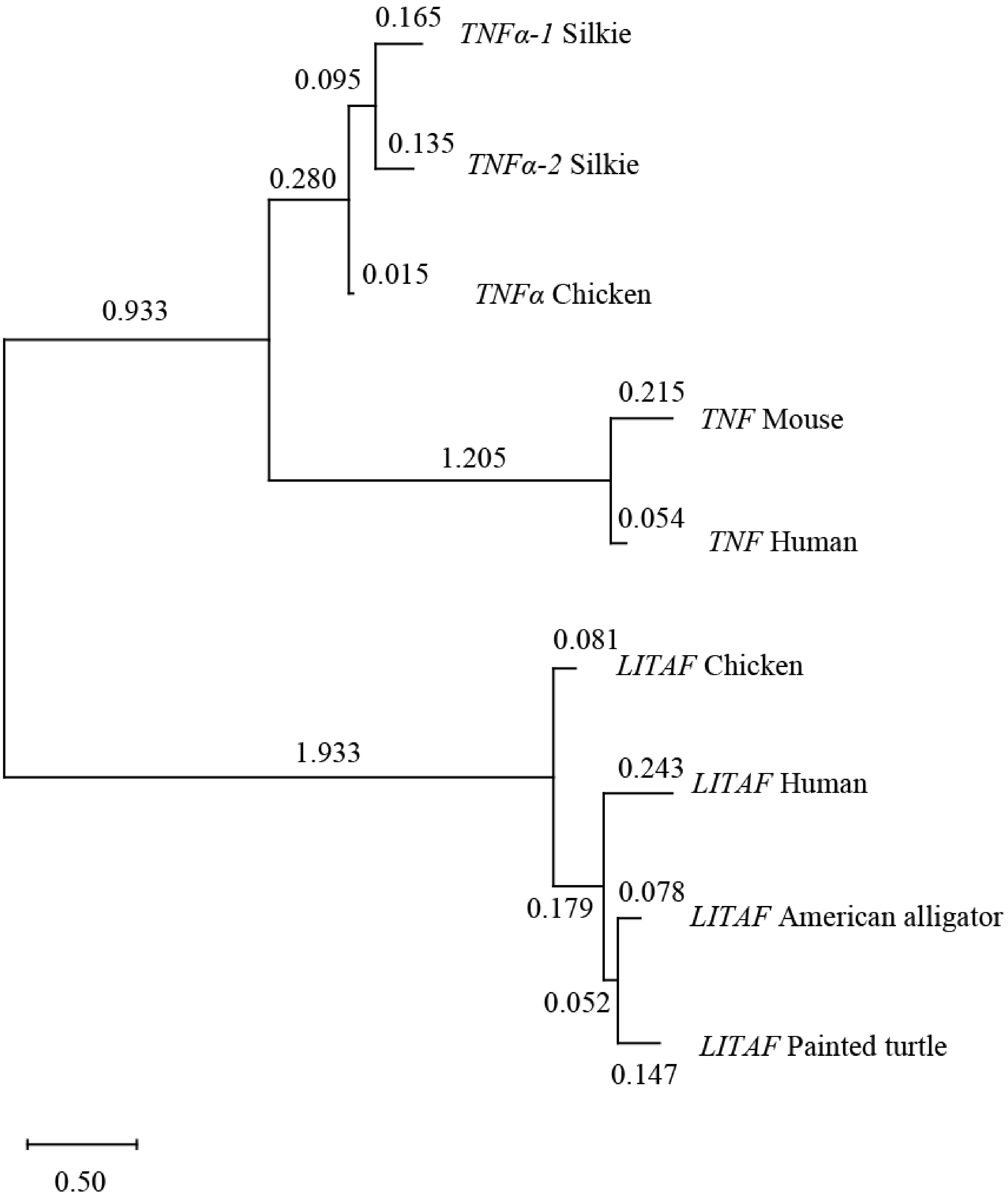

Supplementary Figure 31. Evolutionary tree of *TNF-α* and *LITAF* homologous genes between species. *TNF-α* and *LITAF* in seven major vertebrates (Chiken, Silkie, Mouse, Human, Aligator,Turtle), representing the diagram of gevolutionary relationships.The tree is drawn to scale, with branch lengths measured in the number of substitutions per site (next to the branches).

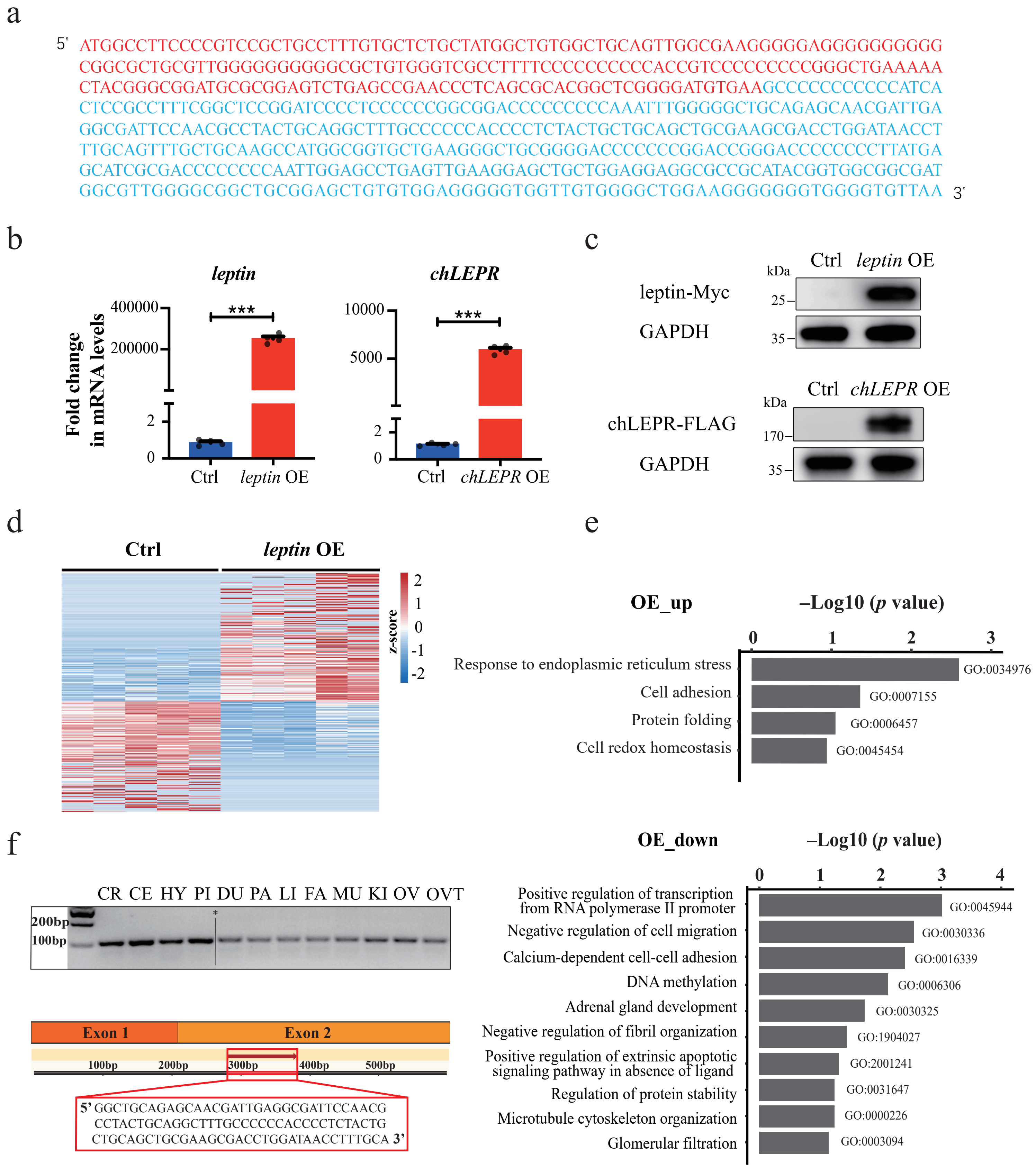

Supplementary Figure 32. Analysis of chicken leptin sequence and function. **a**. Coding sequence of chicken leptin. **b**. qRT-PCR measures mRNA levels of leptin and chLEPR in DF-1 cells under indicated conditions. n = 4 independent experiments. Error bars indicate mean + SE. Statistical analyses were performed by two-tailed Student's t test, ***p < 0.001. **c**. Immunoblotting of over-expression leptin-Myc and chLEPR-Flag levels in DF-1 cells. GAPDH serves as a loading control. **d.** Heat map of the differentially expressed genes in ICP-1 cells cultured under indicated conditions. **e**. GO enrichment analysis of genes upregulated or downregulated in ICP-1 cells overexpressing leptin. **f**. Representative DNA gel and sequence analysis showing leptin qRT-PCR products amplified from cDNA of indicated Silkie tissues. * The line denotes where cropped blots were spliced together in the image.

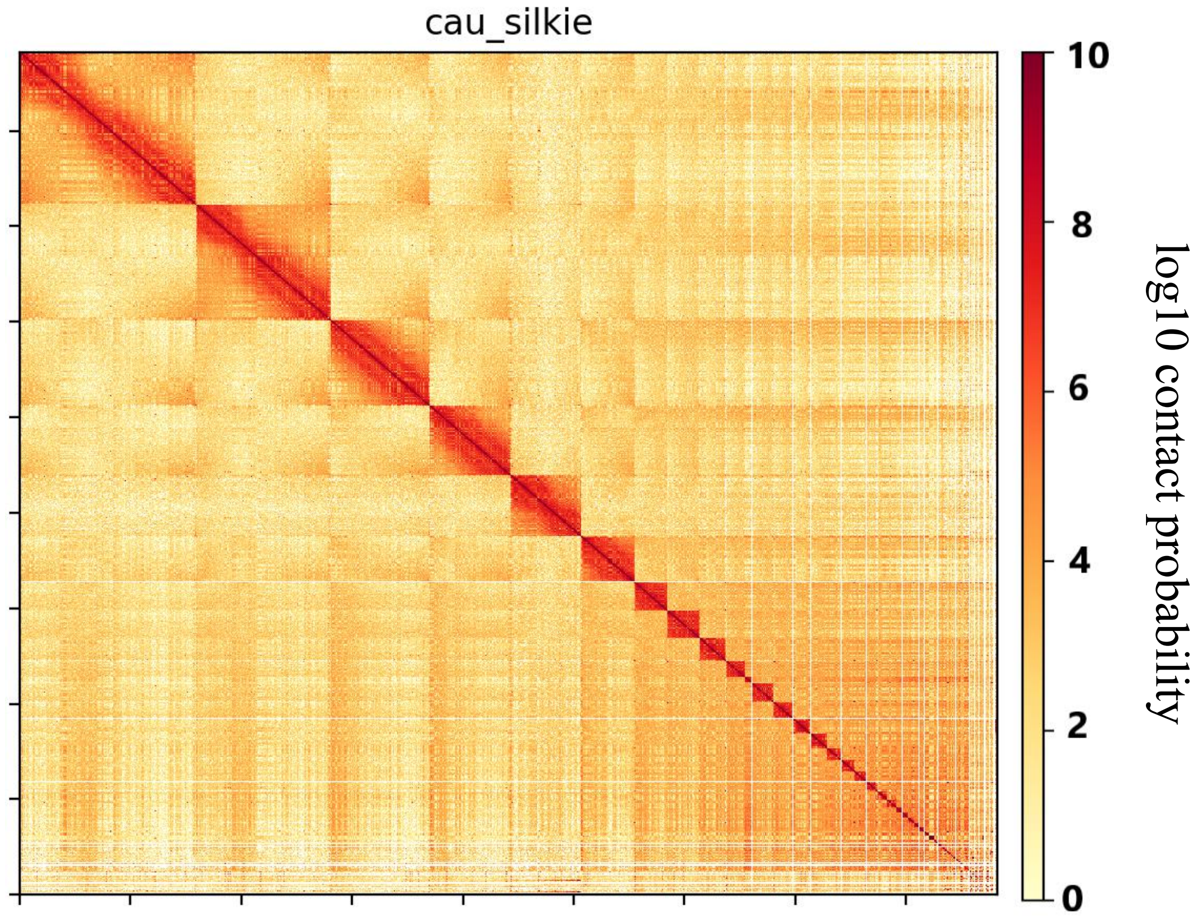

Supplementary Figure 33. Chromatin interactions in each chromosome of CAU_Silkie. The Hi-C data were mapped to the CAU_silkie_chicken genome. Heatmap is shown at a resolution of 500 Kb. The dark red dots show a high probability of interaction, and light yellow show a low probability of interaction.

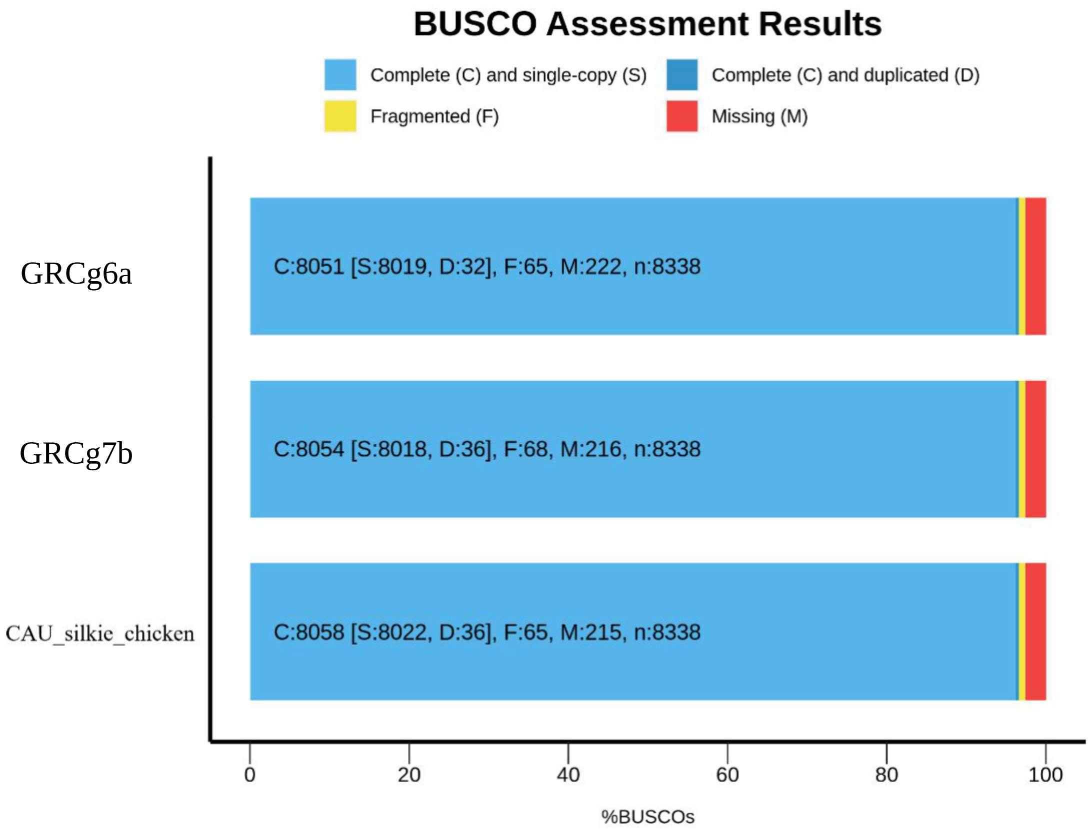

Supplementary Figure 34. BUSCO scores results. BUSCO (Benchmarking Universal Single-Copy Orthologs) attempts to provide a quantitative assessment of the completeness in terms of expected gene content of a genome assembly. The results are simplified into categories of Complete BUSCOs (C), Complete and single-copy BUSCOs (S), Complete and duplicated BUSCOs (D), Fragmented BUSCOs (F), and Missing BUSCOs (M) and total BUSCO groups searched (n) in avian.

Supplementary Figure 35. uncropped gels for Figures

Size markers and uncropped versions of the blots in Fig 4e

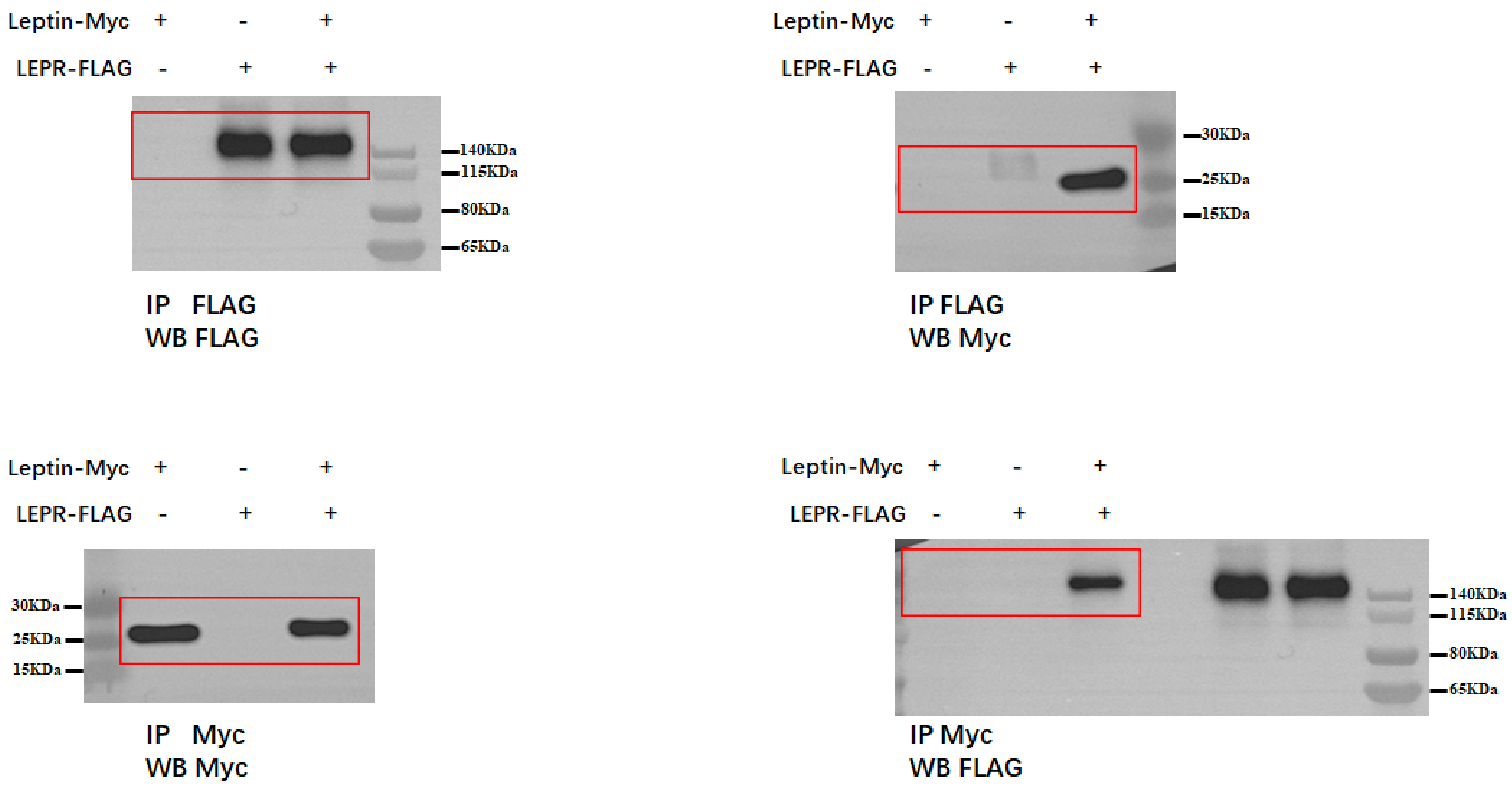

Size markers and uncropped versions of the blots Supplementary Fig 32c

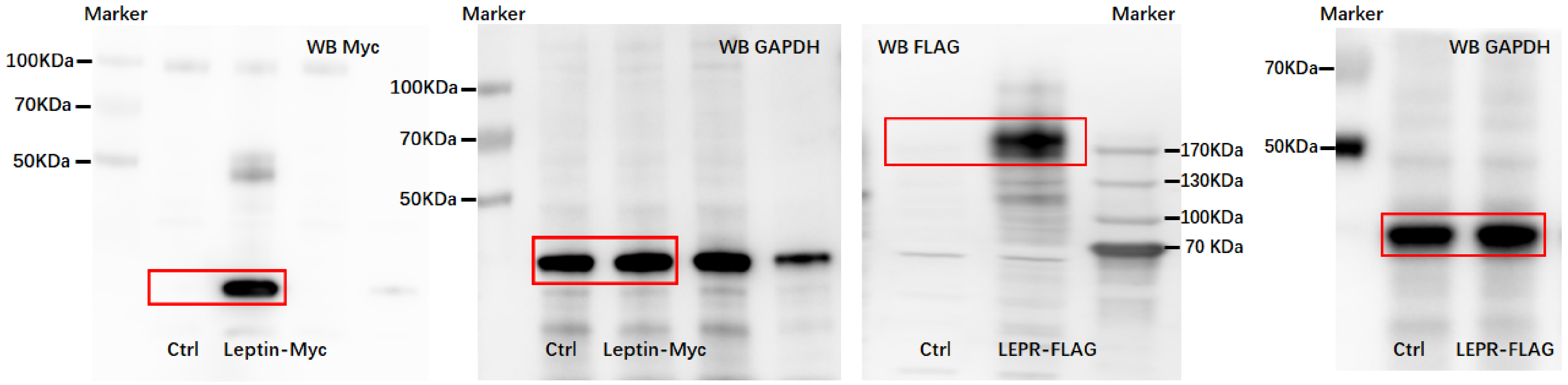

Size markers and uncropped versions of the gels in Supplementary Fig 32f

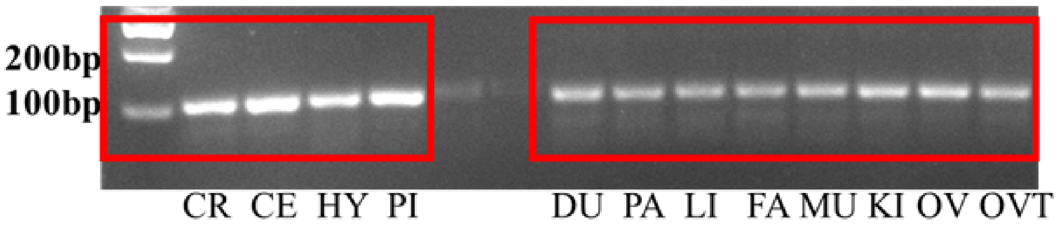

Supplementary Table 1: Assembly comparison with chicken reference genome

Assembly	CAU_Silkie	GRCg6a
Total length including Ns(bp)	1,080,256,408	1,065,348,650
Number of pseudo-chromosome	39	34
Total length of pseudo-chromosome(bp)	1,039,802,905	1,050,139,823
Largest scaffold(bp)	198,715,114	197,608,386
Number of scaffolds	78	524
Scaffold N50(bp)	91,511,380	20,785,086
Scaffold L50	4	12
Number of gaps	11	946
Ns length(bp)	5,025	9,784,460

Supplementary Table 2: Summary of FISH mapping for CAU_Silkie

BAC clone*	Source Location(Chr)	Length (bp)	CAU_Silkie_Chr	CAU_Silkie_Alignment length (bp)	CAU_Silkie_Identity (%)
CH261-97F21	16	1040	16	867	95.04
CH261-100E5	27	1086	27	959	96.14
CH261-107E2	1	1175	1	1000	95.40
CH261-118M1	1	1002	1	955	94.66
CH261-119K2	1	1060	1	853	96.60
CH261-120J2	1	1066	1	911	94.95
CH261-184E5	1	1348	1	1176	94.05
CH261-25P18	1	1139	1	1048	94.37
CH261-29N14	1	1086	1	1066	94.18
CH261-83O13	1	1025	1	856	95.91
CH261-98G4	1	1009	1	964	95.75
CH261-123O22	2	1016	2	903	96.12
CH261-172N3	2	1210	2	985	94.92
CH261-1J20	2	645	2	614	96.09
CH261-40G6	2	1209	2	1072	92.44
CH261-44H14	2	1152	2	1077	93.97
CH261-50C15	2	1120	2	907	92.06
CH261-115J5	3	1055	3	920	97.94
CH261-120H23	3	954	3	786	97.33
CH261-130M12	3	1073	3	881	96.25
CH261-160I6	3	1014	3	831	95.79
CH261-97P20	3	1022	3	836	96.53
CH261-111A15	4	786	4	922	95.88
CH261-183B15	4	1365	4	1164	93.21
CH261-18C6	4	1159	4	1145	92.31
CH261-89P6	4	989	4	837	97.49
CH261-93H1	4	1042	4	887	97.41
CH261-122F8	5	1101	5	919	95.32
CH261-49B22	5	1059	5	937	92.53
CH261-165L8	6	1136	6	953	91.50
CH261-179F2	6	966	6	869	97.58
CH261-49F3	6	1154	6	1090	96.06
CH261-94G14	6	1125	6	991	95.96
CH261-180H18	7	1078	7	910	96.70
CH261-186K14	7	1128	7	1048	95.23
CH261-38E18	7	1174	7	967	92.66
CH261-34H16	8	1221	8	1186	92.66
CH261-187M16	9	1158	9	951	96.32
CH261-115G24	10	906	10	860	96.63
CH261-118E15	10	992	10	929	96.34
CH261-71G18	10	1154	10	1079	93.51
CH261-94C12	10	1067	10	1049	98.57
CH261-121N21	11	1086	11	925	97.30
CH261-138H13	11	991	11	977	97.65
CH261-154H1	11	1131	11	966	96.38
CH261-152H14	12	1010	12	1009	98.71
CH261-4M5	12	1098	12	1000	95.70
CH261-88K1	12	1039	12	933	96.89
CH261-90N18	12	998	12	903	97.34
CH261-95H20	12	1099	12	1036	93.73
CH261-115I12	13	990	13	962	96.36
CH261-11H24	13	1061	13	852	95.31
CH261-71N1	13	1093	13	938	93.28
CH261-69D20	14	997	14	806	96.90
CH261-131E4	15	1074	15	932	96.35
CH261-40D6	15	1021	15	951	96.85
CH261-48M1	15	919	15	880	93.18
CH261-113A7	17	988	17	860	97.91
CH261-42P16	17	1037	17	829	96.62
CH261-69M11	17	1195	17	979	90.50
CH261-72P11	17	1105	17	910	97.03
CH261-118D24	18	982	18	806	96.15
CH261-137B21	18	1058	18	1009	98.22
CH261-67N15	18	1197	18	941	97.24
CH261-72B18	18	1055	18	1027	95.81
CH261-10L6	20	1339	20	1183	94.59
CH261-124A24	20	971	20	873	97.82
CH261-122K8	21	1145	21	1035	94.78
CH261-49L18	21	1164	21	1050	94.38
CH261-18G17	22	1180	22	1179	94.06
CH261-30D24	22	1127	22	899	94.66
CH261-49B2	22	1155	22	936	95.30
CH261-105P1	23	1003	23	880	97.05
CH261-49G9	23	1170	23	1021	94.71
CH261-90K11	23	1034	23	915	97.27
CH261-154H17	24	1158	24	1053	95.73
CH261-65O4	24	1070	24	1048	94.85
CH261-127K7	25	1007	25	959	96.14
CH261-169N16	25	196	25	189	95.24
CH261-186M13	26	1147	26	922	96.20
CH261-40C14	26	1162	26	970	94.12
CH261-50J5	26	1159	26	996	91.97
CH261-66M16	27	1155	27	1080	90.65
CH261-101C8	28	1074	28	889	96.96
CH261-186C5	28	1127	28	1043	95.11
CH261-129A16	Z	1103	Z	1075	95.07
CH261-133M4	Z	1068	Z	942	96.82
CH261-137F19	Z	1073	Z	940	97.02

[1] O'Connor R E, Kiazim L, Skinner B, et al. Patterns of microchromosome organization remain highly conserved throughout avian evolution[J]. Chromosoma, 2019, 128(1): 21-29.
[2] Solinhac R, Leroux S, Galkina S, et al. Integrative mapping analysis of chicken microchromosome 16 organization[J]. BMC genomics, 2010, 11(1): 1-12.

Supplementary Table 3: Characterization of genes in Silkie Chiken genomes.

	**Silkie(CAU Silkie)**
Gene number	18034
Total gene Length (bp)	402557831
Average gene length	22322.15986
Total exon length (bp)	36291827
Average exon length	169.0405696
Total CDS length (bp)	36291827
Average CDS length	169.0405696
Average exons per gene	11.90490185
Average intron length(bp)	1870.664033

Supplementary Table 4: Summary of gene annotation by using different databases

-	silkie(CAU silkie)	
	**Gene number**	**Percentage%**
GO	12,145	67.3
KEGG	13,678	75.8
Swissprot	14,556	80.7
NR	17,499	97.03

Supplementary Table 5: Structural variations in CAU_Silkie genome.

Total Structural variations *	23561
SNP	8408966
INDEL (<=50bp)	591967
Deletion	1503
Duplication	384
Insertion	120
Invertion	686
Translocations (intra-chromosome)	72
Translocations (inter-chromosome)	49

* SVs > 50bp

Supplementary Table 6: The number of methylated CpG sites in chromosomes of CAU_Silkie

Chromosome	CpG sites number	Cs number	Methylation ratio
1	852170	40154949	0.021
2	631520	30563767	0.021
3	463529	22216986	0.021
4	384034	18488079	0.021
5	291177	12333947	0.024
6	195851	7512953	0.026
7	195868	7741365	0.025
8	171826	6185958	0.028
9	137804	5086595	0.027
10	178255	4702909	0.038
11	131757	4329073	0.030
12	179589	4563892	0.039
13	106747	4030675	0.026
14	122987	3518693	0.035
15	98838	2956346	0.033
16	43062	714995	0.060
17	88934	2541467	0.035
18	93433	2787648	0.034
19	96211	2582321	0.037
20	101262	3377606	0.030
21	81899	1986493	0.041
22	69204	1433008	0.048
23	65720	1506457	0.044
24	58504	1602374	0.037
25	55480	757999	0.073
26	63094	1367366	0.046
27	62768	1466518	0.043
28	64977	1218130	0.053
29	29630	513768	0.058
30	53150	662107	0.080
31	31899	627370	0.051
32	37275	623307	0.060
33	22078	1221405	0.018
34	79801	933890	0.085
35	19231	309100	0.062
36	26122	418348	0.062
37	31799	410834	0.077
38	43271	579054	0.075
Z	368071	16148778	0.023

Supplementary table 7:Major distribution of novel assembled sequence on chromosome 16 of Silkie

	Length(bp)	Gene number	Start (bp)	End (bp)
	591071	11	642947	1234018
	120985	3	1313451	1434436
	76306	1	1506808	1583114
	40714	2	1858322	1899036
	439057	11	1921377	2360434
	73817	2	2441119	2514936
	77905	3	2579170	2657075
	185153	10	2704905	2892020
Total	1605008	43		

Supplementary table 8:The protein-coding genes annotated in novel sequences

Gene symbol	Gene description	Human Ensembl ID
DDX39B	Spliceosome RNA helicase DDX39	ENSG00000198563
BAG6 *^	Large proline-rich protein BAG6	ENSG00000204463
BAG6 *^	Large proline-rich protein BAG6	ENSG00000204463
extensin-like	extensin-like	
TNFA	TNF-alpha	ENSG00000228978
GPANK1*	G patch domain and ankyrin repeat-containing protein 1	ENSG00000204438
CSNK2B *	casein kinase 2 beta	ENSG00000204435
CFBL *	complement factor b, like	ENSG00000243649
CFBL *	complement factor b, like	ENSG00000243649
MGAT1 *	alpha-1,3-mannosyl-glycoprotein 2-beta-N-acetylglucosaminyltransferase	ENSG00000131446
PLCA *	1-acyl-sn-glycerol-3-phosphate acyltransferase alpha	ENST00000395499
EHMT1 *	euchromatic histone lysine methyltransferase 1	ENSG00000181090
VARS1	Valine--tRNA ligase	ENSG00000204394
LSM12 *^	U6 snRNA-associated Sm-like protein LSm2	ENSG00000161654
ABHD16A *	abhydrolase domain-containing protein 16A	ENSG00000204427
SELENOV *	selenoprotein V-like	ENSG00000186838
DHX16*	pre-mRNA-splicing factor ATP-dependent RNA helicase DHX16	ENSG00000204560
ATAT1*^	alpha tubulin acetyltransferase 1	ENSG00000137343
PPP1R10*	protein phosphatase 1 regulatory subunit 10	ENSG00000204569
ABCF1*^	ATP-binding cassette sub-family F member 1-like	ENSG00000204569
TRIM39	tripartite motif containing 39	ENSG00000204599
ZBTB12*^	zinc finger and BTB domain containing 12	ENSG00000204366
RXRBA * '	retinoid x receptor, beta a	ENSG00000204231
WDR46 * '	WD repeat domain 46	ENSG00000227057
VPS52 * '	vacuolar protein sorting-associated protein 52 homolog	ENSG00000223501
RPS5*	ribosomal protein S5	ENSG00000083845
TUBB	Tubulin Beta Class I	ENSG00000196230
CCHCR1*^	coiled-coil alpha-helical rod protein 1	ENSG00000204536
SYNGAP1 *^ '	synaptic Ras GTPase activating protein 1	ENSG00000197283
CUTA * '	protein CutA	ENSG00000112514
C6orf136*^	chromosome 6 open reading frame 136	ENSG00000204564
MRPS18B*^	mitochondrial ribosomal protein S18B	ENSG00000204568
ABCF1*	ATP-binding cassette sub-family F member 1-like	ENSG00000204574
GNL1*	G protein nucleolar 1	ENSG00000204590
POLR2A*	RNA polymerase II subunit A	ENSG00000181222
BLA*	major histocompatibility complex class II alpha chain BLA	ENSG00000204257
COL11A1	collagen type XI alpha 1 chain	ENSG00000060718
SLC39A7 *^ '	solute carrier family 39 member 7	ENSG00000112473
RPS5*	ribosomal protein S5	ENSG00000083845
GABBR1*	gamma-aminobutyric acid type B receptor subunit 1	ENSG00000204681
FLOT1	Flotillin 1	ENSG00000137312
MDC1*	mediator of DNA damage checkpoint 1	ENSG00000137337
ZBTB9*	zinc finger and BTB domain containing 9	ENSG00000213588
SYNGAP1*^ '	synaptic Ras GTPase activating protein 1	ENSG00000197283
PHF1*	PHD finger protein 1	ENSG00000112511

* The genes were newly discovered in chromosome 16 of silkie

^ The genes were thought to be missing in birds

' The genes were found in the chicken pan-genome

Supplementary Table 9: Summary of raw reads from PacBio Sequel II

Sequencing	Cell ID		
	m64061 201030 024601	m64066 200930 085914	m64082 201009 073749
Polymerase read bases (Gb)	275.90	271.64	228.98
Polymerase reads	4,165,072	3,854,617	3,872,628
Unique molecular yield (Gb)	59.87	58.09	58.03
Mean polymerase read length (kb)	66.24	70.47	59.13
Polymerase read N50 (kb)	159.19	172.61	157.00
Mean subread length (kb)	11.88	12.13	12.56
Subread N50 (kb)	12.70	12.95	13.50
Mean insert length (kb)	15.32	16.13	15.85
Insert N50 (kb)	15.70	17.36	19.30
Average length (kb)	11.88	12.13	12.56
Average N50 (kb)	12.70	12.95	13.50
Total bases (Gb)	275.02	270.78	228.28
Subreads depth	250.01	246.16	207.53

Supplementary Table 10: Summary of HiFi reads

Data	PacBio HiFi reads		
Length (bp)	Number	Total length (bp)	Percent of length contribution*
(0, 1,000]	818	130,136	0.00%
(1,000, 5,000]	10,040	37,070,630	0.09%
(5,000, 10,000]	21,081	159,907,145	0.37%
(10,000, 15,000]	3,358,927	42,565,279,003	98.71%
(15,000, 20,000]	23,128	358,674,639	0.83%
(20,000, 25,000]	55	1,176,855	0.00%
(25,000, 30,000]	17	455,152	0.00%
Total	3,414,066	43,122,693,560	100.00%
Sequencing depth		39.20	
Total length (Gb)		43.12	
Read N50 (kb)		12.69	
Read number		3,414,066	
Mean length (kb)		12.63	
Max length (kb)		29.36	

*Percent of length contribution = length of one interval / total length

Supplementary Table 11: Summary of ONT reads

Data	ONT reads			Corrected ONT reads*		
Length (bp)	Number	Total length (bp)	Percent of length contribution	Number	Total length (bp)	Percent of length contribution
(0, 1,000]	1,473,294	834,389,467	0.90%	1,468,822	829,184,902	0.88%
(1,000, 5,000]	2,741,752	6,872,969,472	7.40%	2,733,479	6,858,785,231	7.30%
(5,000, 10,000]	986,076	6,983,491,248	7.52%	988,433	7,000,479,384	7.45%
(10,000, 15,000]	460,718	5,656,904,073	6.09%	461,426	5,665,645,503	6.03%
(15,000, 20,000]	295,748	5,131,593,286	5.52%	295,893	5,134,214,385	5.46%
(20,000, 25,000]	214,289	4,796,891,124	5.16%	214,213	4,794,276,889	5.10%
(25,000, 30,000]	200,676	5,544,742,388	5.97%	194,001	5,353,449,143	5.70%
(30,000, 50,000]	999,836	38,325,618,456	41.25%	1,000,911	38,507,322,352	40.97%
(50,000, 75,000]	256,267	14,981,230,740	16.12%	267,223	15,640,703,112	16.64%
(75,000, 100,000]	33,815	2,843,289,362	3.06%	36,692	3,086,809,520	3.28%
(100,000, 150,000]	7,857	887,919,390	0.96%	9,154	1,038,055,523	1.10%
(150,000, 200,000]	298	49,081,283	0.05%	372	61,263,967	0.07%
(200,000, 250,000]	20	4,170,809	0.00%	27	5,701,347	0.01%
(250,000, 300,000]	5	1,361,958	0.00%	5	1,380,469	0.00%
(300,000, 550,000]	4	1,642,525	0.00%	4	1,661,700	0.00%
Total	7,670,655	92,915,295,581	100.00%	7,670,655	93,978,933,427	100.00%
Sequencing depth		84.47			85.44	
Total length (Gb)		92.92			93.98	
Read N50 (kb)		34.93			34.93	
Read number		7,670,655			7,670,655	
Mean length (kb)		12.11			12.25	
Max length (kb)		547.66			549.75	

* : Reads length less than 1kb were filtered by NextDenovo

Supplementary Table 12: Summary of genome assemblies for CAU_Silkie

Statistics	HiFi Assembly	ONT Assembly	ONT Polished	Hybird Assembly	Hi-C Grouping	Final Assembly
Contig/Scaffold number	1243	180	180	132	116	78
Total length including Ns(bp)	1,121,360,583	1,070,707,953	1,070,707,953	1,126,310,592	1,126,328,092	1,080,553,668
Contig/Scaffold N50 (bp)	12,556,330	48,284,348	48,284,348	72,427,925	79,431,813	91,511,380
Contig/Scaffold L50	26	8	8	6	5	4
Contig/Scaffold N90 (bp)	1,101,206	5,801,169	5,802,151	6,225,192	6,558,973	10,490,610
Contig/Scaffold L90	145	36	36	28	22	20
Contig/Scaffold Max (bp)	58,228,619	98,940,442	98940442	198715114	198715114	198715114
Gap length (bp)	0	0	0	0	17500	5025
Gap Number	0	0	0	0	35	11

Supplementary Table 13: Summary of Hi-C reads mapping

Class	Number of Reads	Percentage
Total read pairs	707,261,351	100%
Uniquely mapped read pairs	190,909,915	27%
Valid interaction pairs	66,573,775	9.41%
Dangling end pairs	110,994,477	15.69%
Re-ligation pairs	9,513,434	1.35%
Self-cycle pairs	1,359,279	0.19%
Dumped pairs	2,468,950	0.35%

Supplementary Table 14: Summary of the Hi-C grouping result in each chromosome

Chr*	Scaffold number	Total length	Final length
chr1	1	198,715,114	198,715,114
chr2	2	151,350,810	151,362,929
chr3	1	98,940,442	111,120,280
chr4	2	91,511,380	91,511,380
chr5	1	59,518,634	59,518,634
chr6	2	35,677,100	35,677,093
chr7	1	36,999,723	36,999,723
chr8	2	29,562,663	29,570,623
chr9	2	23,835,124	23,833,528
chr10	1	21,528,794	21,528,794
chr11	2	20,025,547	20,082,464
chr12	1	21,329,695	21,329,695
chr13	1	17,944,738	17,944,738
chr14	1	15,517,226	15,517,226
chr15	1	12,921,927	12,921,927
chr16	3	3,184,811	3,318,479
chr17	1	10,490,610	10,490,610
chr18	1	12,081,000	12,081,000
chr19	2	10,824,814	10,822,179
chr20	1	14,210,439	14,507,699
chr21	1	7,756,144	7,756,144
chr22	1	5,672,792	5,672,792
chr23	2	6,148,826	6,154,293
chr24	1	6,558,973	6,558,973
chr25	2	3,318,979	3,549,130
chr26	1	5,783,249	5,783,249
chr27	1	6,108,090	6,116,237
chr28	1	5,192,615	5,192,615
chr29	1	2,376,650	2,376,650
chr30	1	2,897,333	3,184,811
chr31	1	2,542,040	2,542,040
chr32	2	2,793,648	2,897,333
chr33	1	4,801,847	4,807,732
chr34	1	3,549,130	4,501,735
chr35	1	1,578,898	1,578,898
chr36	1	2,118,238	2,118,238
chr37	3	1,877,094	1,882,168
chr38	1	2,719,936	2,817,697
chrZ	1	79,431,813	79,431,813

*The pseudo-chromosomes

Supplementary Table 15: Summary of annotation of non-coding RNA in Silkie_chicken

	Type	Copy(w*)	Average length(bp)	Total length(bp)	% of genome
miRNA		254	81.87	20,794	0.001924
tRNA		320	75.52	24,166	0.002236
rRNA	SSU_rRNA 18s	8	903.00	7,224	0.000669
	LSU_rRNA 28s	25	775.36	19,384	0.001794
	5.8S	3	153.00	459	0.000042
	5S	60	119.10	7146	0.000661
snRNA	scaRNA	15	187.40	2,811	0.000260
	CD-box	131.00	88.56	11,602	0.001074
	HACA-box	70	140.70	9,849	0.000911
	splicing	72	138.47	9,970	0.000923
	unknown	2	59.50	119	0.000011

Supplementary Table 16: Summary of annotation of repeat regions

	CAU_Silkie	
	Length(bp)	% in Genome
DNA transposons	11,500,736	1.06
LINE	79,641,014	7.37
SINE	678,831	0.06
LTR elements	25,368,163	2.35
Unclassified	10,688,751	0.99
Total interspersed repeats	127,877,495	11.83
Small RNA	252,827	0.02
Satellites	20,283,898	1.88
Simple repeats	21,320,180	1.97
Low complexity	4,082,301	0.38
Total	173,647,790	16.07

*The repeat regions was perormed using Repeamasker pipeline

Supplementary Table 17: Primer sequences for identifying coding sequence of silkie *leptin*

	Template	Forward (5′ to 3′)	Reverse (5′ to 3′)
leptin 1-1	cDNA	ATGGCCTTCCCCGTCCGCT	TTGCCTTCACATCCCCGAG
leptin 2-1	cDNA	GTGAAGCCCCCCCCCCCATCAC	ATCGCCTCAATCGTTGCTCT
leptin 2-2	cDNA	GCTGCAGAGCAACGATTGAG	GTTATCCAGGTCGCTTCGCA
leptin 2-3	cDNA	CGAAGCGACCTGGATAACCTT	GCTCCTTCAACTCAGGCTCC
leptin 2-4	cDNA	ATTGGAGCCTGAGTTGAAGG	AGCCCCACAACCACCCCCTC
leptin 2-5	cDNA	CTGGAGGAGGCGCCGCATAC	TTAACACCCCACCCCCCCTTC

Supplementary Table 18: Primer sequences to detect the expression levels of leptin and LEPR in Silkie chickens.

Gene	Orientation	Sequence
leptin	forward	5′- GGCTGCAGAGCAACGATTGA -3′
	reverse	5′- GCAAAGGTTATCCAGGTCGC -3′
chLEPR	forward	5′- CACTCGCTGGGAACACTTGA -3′
	reverse	5′- TTCAGCAGCCCATCGTTTCT -3′
GAPDH	forward	5′- CCCCCATGTTTGTGATGGGT -3′
	reverse	5′- ACGCTGGGATGATGTTCTGG -3′
