# Supplementary material for: A chromosome-level genome assembly for the Silkie chicken resolves complete sequences for key chicken metabolic, reproductive, and immunity genes
Source: Commun Biol. 2023 Dec 6;6:1233. doi: 10.1038/s42003-023-05619-y (PMC10700341; doi:10.1038/s42003-023-05619-y)
Supplement: Supplementary file 2 — Description of Additional Supplementary Files [file 42003_2023_5619_MOESM2_ESM.docx]

**Description of Additional Supplementary Files**

**File name:** Supplementary Data 1

**Description:** This supplementary dataset provides information on

**File name:** Supplementary Data 2

**Description:** The description of PAVs determined by one-tailed t-test between CAU_silkie and GGA7b.

**File name:** Supplementary Data 3

**Description:** Genes overlap with sequences whom absence in other seven chicken breeds.

**File name:** Supplementary Data 4

**Description:** The Structural variations detected between CAU_silkie and GGA7b.

**File name:** Supplementary Data 5

**Description:** Results of muanual annotation of protein-coding genes on chromosome 16 of Silkie and chromosome 30 of Mallard

**File name:** Supplementary Data 6

**Description:** Results of Differential Gene Expression in Chickens Before and After Infection with Marek's Disease

**File name:** Supplementary Data 7

**Description:** Results of Differential Gene Expression in Chickens Before and After Infection with E. coli Disease

**File name:** Supplementary Data 8

**Description:** The missing genes identified in the Silkie genome.

**File name:** Supplementary Data 9

**Description:** Missing genes with traces of presence in bird genomes.

**File name:** Supplementary Data 10

**Description:** Novel sequences that are not present in the red jungle fowl genome

**File name:** Supplementary Data 11

**Description:** Source data
