# Supplementary material for: A chromosome-level genome assembly for the Silkie chicken resolves complete sequences for key chicken metabolic, reproductive, and immunity genes
Source: Commun Biol. 2023 Dec 6;6:1233. doi: 10.1038/s42003-023-05619-y (PMC10700341; doi:10.1038/s42003-023-05619-y)
Supplement: Supplementary file 6 — Reporting Summary [file 42003_2023_5619_MOESM6_ESM.pdf]

nature portfolio

Corresponding author(s): Ning Yang, Zhuo-Cheng Hou, Li-Wa Shao

Last updated by author(s): Oct 29, 2023

Reporting Summary

Nature Portfolio wishes to improve the reproducibility of the work that we publish. This form provides structure for consistency and transparency in reporting. For further information on Nature Portfolio policies, see our Editorial Policies and the Editorial Policy Checklist.

Statistics

For all statistical analyses, confirm that the following items are present in the figure legend, table legend, main text, or Methods section.

n/a	Confirmed	
☐	☒	The exact sample size (*n*) for each experimental group/condition, given as a discrete number and unit of measurement
☐	☒	A statement on whether measurements were taken from distinct samples or whether the same sample was measured repeatedly
☐	☒	The statistical test(s) used AND whether they are one- or two-sided<br>*Only common tests should be described solely by name; describe more complex techniques in the Methods section.*
☒	☐	A description of all covariates tested
☒	☐	A description of any assumptions or corrections, such as tests of normality and adjustment for multiple comparisons
☐	☒	A full description of the statistical parameters including central tendency (e.g. means) or other basic estimates (e.g. regression coefficient) AND variation (e.g. standard deviation) or associated estimates of uncertainty (e.g. confidence intervals)
☐	☒	For null hypothesis testing, the test statistic (e.g. *F*, *t*, *r*) with confidence intervals, effect sizes, degrees of freedom and *P* value noted<br>*Give P values as exact values whenever suitable.*
☒	☐	For Bayesian analysis, information on the choice of priors and Markov chain Monte Carlo settings
☒	☐	For hierarchical and complex designs, identification of the appropriate level for tests and full reporting of outcomes
☐	☒	Estimates of effect sizes (e.g. Cohen's *d*, Pearson's *r*), indicating how they were calculated

Our web collection on statistics for biologists contains articles on many of the points above.

Software and code

Policy information about availability of computer code

Data collection	HiFiasm 0.13-r307, NextDenovo v2.3.1, Nextpolish v1.3.1, Quickmerge v0.3, SALSA2 v2.2, PBJelly2 PBSuite_15.8.24, TGS-gap-closer v1.1.1, Purge_dups v1.2.5, SALSA v2.2, Braker2 (v2.1.6), AUGUSTUS (v3.3.2), SNAP(v.2013-11-29),Trinity(v2.8.3),PASA (v2.3.3),tRNAscan-SE(v1.3.1),Infernal (v1.1.3), RepeatMasker(v4.0.7), Apollo (v2.6.1), EVidenceModeler (v2.31.8), ,LTR_FINDER (1.06), and RepeatModeler (v1.05)
Data analysis	HTSeq(v0.11.2),DEseq2(v1.32.0),Tophat2(v2.1.1),MG2C(v2.1), RectChr(v1.29), Nanopolish v0.13.2, HiCPlotter v0.6.6, Syri v1.5, HiC-Pro v3.0.0, BUSCO (version 5), BLAST v2.10.1+, SAMtools 1.7, Vcftools v0.1.16, Speedseq v0.1.2, MUMmer v4.00beta2, BEDTools v2.30.0, Delly v0.9.1, Freebayes v0.9.21, scoreTool v1.1. R v3.6.0, Seqtk v1.3-r116-dirty,IGV v2.11.4, RoseTTAFold, pyMOL(v2.5.2, , ZDOCK version: 2.3.2) and Seqkit v0.15.0

For manuscripts utilizing custom algorithms or software that are central to the research but not yet described in published literature, software must be made available to editors and reviewers. We strongly encourage code deposition in a community repository (e.g. GitHub). See the Nature Portfolio guidelines for submitting code & software for further information.

Data

Policy information about availability of data

All manuscripts must include a data availability statement. This statement should provide the following information, where applicable:

- Accession codes, unique identifiers, or web links for publicly available datasets
- A description of any restrictions on data availability
- For clinical datasets or third party data, please ensure that the statement adheres to our policy

The raw sequencing data, genomic sequencing data and transcriptome data that support the findings of this study have been deposited to the NCBI BioProject

database under accession PRJNA805080 and PRJNA827662. The sequence source of the public database were shown below: Uniprot database was downloaded from https://www.uniprot.org/; Ensembl/GENCODE gene set of human was downloaded from http://ftp.ensembl.org/pub/release-103/fasta/homo_sapiens/pep/Homo_sapiens.GRCh38.pep.all.fa.gz; Nr database was downloaded from https://ftp.ncbi.nlm.nih.gov/blast/db/FASTA/nr.gz; KEGG database was downloaded from https://www.genome.jp/kegg/; InterPro was downloaded from https://www.ebi.ac.uk/interpro/; Pfam database was downloaded from http://pfam.xfam.org/; GO database was downloaded from http://geneontology.org/. All data and research materials are available upon reasonable request by contacting the corresponding authors.

Field-specific reporting

Please select the one below that is the best fit for your research. If you are not sure, read the appropriate sections before making your selection.

☒ Life sciences ☐ Behavioural & social sciences ☐ Ecological, evolutionary & environmental sciences

For a reference copy of the document with all sections, see nature.com/documents/nr-reporting-summary-flat.pdf

Life sciences study design

All studies must disclose on these points even when the disclosure is negative.

Sample size	No sample-size calculation was performed, but the sample size chosen was compare to the pervious literatures similarly reporting genomic resources for interspecific difference. For instance, Zhu, F. et al. 2021 used 48 Pekin ducks, 43 mallard birds and 23 Shaoxing ducks for exploring interspecific difference. Laine, V et al. 2016 used 29 wild great tit individuals for exploring interspecific difference. Therefore, we think in our study ,the samples size is sufficient for genomic and transcriptomic analysis. Therefore, we think in our study ,the samples size is sufficient for genomic and transcriptomic analysis. Additionally,for immunoblotting experiments in cells, about 1 million cells per sample. For immunoprecipitation experiments in cells, cells in one 10cm dish with 90% confluence per sample. For mRNA-seq sample preparations, cells in one well of 6-well plate with about 90% cell confluence per sample were used to extract RNA. For RT-qPCR experiments in cells, one well of 6-well plate with about 90% cell confluence per sample. Sample size was determined according to our pre-tests. For RT-qPCR experiments in tissues, about 50-100 mg tissues per sample were used to extract RNA. Sample size was determined according to our pre-tests.
Data exclusions	Data was only excluded where experiments failed (based on animals or cells failed to meet experiment endpoint or variance of internal standards), or where limited material were exhausted.
Replication	Differentiation assays have been performed at least three times, and all attempt at replication were successfully (detail are indicated on figures/results). In RNA-seq analysis, each design has 6 biological replicates, which eliminates intra-group errors and improves the accuracy of the results. The sequencing data is 8G/sample to ensure that the sequencing is saturated. DNA re-sequencing sequencing depth is between 10-30x to ensure comprehensive and accurate detection of variation. We aimed to address all conclusions using different methods and replicated each experiment as written in the respective figure legends. Each experiment was repeated at least triple under independent conditions, all replicates showed similar results.
Randomization	All samples were randomly assigned to experimental groups. Animals were weight matched and randomly assigned to experimental groups. Cells in culture wells were randomly assigned to study groups. For immunoblotting, immunoprecipitation, qPCR experiments and mRNA-seq experiments, samples under each treatment condition were collected as standard approaches.
Blinding	Blinding was not used because all analyses in the main text were performed on the premise that the samples are known.

Reporting for specific materials, systems and methods

We require information from authors about some types of materials, experimental systems and methods used in many studies. Here, indicate whether each material, system or method listed is relevant to your study. If you are not sure if a list item applies to your research, read the appropriate section before selecting a response.

Materials & experimental systems

n/a	Involved in the study	
☐	☒	Antibodies
☐	☒	Eukaryotic cell lines
☒	☐	Palaeontology and archaeology
☐	☒	Animals and other organisms
☒	☐	Human research participants
☒	☐	Clinical data
☒	☐	Dual use research of concern

Methods

n/a	Involved in the study	
☒	☐	ChIP-seq
☒	☐	Flow cytometry
☒	☐	MRI-based neuroimaging

Antibodies

Antibodies used	1. Rabbit polyclonal anti-FLAG antibody for western blots: Supplier/Sigma; Cat. No./F7425; Lot No./0000120996.<br>2. Mouse monoclonal anti-Myc antibody for western blots: Supplier/Cell Signaling Technology; Cat. No./2276; Clone name/9B11; Lot No./24.

	3. anti-GAPDH antibody for western blots: Supplier/Proteintech; Cat. No./10494-1-AP; Lot No./00087635.<br>4. Mouse monoclonal anti-FLAG magnetic beads for immunoprecipitation: Supplier/Sigma; Cat. No./M8823; Clone name/M2; Lot No. /SLCJ0317.<br>5. Mouse monoclonal anti-Myc magnetic beads for immunoprecipitation: Supplier/Bimake; Cat. No./B26302; Clone name is not supplied by the supplier; Lot No./820023.<br>6. anti-mouse IgG antibody for western blots: Supplier/Abbkine; Cat. No./A25112, Lot No./ATUOC2801<br>7. anti-Rabbit IgG antibody for western blots: Supplier/Proteintech; Cat. No./SA00001-2, Lot No./20000339.
Validation	1. Rabbit polyclonal anti-FLAG antibody(Sigma F7425): Validated by the supplier with following notes: (1) species reactivity: all. (2) Application: The antibody recognizes the FLAG epitope located on FLAG-tagged fusion proteins, applying dot blot, WB, IP and ICC assays.<br>2. Mouse monoclonal anti-Myc antibody(Cell Signaling Technology 2276): Validated by the supplier with following notes (1) species reactivity: all. (2) Application: WB, IP, IHC, ChIP, IF, F, E-P.<br>3. anti-GAPDH antibody(Proteintech 10494-1-AP): Validated by the supplier with following notes: 10494-1-AP targets GAPDH in WB, RIP, IP, IHC, IF, FC, CoIP, ELISA applications and shows reactivity with human, mouse, rat, pig, chicken, arabidopsis , corn , cabbage, rice samples etc..<br>4. Mouse monoclonal anti-FLAG magnetic beads(Sigma M8823): Validated by the supplier with following notes: Specificity: Binding Specificity toward FLAG-tagged protein. Application: Suitable for immunoprecipitation procedures.<br>5. Mouse monoclonal anti-Myc magnetic beads(Bimake B26302): Validated by the supplier with following notes: Application: Immunoprecipitation and protein Purification. Immunoprecipitation of 293T cells was validated, Citation [PMID]: 31091447.

Eukaryotic cell lines

Policy information about cell lines

Cell line source(s)	A cell line of immortalized chicken preadipocytes (ICP1) was provided by the Poultry Breeding Group of the College of Animal Science and Technology, Northeast Agricultural University.<br>The chicken embryo fibroblast cell line (DF-1) were purchased from ATCC.
Authentication	Cell lines served in this study were not authenticated right before usage.
Mycoplasma contamination	Cell lines were validated to be free of mycoplasma contamination.
Commonly misidentified lines<br>(See ICLAC register)	No commonly misidentified cell lines were used

Animals and other organisms

Policy information about studies involving animals; ARRIVE guidelines recommended for reporting animal research

Laboratory animals	Silkie hens at 20 weeks of age were used to collecte DNA and RNA.
Wild animals	The study did not involve wild animals
Field-collected samples	The study did not involve the sample collected from the field
Ethics oversight	Animal Care and Use Committee of China Agricultural University (permit number: SYXK 2007–0023).

Note that full information on the approval of the study protocol must also be provided in the manuscript.
